# Amyotrophic Lateral Sclerosis: Proteins, Proteostasis, Prions, and Promises

**DOI:** 10.3389/fncel.2020.581907

**Published:** 2020-11-04

**Authors:** Luke McAlary, Yee Lian Chew, Jeremy Stephen Lum, Nicholas John Geraghty, Justin John Yerbury, Neil R. Cashman

**Affiliations:** ^1^Illawarra Health and Medical Research Institute, University of Wollongong, Wollongong, NSW, Australia; ^2^Molecular Horizons and School of Chemistry and Molecular Bioscience, Faculty of Science, Medicine and Health, University of Wollongong, Wollongong, NSW, Australia; ^3^Djavad Mowafaghian Centre for Brain Health, University of British Columbia, Vancouver, BC, Canada

**Keywords:** amyotrophic lateral scelerosis, proteostasis, protein aggregation, prion-like, *in vitro* models, invertebrate models, mouse models, therapeutics

## Abstract

Amyotrophic lateral sclerosis (ALS) is characterized by the progressive degeneration of the motor neurons that innervate muscle, resulting in gradual paralysis and culminating in the inability to breathe or swallow. This neuronal degeneration occurs in a spatiotemporal manner from a point of onset in the central nervous system (CNS), suggesting that there is a molecule that spreads from cell-to-cell. There is strong evidence that the onset and progression of ALS pathology is a consequence of protein misfolding and aggregation. In line with this, a hallmark pathology of ALS is protein deposition and inclusion formation within motor neurons and surrounding glia of the proteins TAR DNA-binding protein 43, superoxide dismutase-1, or fused in sarcoma. Collectively, the observed protein aggregation, in conjunction with the spatiotemporal spread of symptoms, strongly suggests a prion-like propagation of protein aggregation occurs in ALS. In this review, we discuss the role of protein aggregation in ALS concerning protein homeostasis (proteostasis) mechanisms and prion-like propagation. Furthermore, we examine the experimental models used to investigate these processes, including *in vitro* assays, cultured cells, invertebrate models, and murine models. Finally, we evaluate the therapeutics that may best prevent the onset or spread of pathology in ALS and discuss what lies on the horizon for treating this currently incurable disease.

## Introduction

### Proteostasis and Prion-Like Propagation

A major pathological component of neurodegenerative diseases such as Alzheimer’s disease, Parkinson’s disease, frontotemporal lobar degeneration (FTLD), and amyotrophic lateral sclerosis (ALS) is the prion-like propagation of misfolded and aggregated proteins in the central nervous system (CNS; for recent reviews in each case see—Hock and Polymenidou, 2016; Watts and Prusiner, 2018; McAlary et al., 2019b; Vargas et al., 2019). The idea of prion-like propagation of protein misfolding and aggregation in these diseases offers a plausible explanation for their idiopathic nature, progressive anatomical spread, the selective vulnerability of specific CNS regions, characteristic pathologies, and age-associated onset (Prusiner, 2001). In each disease, particular proteins are thought to misfold into a conformation that is capable of propagating throughout the CNS like the infectious prion protein (PrP; Vaquer-Alicea and Diamond, 2019).

PrP is typically found in a conformation composed primarily of alpha-helices (PrP^C^); however, it can be converted into a β-sheet-rich pathogenic conformation (PrP^Sc^) that can recruit and convert normal PrP to become pathogenic PrP^Sc^ through a template-directed manner (Prusiner, 1982; Pan et al., 1993). This conversion may be the result of aberrant biosynthesis and processing in cells (reviewed in Chakrabarti et al., 2009). There is no defined structure for PrP^Sc^ as it exists in a continuum from monomers to large amyloid-like polymeric assemblies (McKinley et al., 1991; Ceroni et al., 1996). Similar to PrP^Sc^, proteins implicated in the etiology of many neurodegenerative diseases are known to form amyloid *in vitro* and deposit into intracellular inclusions in an amyloid conformation (Chiti and Dobson, 2017). The amyloid state is defined by its cross-β fiber architecture (Eisenberg and Jucker, 2012; Eisenberg and Sawaya, 2017), and is of critical importance to the pathogenic mechanisms of prions and prion-like proteins.

**Figure 1:**
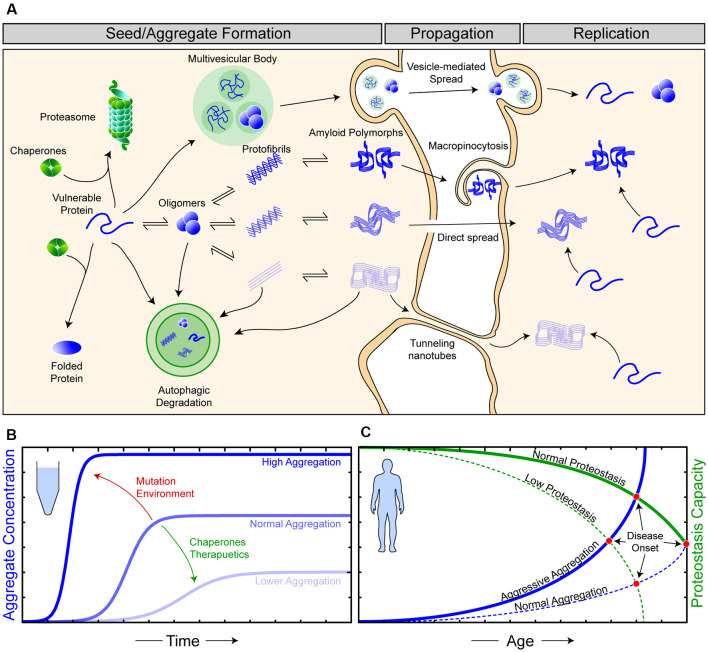
The relationship between proteostasis and prion-like protein propagation. **(A)** The proteostasis network (green objects) is composed of molecular chaperone proteins, degradation pathways (proteasomal and autophagic), and the trafficking of proteins. Chaperones act to protect vulnerable proteins from becoming misfolded and aggregating, potentially through the amyloid pathway (blue). During seed/aggregate formation, proteins vulnerable to amyloid aggregation can form polymorphic assemblies through template-directed growth, eventually, elicit different biological and pathological effects dependent on the polymorphic assembly (strain). Amyloid assemblies are thought to propagate from cell-to-cell through exocytosis in vesicles and exosomes, through membrane breakages, macropinocytosis, and tunneling nanotubes. Once an amyloid assembly has been transferred to a naive cell, replication continues as the amyloid assemblies can now recruit vulnerable protein within this cell. **(B)** *In vitro* experiments have shown that amyloid formation can be augmented *via* changes in environmental conditions or mutations (red) in substrate proteins to become more aggressive. Likewise, the addition of molecular chaperones and/or therapeutics (green), such as small molecules or antibodies, can suppress amyloid aggregation. **(C)** Amyloid aggregation in humans is a stochastic process, occurring over long time scales and, in simplistic terms, is an interplay of proteostasis capacity (green) and protein aggregation propensity (blue). Mutations and/or environmental features can result in both aggressive aggregation and/or a lower proteostasis capacity, ultimately resulting in earlier disease onset in affected individuals.

*In vitro*, the formation of amyloid is thought to proceed from the initial misfolding and aggregation of protein monomers into an ensemble of soluble non-native oligomeric states, with some oligomers forming proto-fibrils capable of elongating through template-directed monomer addition (Arosio et al., 2015). Amyloid fibrils are typically highly thermodynamically stable but vary in their mechanical stability (Yoon et al., 2013). Amyloid fibrils with low mechanical stability are more likely to fragment, leading to the exposure of more ends from which they can elongate in a repetitive cycle of fragmentation and growth (Knowles and Buehler, 2011; Figures 1A,B). Indeed, fragmentation is a highly important characteristic of the infectivity and replication propensity of prions and prion-like proteins (Marchante et al., 2017). Furthermore, microstructural heterogeneity in the initial population of misfolded protein and oligomers is thought to give rise to a range of conformationally distinct amyloid fibrils with different physical properties, often termed polymorphs (Petkova, 2005; Kodali et al., 2010; Safar et al., 2015). Over time, the fibril polymorphs with properties that permit efficient self-replication are selected for, giving rise to a dominant “strain” of amyloid fibril (Figure 1A). The notion of different strains of amyloid and prions provides a highly plausible explanation for the pathologic and clinical heterogeneity observed in neurodegeneration, as different strains can spread and recruit benign substrates at different rates (Telling et al., 1996; Safar et al., 1998; Morales, 2017). Supporting this, cryo-electron microscopy of amyloid isolated from human brain tissue has shown structural diversity in fibrils isolated from a single person (Kollmer et al., 2019) and in different neurodegenerative prion-like diseases (Fitzpatrick et al., 2017; Strohäker et al., 2019).

Although amyloid formation is often simplified as a relatively rapid kinetic reaction involving only the constituent amyloidogenic protein (Arosio et al., 2015; Figure 1B), the *in vivo* formation of these assemblies is notably slower and more complex (Owen et al., 2019). This additional complexity is primarily due to the existence of cellular mechanisms to maintain protein homeostasis (proteostasis; Yerbury et al., 2016; Figure 1A). Proteostasis is the concept of the maintenance of the proteome in the correct concentration (balance of protein synthesis and degradation), in the correct conformation (chaperones), and at the right location at the right time (trafficking). The importance of proteostasis in neurodegenerative disease is highlighted by the fact that disturbed proteostasis mechanisms are strongly associated with aging and neurodegeneration (Cheng et al., 2018; Kurtishi et al., 2019; Lehtonen et al., 2019; Yerbury et al., 2020). Mutations can predispose a protein to become more aggregation prone, and mutations in proteostasis components can impair proteostasis, where both are capable of leading to an earlier onset of neurodegenerative disease (Figure 1C). It is suggested that proteostasis capacity declines with age, with this being due to a combination of genetic and environmental factors (Hipp et al., 2019), making efforts into how determining proteostasis is maintained an important part of neurodegenerative disease research.

The key parts of the proteostasis network that are related to protein quality control are molecular chaperones and protein degradation. Molecular chaperone proteins are found throughout cell compartments, with the endoplasmic reticulum (ER) containing many chaperones due to the role it plays in protein synthesis. Protein disulfide isomerases (PDI) are a family of molecular chaperone proteins, localized primarily to the ER, that have oxidoreductase activity, which allows them to reduce and oxidize disulfide bonds in the proteins they are chaperoning (Hatahet and Ruddock, 2009). The important role of PDI’s in chaperoning newly synthesized proteins is signified by their growing involvement in several neurodegenerative diseases, including ALS (Perri et al., 2016). Another class of molecular chaperones are heat shock proteins, which act to prevent protein misfolding and aggregation by binding unstable proteins to either aid in their refolding or delivering them to cellular protein degradation machinery (Goldberg, 2003; Hartl et al., 2011; Balchin et al., 2016). Some molecular chaperone proteins are also suggested to aid in the mechanical stabilization of amyloid fibrils by binding along their solvent-exposed surfaces (Shammas et al., 2011; Binger et al., 2013; Cox et al., 2018). Molecular chaperones can even break apart amyloid assemblies (Baughman et al., 2018; Scior et al., 2018), making them easier to degrade or potentially promoting their propagation (Jones and Tuite, 2005). If a protein is terminally misfolded or aggregated, it is delivered to protein degradation machinery, such as the ubiquitin-proteasome system and autophagy-lysosomal system, for proteolytic degradation (Goldberg, 2003; Yerbury et al., 2016). The ubiquitin-proteasome degrades misfolded proteins (Kleiger and Mayor, 2014), but is not capable of dealing with larger aggregates, which are typically degraded, along with damaged cellular components, through autophagy (Dikic and Elazar, 2018).

The relationship between prion-like propagation and the proteostasis network is still being elucidated. Although the proteostasis network acts to suppress protein misfolding and aggregation, some prion-like particles eventually evade these defensive systems. Indeed, some prion-like strains may be resistant to neutralization by proteostasis mechanisms. Over time, these resistant strains could outcompete other strains, leading to the formation of the pathology observed post-mortem. It may well be that the polymorphic amyloid assemblies currently being identified *ex vivo* (Fitzpatrick et al., 2017; Kollmer et al., 2019) represent those that are most capable of inducing disease and, therefore, may present as viable therapeutic targets in the face of the extreme patient heterogeneity observed in neurodegenerative diseases. It is also possible that dynamic prion-like propagation of misfolded proteins may place further stress on the proteostatic machinery, resulting in additional errors or even collapse (Yerbury et al., 2016). A combined understanding of proteostasis and the prion-like mechanisms involved in some conditions could potentially shed further light on pathogenesis. Therefore, this review focuses on the examination of both proteostasis and prion-like propagation in amyotrophic lateral sclerosis, a disease in which there is strong evidence to suggest a combination of prion-like propagation and dysfunctional proteostasis is occurring simultaneously.

### Amyotrophic Lateral Sclerosis—Pathology and Genetics Related to Prion-Like Propagation and Proteostasis

Amyotrophic lateral sclerosis (ALS) is characterized by the progressive degeneration of motor neurons in both the motor cortex, brain stem, and spinal cord (Van Es et al., 2017). Motor symptoms have been observed to occur from a focal point of onset from which proximal motor neurons become affected in an orderly manner (Ravits et al., 2007; Ravits and La Spada, 2009). The site of onset can be highly heterogeneous, resulting in varied symptom presentation, which ultimately leads to difficulty in making a correct diagnosis (Chiò et al., 2009a). Pathology is also thought to begin at a focal point within the CNS and, like other neurodegenerative diseases, is associated with the deposition of proteins into insoluble inclusions. ALS-associated inclusions are found primarily within motor neurons, but can also form in the surrounding oligodendrocytes and astrocytes (Mori et al., 2008; Zhang et al., 2008; Philips et al., 2013; Brettschneider et al., 2014; Fatima et al., 2015) as well as cell populations outside the pyramidal motor system (Geser et al., 2008; Ince et al., 2011; Braak et al., 2013; Brettschneider et al., 2013). Histopathological studies have suggested that the spread of pathology from a focal point occurs through connected cells in stages (Mori et al., 2008; Brettschneider et al., 2013), similar to other neurodegenerative diseases and prion diseases.

The key proteins associated with pathological inclusion formation are transactive response DNA-binding protein 43 (TDP-43; Arai et al., 2006; Neumann et al., 2006), superoxide dismutase-1 (SOD1; Shibata et al., 1993), and fused in sarcoma (FUS; Kwiatkowski et al., 2009; Vance et al., 2009). A significant majority of ALS cases (~97%) are associated with TDP-43 positive inclusions, where the remainder are associated with either SOD1 (~2%) or FUS (~1%) inclusions (Ling et al., 2013). TDP-43 is a DNA/RNA-binding protein that has many functions in RNA metabolism (Buratti et al., 2001, 2005; Hefferon et al., 2004; Mercado et al., 2005). FUS is also a DNA/RNA-binding protein that has functions related to both RNA metabolism (Belly et al., 2005; Fujii and Takumi, 2005; Andersson et al., 2008) and the DNA-damage response (Wang W.-Y. et al., 2013). SOD1 is the primary antioxidant enzyme of the cell and functions to convert superoxide anion to less harmful molecular oxygen or hydrogen peroxide (Mccord and Fridovich, 1969). Importantly, TDP-43, SOD1, and FUS are capable of forming amyloid fibrils *in vitro* (Chattopadhyay et al., 2008; Chen et al., 2010; Nomura et al., 2014), and there is also strong evidence to suggest that, for at least SOD1 and TDP-43, they adopt an amyloid formation *in vivo* (Kato et al., 2000; Bigio et al., 2013; Robinson et al., 2013), although there is not yet a consensus as classical amyloid stains are often negative in ALS cases (Neumann et al., 2006; Kerman et al., 2010).

The most common identified cause of fALS cases is hexanucleotide (G_4_C_2_) repeat expansions in the *C9ORF72* gene, which result in the transcription of large (tens to thousands of repeats) hexanucleotide repeat RNA molecules and subsequent repeat-associated non-AUG (RAN) translation into dipeptide repeat (DPR) proteins (Freibaum and Taylor, 2017). The DPRs translated from the G_4_C_2_ repeat RNA include Gly-Arg (GA), Pro-Gly (PG), Pro-Arg (PR), Gly-Ala (GA), and Pro-Ala (PA) polypeptides. These DPRs are thought to elicit different levels of toxicity and react differently within the cell based on their physicochemical properties (Freibaum and Taylor, 2017). For example, PR and GR repeats are suggested to aberrantly interact with RNA and nucleolar proteins, resulting in disruption to ribosomal biogenesis (White et al., 2019). On the other hand, GA repeats have been suggested to bind and trap multiple proteins responsible for the trafficking of biomolecules between the nucleus and cytoplasm (Zhang et al., 2016). Regardless of the effects of the DPRs, the major pathological hallmark of C9ORF72-associated ALS remains TDP-43 mislocalization and aggregation (Cook et al., 2020).

Although TDP-43 mislocalization, phosphorylation, and aggregation in motor neurons is the major pathological hallmark of ALS, few patients carry TDP-43 mutations. Similar to most other neurodegenerative diseases and prion diseases, the majority of ALS cases are sporadic (sALS), accounting for roughly 90% of cases. The remaining cases are familial (fALS) and, although some of the underlying genetic causes remain unidentified, are typically associated with a family history of the disease (Taylor et al., 2016). Only ~5% of patients with fALS carry TDP-43 mutations, SOD1 accounts for ~20% of fALS cases, and FUS accounts for ~5% (Laferriere and Polymenidou, 2015). The identification of hexanucleotide repeats in *C9ORF72* as being ALS-causative was a significant discovery to the field as these mutations account for approximately 40% of fALS cases (Dejesus-Hernandez et al., 2011; Renton et al., 2011). Importantly, over 20 fALS-associated genes have been identified, where these genes are broadly associated with proteostasis mechanisms including protein degradation, protein production (RNA metabolism), and protein trafficking (Taylor et al., 2016; Yerbury et al., 2020). Comprehensive reviews of the genetics and resulting pathomechanisms of ALS have been reviewed elsewhere (Matus et al., 2013; Renton et al., 2014; Carrì et al., 2015; Medinas et al., 2017b; Nguyen et al., 2018; Burk and Pasterkamp, 2019; Mejzini et al., 2019; Vicencio et al., 2020).

A brief example of the complexity of ALS genetics and mechanisms can be found with ubiquilin-2 (UBQLN2). UBQLN2 is an fALS-associated protein responsible for many interactions that facilitate protein degradation *via* the proteasomal and autophagic pathways (Kleijnen et al., 2000; Kim et al., 2008; N’Diaye et al., 2009). As such, pathogenic mutations in ubiquilin-2 can result in dysfunctional protein degradation (Deng et al., 2011; Chang and Monteiro, 2015; Osaka et al., 2016; Renaud et al., 2019). However, UBQLN2 may also play a role in the dissolution of the cytoplasmic ribonucleoprotein complexes known as stress granules (Dao et al., 2018), implicating dysfunctional RNA metabolism for UBQLN2 mutants. Furthermore, mutations in genes such as *UBQLN2* may exacerbate the already metastable supersaturated states of TDP-43, SOD1, and FUS (Ciryam et al., 2017), hence, leading to the downstream pathological aggregation of these proteins in particular.

Collectively, the pathological aggregation and prion-like spread of specific proteins as a response to numerous potential mutations in the proteostasis network highlights the critical relationship between proteostasis and prion-like propagation in ALS. As such, researchers have exploited various experimental models to investigate these mechanisms in disease, including the *in vitro* study of purified proteins and cultured cells, as well as *in vivo* in invertebrate models (*Caenorhabditis elegans, Drosophila melanogaster*) and mouse models. For reviews that incorporate zebrafish (*Danio rerio*) models, see Babin et al. (2014), Van Damme et al. (2017), and Morrice et al. (2018). Here, we focus specifically on models that have used either SOD1, TDP-43, or FUS.

## *In Vitro* Models to Examine Proteostasis and Prion-Like Features of Amyotrophic Lateral Sclerosis

### Purified Protein Systems

Much of the knowledge that forms the foundation of our understanding of proteostasis collapse, and how this may result in the exponential replication of a prion-like particle in disease, has been supported and initiated by studies of purified protein. The simplistic and reductionist nature of experiments using purified protein affords significant advantages in the specific elucidation of protein structure and function. Through precise control and modulation of the environment (temperature, pH, oxidative/reducing, osmolyte concentration, denaturants) and the proteins (post-translational modification, mutation, binding partners, chaperones) in an assay, a more fundamental understanding of the processes underlying protein misfolding and prion-like propagation can be achieved.

#### Superoxide Dismutase-1

As a result of being the first protein found to harbor ALS-associated mutations (Rosen et al., 1993), SOD1, in its purified form, has been extensively studied from the perspectives of protein folding, aggregation, and prion-like propagation (McAlary et al., 2019b; Wright et al., 2019; Trist et al., 2020). SOD1 protein is most often expressed and purified using either bacteria (*E. coli*) or yeast (*S. cerevisiae*, Hallewell et al., 1985, 1987). Considering the extensive post-translational modification (PTM) that SOD1 must undergo before reaching its native conformation, which includes metal-binding, disulfide formation, and N-terminal acetylation, yeast are the expression vector of choice as this system is capable of imparting all of these PTMs (Hallewell et al., 1987). A bacterial expression strategy to facilitate correct metal input and disulfide formation is to co-express SOD1 with its co-chaperone the copper chaperone for SOD1 (CCS) in the presence of excess copper and zinc (Lindberg et al., 2002).

A SOD1 monomer is an 8-stranded β-barrel with two major loops, the metal-binding loop (loop IV) and electrostatic loop (loop VII), being responsible for the coordination of metals or guidance of superoxide substrate to the enzymatic site, respectively. Furthermore, a conserved intramolecular disulfide, that stabilizes the tertiary structure and promotes dimerization, is formed between residues Cys57 and Cys146 (Wright et al., 2019). The native conformation of SOD1 is an impressively stable homodimer, evidenced by resistance to thermal denaturation and proteolytic digestion (Senoo et al., 1988; Rodriguez et al., 2002). For this reason, SOD1 homodimers are sometimes used as a non-aggregating control protein in some assays (Gregory et al., 2017). Although the native conformation of SOD1 is highly stable, the maturation (sequential acquisition of post-translational modifications) folding landscape of SOD1 is highly susceptible to destabilization and off-folding pathways, especially when mutated (Lindberg et al., 2002; Rodriguez et al., 2002; Luchinat et al., 2014, 2017; Sekhar et al., 2015, 2016; McAlary et al., 2016; Culik et al., 2018). Given that ALS-associated mutations in SOD1 were found to elicit a gain-of-toxic function effect (Bruijn et al., 1998), and that protein misfolding and aggregation is a key hallmark of SOD1-associated ALS (Durham et al., 1997; Shinder et al., 2001), much work has focused on the effect that ALS-associated mutations had on SOD1 folding stability (Wright et al., 2019). In particular, studies have focused on dissecting the differences between mutant and wild-type SOD1 across its maturation landscape, with a focus on monomer stability (Lindberg et al., 2005; McAlary et al., 2016) and dimer stability (Lindberg et al., 2005; Redler et al., 2011; McAlary et al., 2013; Capper et al., 2018; Chantadul et al., 2020). Using both wild-type and variants of SOD1 with its free cysteines replaced (Lepock et al., 1990), SOD1 folding and misfolding has been rigorously probed *via* circular dichroism (Lindberg et al., 2002, 2005; Byström et al., 2010; Wright et al., 2013), nuclear magnetic resonance (Arnesano et al., 2004; Sekhar et al., 2015, 2016; Culik et al., 2018), and calorimetry (Rodriguez et al., 2002; Broom et al., 2015, 2016; Anzai et al., 2017).

Some evidence, however, shows that certain ALS-associated mutations, such as D101N, do not significantly alter the folding stability of the SOD1 maturation states that are thought to be likely disease precursors (Byström et al., 2010; Vassall et al., 2011). Indeed, some mutations confound attempts to directly relate folding stability to the variable patient survival observed for patients with different SOD1 mutations (Byström et al., 2010; Vassall et al., 2011; McAlary et al., 2016). More recently, the role of macromolecular crowding in tuning protein folding stability has become clearer. Macromolecular crowding refers to a high concentration of macromolecules (proteins, nucleic acids, lipids, carbohydrates) in solution. Contrary to previous ideas, macromolecular crowding can destabilize proteins that are typically considered as stable when assessed at dilute concentrations (Miklos et al., 2011; Sarkar et al., 2012). SOD1 is no exception to this as evidence is emerging that even wild-type SOD1, in its metal-free and/or reduced form, is destabilized at a physiological temperature under crowded conditions (Bille et al., 2019; Takahashi et al., 2020). Indeed, in-cell nuclear magnetic resonance experiments of SOD1 have revealed that destabilization is common to both wild-type and ALS-associated mutants (Luchinat et al., 2014; Danielsson et al., 2015). In regards to both proteostasis and prion-like propagation, the consensus of this work suggests that ALS-associated mutations can have specific destabilizing effects on SOD1 structure, resulting in a greater proportion of the protein populating an ensemble of non-native states and, therefore, is susceptible to aggregation and prion-like conversion (McAlary et al., 2019b; Wright et al., 2019). Furthermore, it would be interesting to revisit the biophysical studies of the effects of ALS-associated mutations on SOD1 folding stability with our greater understanding of the effect of macromolecular protein crowders. By better understanding the effects of protein crowding on SOD1 stability, we may better understand how specific mutations promote cytotoxic aggregation and the relationship between SOD1 variant stability and patient phenotype.

Similar to the above-mentioned studies of SOD1 folding stability, the examination of the fibrillation of SOD1 has seen extensive use of purified protein. Due to the high stability of SOD1, some assays have induced fibrillation of purified protein using harsh thermal or chemical conditions (DiDonato et al., 2003; Stathopulos et al., 2003); however, the most physiologically relevant assays have simply utilized chelating and reducing conditions to promote SOD1 fibrillation (Chattopadhyay et al., 2008). Typical SOD1 aggregation assays induce the fibrillation of the protein under shaking conditions and measure an increase in β-sheet content using Thioflavin-T (Naiki et al., 1989). Shaking is considered to increase the rate at which fibrils fragment to create new ends from which to polymerize. Although the fibrillation of SOD1 has been described as being mostly fragmentation-assisted *in vitro* and *in vivo* (Lang et al., 2015), a recent study reported the formation of SOD1 fibrils under quiescent conditions (Khan et al., 2017), suggesting alternative pathways by which SOD1 can fibrillate and that certain strains may be preferentially selected depending on the method used. Using the Thioflavin-T assay, it has been shown that even wild-type SOD1, in its demetallated and reduced form, is capable of forming amyloid fibrils (Chattopadhyay et al., 2008; Furukawa et al., 2008; Lang et al., 2012; Chan et al., 2013; Ivanova et al., 2014; Abdolvahabi et al., 2016; McAlary et al., 2016). Furthermore, ALS-associated mutations augment the fibrillation of SOD1 to different degrees (Furukawa et al., 2008, 2010; Lang et al., 2012; Chan et al., 2013; Abdolvahabi et al., 2016, 2017; McAlary et al., 2016), whereas some *de novo* mutations and PTMs have been shown to prevent (Abdolvahabi et al., 2015; Rasouli et al., 2017; Pokrishevsky et al., 2018) or enhance fibrillation (Shi et al., 2013). The Thioflavin-T assay has also been used to examine if SOD1 fibrillation can be lowered using chaperone proteins (Yerbury et al., 2013) or small molecules (Bhatia et al., 2015, 2020: Malik et al., 2019).

N-terminal acetylation of α-synuclein is reported to significantly alter the secondary structure of the protein (Trexler and Rhoades, 2012) and therefore, its aggregation kinetics and structure (Guerrero-Ferreira et al., 2019; Watson and Lee, 2019). Importantly, many of the studies examining SOD1 fibrillation have utilized bacterially expressed protein, which is not N-terminally acetylated like it is typically found in eukaryotic systems. Although no direct comparisons have been performed between N-terminally acetylated SOD1 and non-acetylated SOD1, there are some notable differences in the identified cores of the amyloid fibril structures produced by the two forms (Furukawa et al., 2010; Chan et al., 2013). There is currently no high-resolution structure of SOD1 fibrils composed of the full-length protein, unlike tau (Fitzpatrick et al., 2017), α-synuclein (Li B. et al., 2018), and amyloid-β (Schmidt et al., 2015). Current high-resolution structures of SOD1-related fibrils are restricted to synthetically produced peptides of small amyloidogenic regions of the protein (Ivanova et al., 2014; Sangwan et al., 2017). The amyloidogenic regions of SOD1 are _14_VQGIINFE_21_, _30_KVWGSIKGL_38_, _101_DSVISLS_107_, and _147_GVIGIAQ_153_ (Ivanova et al., 2014). However, limited proteolysis of fibrils produced from recombinant SOD1 has identified the N-terminus of the protein, comprising residues 1–63, as the potential amyloidogenic core (Furukawa et al., 2010; Chan et al., 2013). Other regions of the SOD1 molecule have also been detected, suggesting polymorphism in the fibril structures generated. It would be valuable to determine the structure of SOD1 fibril polymorphs to understand better the relationship between SOD1 aggregation and the prion-like spread observed in patients.

Protein fibrillation assays have been a key tool in understanding the underlying prion-like propagation of SOD1 aggregation. The presence of seed in fibrillation assays essentially bypasses the need for the nucleation of a fibrillation-competent protein assembly in the assay, thus shifting the kinetics almost entirely to fibril elongation (Cohen et al., 2011). Through the addition of preformed protein aggregates (from recombinant sources, cells, or whole organisms) to fibrillation assays, it can be elucidated whether or not the seed is present within a sample (Schmitz et al., 2016), the susceptibility of protein variants to seeded fibrillation (Pokrishevsky et al., 2018), or the ability of different treatments to prevent seeded fibrillation (Sievers et al., 2011). Indeed, the first evidence that SOD1 may have prion-like properties first came from fibrillation assays of recombinant purified SOD1 seeded with spinal cord extracts from mice overexpressing human SOD1-G93A (Chia et al., 2010). From seeded fibrillation assays, it has been determined that loss of the intramolecular disulfide is a significant contributor to increasing the susceptibility of SOD1 to undergo seeded fibrillation (Chattopadhyay et al., 2015) and that this is enhanced by mutation. Other studies examining the seeded fibrillation of SOD1 have shown that some *de novo* mutants of SOD1 are resistant to seed-induced fibrillation (Pokrishevsky et al., 2018), and that specific amyloidogenic segments of SOD1 contribute more significantly to seeded aggregation (Ivanova et al., 2014).

Overall, it is clear that ALS-associated mutations in SOD1 lower the folding stability of the protein and permit the sampling of more aggregation-prone conformations. These destabilized conformations are capable of both nucleating the formation of amyloid fibrils and acting as a substrate for the further polymerization of existing fibrils. Future inquiries in this area focusing on the elucidation of the structure and polymorphism of SOD1 fibrils would be useful to not only understand the fibrillation of SOD1 but also to design therapies that may prevent it. Besides, advances in the sensitivity of detecting fibrillar species with prion-like properties from clinical sources using fibrillation assays (also called the real-time quaking-induced conversion assay; Schmitz et al., 2016) is currently unexplored for SOD1-associated ALS and ALS in general. Application of the sensitivity of the Thioflavin-T-based fibrillation assays to ALS patient tissue would be invaluable in elucidating the relationship between patient prognosis and the prion-like nature of the disorder.

#### Transactive Response DNA-Binding Protein 43

There has been less research performed utilizing recombinant purified TDP-43, which is primarily due to the difficulty in purifying soluble TDP-43, and the difficulty handling the purified product. Some advances have recently been made in this space with the usage of solubility tags, replacement of tryptophan residues with alanine, and exhaustive processing of recombinant protein extracts (Li et al., 2017; Vivoli Vega et al., 2019; Wright et al., 2020). Owing to the difficulty associated with purification of the full-length protein, the biophysical understanding of TDP-43 has been obtained predominantly by analyses of purified single domains of the protein, including the N-terminal domain (NTD; Kuo et al., 2009; Mompeán et al., 2016), RNA-recognition motifs (RRM1 and RRM2; Kuo et al., 2009; Lukavsky et al., 2013), and the C-terminal low-complexity domain (LCD; Conicella et al., 2016). The intrinsically high aggregation propensity of the LCD is likely why the examination of full-length TDP-43 has been difficult, as other domains are reported to be soluble when expressed in heterologous systems (Kuo et al., 2009; Lukavsky et al., 2013; Mompeán et al., 2016). As well as affording TDP-43 a high propensity to aggregate, the LCD is also the domain that contains the majority of ALS/FTLD-associated mutations (Abel et al., 2013) and forms the major component by mass of the ALS/FTLD-associated fragments of TDP-43 (Neumann et al., 2006). As such, the LCD has been a primary focus of TDP-43-focused ALS/FTLD research.

The TDP-43 LCD is defined as a prion-like domain due to its enrichment in asparagine, glutamine, glycine, and tyrosine residues, making it similar in sequence composition and physicochemical properties to the yeast prion protein Sup35 (King et al., 2012). Recent studies have highlighted the capability of low-complexity domains in DNA/RNA-binding proteins (not all of them prion-like) to facilitate the formation of physiologically relevant biomolecular condensates through the process of liquid-liquid phase separation (LLPS) *in vitro* (Banani et al., 2017). Evidence for *in vivo* formation and dysfunction of biomolecular condensates is starting to emerge (Zhang et al., 2020). The TDP-43 LCD has been shown to undergo LLPS, with this being primarily mediated by a transiently-populated α-helix (residues 321–330; Conicella et al., 2016) as well as aromatic residues spread throughout the domain (Li H.-R. et al., 2018). The majority of ALS/FTLD-associated mutations in TDP-43 are localized to the LCD. Considering this, a recent effort has been applied to examining the role of biomolecular condensates, such as stress granules, as a potential site in which the initial aggregation of TDP-43 (and other RNA-binding proteins) may occur (reviewed in Nedelsky and Taylor, 2019). *In vitro* evidence suggests that LLPS of the TDP-43 LCD promotes amyloid fibril formation, likely at the condensate-solution interface (Babinchak et al., 2019) however, whether these fibrils are formed within condensates remains unclear. It is thought that disease-associated mutations may exacerbate the gradual loss of internal molecular rearrangement within condensates and promote a liquid-to-solid transition of the protein components, although this hypothesis is currently not fully tested, especially in regards to amyloid formation, and requires further investigation in both protein only assays and living cells.

Although the relationship between TDP-43 LCD LLPS and aggregation is not well understood, the capacity of the TDP-43 LCD, and smaller segments of it, to form amyloid fibrils *in vitro* is well-established (Chen et al., 2010; Guo et al., 2011; Jiang et al., 2013, 2016; Mompeán et al., 2014; Sun C.-S. et al., 2014; Zhu et al., 2014; Babinchak et al., 2019; Cao et al., 2019). Cross-β fibers are formed by several small segments of the TDP-43 LCD (Guenther et al., 2018a) which may comprise the cores of amyloid polymorphs formed by larger segments and the entire LCD itself (Chen et al., 2010; Guo et al., 2011; Jiang et al., 2013, 2016; Mompeán et al., 2014; Babinchak et al., 2019; Cao et al., 2019; Shenoy et al., 2020). Cryo-electron microscopy has been utilized to examine the polymorphic nature of synthetic TDP-43 LCD segment assemblies composed of amino acid residues 311–360 or 286–331 (Cao et al., 2019). Although these structures are the result of *in vitro* assays of LCD segments, they provide a starting point by which to understand the amyloid state of TDP-43 and how it might confer different pathogenic severities in patients. Indeed, it is clear that TDP-43 can adopt an amyloid-like state in relevant tissues from ALS and FTLD patient samples as shown by immunoelectron microscopy (Lin and Dickson, 2008; Thorpe et al., 2008) and Thioflavin-S staining of carefully processed samples (Bigio et al., 2013; Robinson et al., 2013). It is important to note that confirmation of amyloid in tissue samples has classically relied upon Thioflavin-S and Congo-red staining, however, evidence has emerged that in some cases protein fibrils may not effectively bind these dyes (Wang Y.-T. et al., 2013; Rasouli et al., 2017), making electron microscopy the tool of choice to definitively assess aggregate morphology (amorphous or fibrillar).

Unlike the LCD, the TDP-43 NTD is a well-structured and stable domain (Qin et al., 2014; Mompeán et al., 2016) that is capable of undergoing extensive multimerization (Chang et al., 2012) and polymerization (Afroz et al., 2017; Mompeán et al., 2017; Tsoi et al., 2017; Wang et al., 2018) *in vitro*. These biophysical studies, using measures of stability, protein-protein interactions, and structural techniques, have suggested several roles for the NTD in both TDP-43 function and pathology. Namely, the NTD promotes the formation of TDP-43 dimers and multimers, likely through head-to-tail interactions (Afroz et al., 2017; Wang et al., 2018; Wright et al., 2020; although there is a dispute about the contact interfaces). The NTD-mediated interaction of separate TDP-43 molecules may prevent the interaction of the aggregation-prone LCDs between interacting TDP-43 molecules (Afroz et al., 2017), suggesting a protective role for the NTD in ALS/FTLD. This notion of NTD-derived protection is supported by the cleavage of the NTD to give rise to C-terminal pathological fragments of TDP-43 in ALS/FTLD (reviewed in Berning and Walker, 2019). In contrast, others have suggested that NTD-mediated interactions promote the association and subsequent aggregation of the TDP-43 LCD (Tsoi et al., 2017).

Similar to the NTD and LCD, the biophysical study of the TDP-43 RRMs has resulted in furthering the understanding of how TDP-43 may gain pathogenic properties through misfolding. A key finding from biophysical analyses of purified RRM domains or full-length TDP-43 is that DNA/RNA-binding to the RRMs increases their stability and decreases the propensity of TDP-43 to aggregate (Huang et al., 2013; Sun Y. et al., 2014; Zacco et al., 2018, 2019). This information highlights the importance of the RRMs in understanding TDP-43 proteinopathy and also provides a means for which potential therapies may be explored by using DNA/RNA oligos to stabilize cytoplasmic TDP-43, a strategy that is currently being pursued (Mann et al., 2019). However, some care must be taken, as the disease-associated D169G mutation that occurs within RRM1 of TDP-43 has been shown to increase the stability of the domain but make TDP-43 more susceptible to caspase-mediated cleavage (Chiang et al., 2016). It is currently unknown what effect RNA/DNA-binding has on the proteolytic cleavage of TDP-43. RRM2 of TDP-43 has also been examined concerning its potential amyloidogenic properties, with data suggesting that it may play an active role in TDP-43 aggregation and amyloid formation. Most studies of the amyloidogenic properties of TDP-43 RRM2 have used small synthetic peptides of the domain (Saini and Chauhan, 2011, 2014; Wang Y.-T. et al., 2013; Shimonaka et al., 2016; Guenther et al., 2018b), but some work has investigated the ability of the entire RRM2 domain to form amyloid (Shimonaka et al., 2016; Garnier et al., 2017).

Collectively, this evidence supports a role for RRM2 in the fibrillation of TDP-43, however, there are still unanswered questions. The use of small peptides, whilst informative, is not suitable for the examination of the heterotypic interactions that typically underlie the formation of diverse fibril polymorphs (Fitzpatrick et al., 2017; Li B. et al., 2018; Guerrero-Ferreira et al., 2019; Kollmer et al., 2019). Considering the number of potential amyloidogenic regions proposed in the TDP-43 LCD (Guenther et al., 2018a), and potential amyloid properties of RRM2, it may well be that the number of possible fibril polymorphs formed by heterotypic interactions within TDP-43 is substantial. Furthermore, the disease-associated truncation of TDP-43 into C-terminal fragments may further diversify the amyloid structures that it can form. This being said, small peptides do give the advantage of allowing for the examination of the seeding properties of highly structurally pure amyloid samples. This has been demonstrated by using fibrils formed from small segments of the TDP-43 LCD to seed TDP-43 aggregation in cultured overexpressing TDP-43 transgenes (Shimonaka et al., 2016).

#### Fused in Sarcoma

Like TDP-43, purified FUS protein has not been as well explored compared to studies of purified SOD1. The N-terminus of FUS is a functional prion-like domain, followed by several RGG rich regions encompassing a single RRM, a zinc finger domain, and finally a nuclear localization signal at the far C-terminus (Iko et al., 2004). Most purification strategies involve the expression of FUS in bacteria as a fusion to tags (sometimes fluorescent and/or solubilizing) on either the N- or C-terminus (Han et al., 2012; Kato et al., 2012; Kwon et al., 2013; Nomura et al., 2014; Patel et al., 2015; Monahan et al., 2017; Alberti et al., 2018; Hofweber et al., 2018; Maharana et al., 2018; Qamar et al., 2018; Murthy et al., 2019).

Also similar to TDP-43, a focus has been placed on the ability of FUS to undergo LLPS to form biomolecular condensates or hydrogels (Han et al., 2012; Kato et al., 2012; Kwon et al., 2013; Patel et al., 2015; Monahan et al., 2017; Alberti et al., 2018; Hofweber et al., 2018; Maharana et al., 2018; Qamar et al., 2018; Murthy et al., 2019). The ability of FUS to undergo LLPS *in vitro* can be enhanced by the ALS-associated G156E mutation (Patel et al., 2015), or it can be suppressed by chaperones and/or post-translational modifications (Monahan et al., 2017; Hofweber et al., 2018; Qamar et al., 2018). Concerning proteostasis, these findings suggest that the aberrant condensation of FUS, or simply condensation of FUS disease mutants, may result in the formation of FUS aggregates in disease. Indeed, FUS condensates are suggested to solidify (mature) over time and also perhaps form larger fibrous structures (Patel et al., 2015), although some work has suggested that careful control of *in vitro* conditions (removal of bubbles, usage of unscratched surfaces) prevents excessive FUS fibril formation in condensation experiments *in vitro* (Alberti et al., 2018).

**Figure 2:**
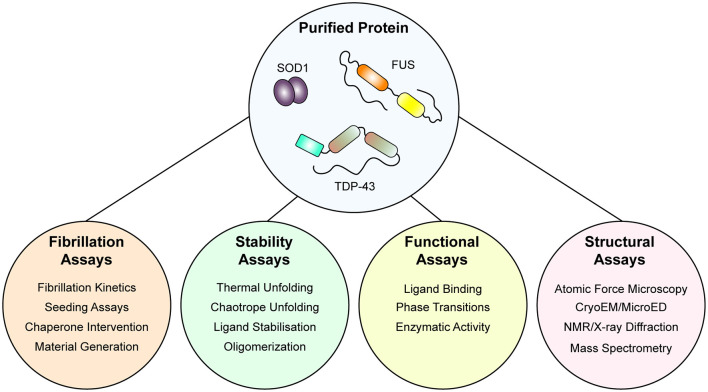
An integrative approach using purified protein to assaying the mechanisms of protein unfolding, aggregation, and prion-like behavior. Once purified, both wild-type and mutant protein is amenable to a suite of assays that can report on folding stability, function, fibrillation, and structure. Application of such techniques has allowed for the determination of the effect of the mutation on protein stability and fibrillation, the role of specific domains in fibrillation, and even the structure of fibrils themselves.

Indeed, current understanding of ALS as a prion-like disorder is supported strongly by experimentation of both SOD1 and TDP-43, however, experimentation with FUS is in early stages (McAlary et al., 2019b). Experiments examining only smaller regions of FUS, including the RGG-rich regions and the N-terminal prion-like domain, have established the ability of FUS to form labile amyloid-like fibers, distinguished from typical amyloid by their lower thermal stability (Kato et al., 2012; Murray et al., 2017; Hughes et al., 2018). Also, initial experiments examining the amyloidogenicity of purified FUS found that GST-tagged full-length FUS protein showed very rapid increases in solution turbidity, suggesting that LLPS was potentially occurring here rather than aggregation (Nomura et al., 2014). Nomura et al. (2014) also show GST-tagged FUS-G156E samples were Thioflavin-T positive and that fibrils were present by electron microscopy, but it is unclear if these were labile amyloid-like structures or *bona fide* highly stable amyloid. In the future, experiments designed to examine if a population of the amyloid assemblies formed by purified FUS (whether through LLPS or simple agitation assays) is resistant to detergent denaturation and proteolytic degradation would be useful to determine if purified FUS can form pathogenic amyloids. For a visual summary of the assays that can be performed with purified protein, see Figure 2.

### Cultured Cells

Although studies utilizing purified protein have provided substantial insight into the misfolding, chaperoning, and amyloid aggregation of ALS-associated proteins, there is a significant lack of biological complexity in these systems. Cultured cells, in the form of immortalized cell lines, have formed a foundation for understanding proteostasis and prion-like propagation in ALS/FTLD. The types of systems used are diverse in their design and application, so we have elicited to focus primarily on those systems that have best helped understand protein homeostasis and prion-like propagation within the cellular environment (reviews on iPSC culture modeling of ALS see Guo et al., 2017; Van Damme et al., 2017).

Standard cell lines in any biological experimentation involving ALS/FTLD often include human embryonic kidney cells (HEKs—often various clones), HeLa cells, SH-SY5Y cells, Neuro-2a, and NSC-34, however, other cell lines are used too. NSC-34 cells are a hybrid cell line generated from a fusion of murine neuroblastoma cells and murine embryonic spinal cord enriched in motor neurons (Cashman et al., 1992), making them a particularly useful immortalized model for studying ALS/FTLD.

Chosen cell lines are often genetically manipulated to express mutant forms of ALS/FTLD-associated proteins *via* transient transfection methods. This has several advantages over examining endogenous proteins as it allows for the delivery of mutant genes, fusion constructs, and overexpression. A common strategy involves the use of fluorescently tagged SOD1 mutants (Turner et al., 2005; Prudencio et al., 2009; Stevens et al., 2010; Münch et al., 2011; Polling et al., 2014; Farrawell et al., 2015, 2019; McAlary et al., 2016; Ayers et al., 2017; Pokrishevsky et al., 2017, 2018; Zhong et al., 2017; Crosby et al., 2018), TDP-43 mutants and fragments (Zhang et al., 2009; Yang et al., 2010; Walker et al., 2013; Farrawell et al., 2015; Zeineddine et al., 2017; Chen and Cohen, 2019; Chen et al., 2019; Sackmann et al., 2020), and FUS (Mastrocola et al., 2013; Britton et al., 2014; Yang et al., 2014; Farrawell et al., 2015; Patel et al., 2015; Bogaert et al., 2018; Maharana et al., 2018). Genetically encoded fluorophore tags, such as green fluorescent protein (GFP), are a highly useful method to specifically interrogate a protein of interest in cells (Day and Davidson, 2009), and have formed an important part of understanding the dynamics of ALS/FTLD mutant proteins in the physiologic environment.

At least for SOD1, there is good evidence to suggest that the fusion of GFP to it’s C-terminus does not significantly augment its ability to fold or dimerize (Stevens et al., 2010). Folding or dimerization of tagged TDP-43 or FUS has not been assessed; however, fluorescent protein-tagged versions of either of these proteins appear to localize correctly in cells, suggesting that tagging is not overly detrimental. Some researchers use smaller non-fluorescent affinity tags to overcome non-physiological interactions induced by fluorescent protein tags. Another advantage associated with fluorescent protein tagging of aggregation-prone proteins is that the localization of proteins into inclusions can sometimes mask epitopes detected by commercial antibodies (Prudencio and Borchelt, 2011). Since the fluorescent protein itself is incorporated into protein inclusions when tagged to aggregation-prone proteins, it is much easier to ascertain the presence of inclusions in cell populations using this system. One can then effectively utilize fluorescence microscopy or flow cytometry to detect the presence of fluorescent protein inclusions within cells (Ramdzan et al., 2012), or from cell extracts (Whiten et al., 2016; Zeineddine et al., 2017).

An advantage of fluorescent tagging of aggregation-prone proteins in cells is the capability to perform biophysical analyses by exploiting the photophysical properties of the fluorophore itself, such as fluorescence recovery after photobleaching (FRAP; Axelrod et al., 1976) or fluorescence loss in photobleaching (Lippincott-Schwartz et al., 2003). A major disadvantage of immunodetection using either affinity epitopes or endogenous epitopes is that this requires the fixation of cells, making it challenging or impossible to analyze dynamic events. Combined usage of both fluorescent tagging and FRAP has revealed that specific aggregation-prone proteins can localize to distinct inclusion types (Kaganovich et al., 2008) and that the mobility of SOD1, TDP-43, and FUS in and outside inclusions and other compartments can be examined using photobleaching (Matsumoto et al., 2005, 2006; Weisberg et al., 2012; Farrawell et al., 2015, 2018; Park et al., 2017).

Key findings from the usage of fluorescently tagged SOD1 in immortalized cell lines related to protein homeostasis include that: (1) disease-associated mutant forms of SOD1 are significantly more aggregation-prone than wild-type SOD1 (Prudencio et al., 2009; Prudencio and Borchelt, 2011; McAlary et al., 2016; Ayers et al., 2017; Crosby et al., 2018; Pokrishevsky et al., 2018; Farrawell et al., 2019; Crown et al., 2020); (2) SOD1 mutants are a target of ubiquitin proteasome degradation and partition exclusively to the juxtanuclear quality control compartment that triages proteins for proteasomal degradation (Matsumoto et al., 2005, 2006; Weisberg et al., 2012; Farrawell et al., 2015; Ayers et al., 2017; Park et al., 2017); and (3) the aggregation and associated toxicity of SOD1 mutants can be alleviated *via* overexpression of components of the cellular quality control machinery or small molecules (Walker et al., 2010; Xia et al., 2011; Park et al., 2017; Pokrishevsky et al., 2017, 2018; Farrawell et al., 2019). For TDP-43 and FUS, fluorescent tagging has been similarly successful, especially when examining the dynamics of FUS and TDP-43 biomolecular condensates (Patel et al., 2015; Maharana et al., 2018; Qamar et al., 2018; Gasset-Rosa et al., 2019; Mann et al., 2019). Considering the growing understanding of the number of diverse and transient structures formed in cells (Nedelsky and Taylor, 2019), experiments investigating protein aggregation in cells must be carefully interpreted when examining fixed samples. What is thought to be an aggregate may simply be a transient structure in some cases.

**Figure 3:**
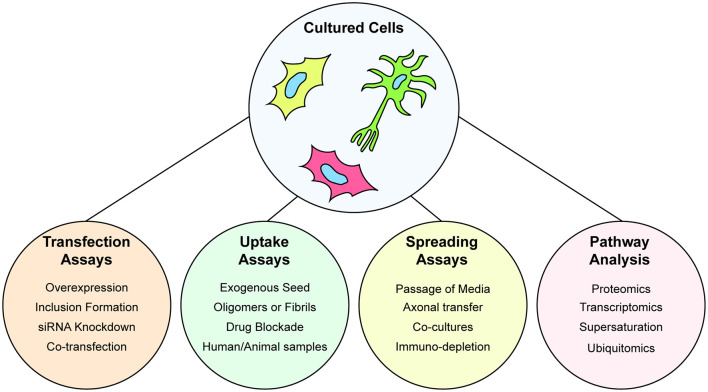
Understanding protein aggregation and prion-like spread using cultured cells. Cultured cells are amenable to genetic manipulation *via* transient or stable transfection, or gene editing to express fluorescently tagged wild-type or mutant forms of proteins. Cultured cells are used to study the ability of cells to uptake preformed protein aggregates in the form of purified aggregates, aggregates from other cultures, or extracts from organisms. Spreading of prion-like particles can also be assayed by serial passage of conditioned media, axonal spread through the use of microfluidic co-cultures. Furthermore, cultured cells are amenable to multiple types of omics that can provide information on wide-scale alterations to the proteome or transcriptome.

Immortalized cell lines are a highly useful model to examine the prion-like propagation of protein misfolding and aggregation in cells, particularly when paired with purified protein samples. Indeed, a combination of transiently expressed GFP-tagged SOD1 variants, and *in vitro* aggregated purified SOD1 protein was utilized to determine the prion-like properties of SOD1 aggregates (Münch et al., 2011). The ability to use exogenous aggregates that can be pure amyloidogenic peptides from a protein, or have the amyloidogenic region(s) deleted, is a powerful assay for understanding the structure-function relationship of prion-like proteins. As such, this system has been used to examine the seeding of GFP-tagged TDP-43 by various exogenously added TDP-43 peptide-derived amyloid fibrils (Shimonaka et al., 2016) or fibrils composed of full-length TDP-43 (Furukawa et al., 2011). The power of assays that utilize transgene expression of proteins in cultured cells is exemplified by the ability to seed aggregation of the reporter proteins *via* the addition of samples from human or animal *in vivo* sources (Nonaka et al., 2013; Pokrishevsky et al., 2017; Porta et al., 2018; Laferrière et al., 2019). Furthermore, the capability of prion-like proteins to replicate and infect can be easily examined in cells by the simple passage of the conditioned media to new naive cultures as has been shown for prion-like SOD1 conformations (Grad et al., 2014). This spread of protein aggregates between cultured cells can be examined by using dual fluorescent reporter, or split reporter, strategies. In these assays, two separate populations of cells are respectively transfected with prion-like proteins with different color fluorescent tags or split fluorescent/luminescence tags. Mixing of the separate cell populations and subsequent co-culturing allows for protein transfer between the populations which can be detected using fluorescence microscopy, flow cytometry, or a plate reader (Feiler et al., 2015; Zeineddine et al., 2017; Sackmann et al., 2020). For a visual summary of the assays that can be performed with cultured cells, see Figure 3.

To complement tractable and scalable cell-based experiments, animal experiments are required to examine the effect of prion-like protein aggregation on physiology and disease pathogenesis.

## Invertebrate Models of ALS

The genetic power and experimental tractability of small animal models (worm, fly, and fish) provide unique opportunities to investigate the pathological role of ALS-associated protein aggregates. Here we will discuss models for neurodegeneration using *Caenorhabditis elegans* and *Drosophila melanogaster*, focusing on insights gained on pathological mechanisms of SOD1, TDP-43, and FUS concerning proteostasis mechanisms and prion-like propagation.

### Caenorhabditis elegans

The nematode (roundworm) *C. elegans* has been used for decades in the study of neuronal development (Sulston et al., 1983), anatomy (White et al., 1986), and cellular/molecular function (Hobert, 2013; Walker et al., 2019). This extensive study has led to *C. elegans* becoming one of the most well-characterized experimental nervous systems, being the only adult animal for which the synaptic connectivity of all its ~300 neurons is known (Cook et al., 2019). More recently, the worm model has become increasingly popular for studies of neurodegeneration and pathology, in part due to its short lifespan (2–3 weeks), genetic tractability, transparent nature, amenability to high-throughput genetic or pharmacological screens (Kaletta and Hengartner, 2006), and the fact that >80% of the *C. elegans* proteome has human homologs (Lai et al., 2000). In particular, the genetic accessibility of *C. elegans* permits the rapid generation of transgenic lines (within 1–2 weeks) with expression restricted to specific cells or tissues, including the remarkable ability to express transgenes in individual neurons. Also, the transparent nature of *C. elegans* enables imaging of fluorescence-tagged proteins in specific tissues/cells within a whole organism (Heppert et al., 2016), which has been invaluable in making mechanistic links between protein aggregation and behavioral deficits. Moreover, the short lifespan of *C. elegans* facilitates aging experiments to be performed rapidly and cost-effectively.

In this section, we will focus on studies that use *C. elegans* transgenic lines to investigate the mechanisms through which ALS-associated mutations in SOD1, TDP-43, and FUS contribute to protein aggregation, neurodegeneration, and behavioral defects concerning proteostasis mechanisms and prion-like propagation. Other aspects of ALS (and other neurodegenerative disease) pathology are covered in detail in reviews elsewhere (Dimitriadi and Hart, 2010; Li and Le, 2013; Therrien and Parker, 2014).

#### Superoxide Dismutase-1

Most studies of SOD1 using *C. elegans* involve the transgenic expression of human wild-type (WT) or mutant SOD1 (hSOD1) tagged with fluorescent proteins to facilitate visualization of protein localization (i.e., whether diffuse in the cell or aggregated). These studies have shown that expression of disease-associated mutant forms of SOD1 (usually G85R, G93A) is associated with increased aggregation and behavioral deficits. The formation of aggregates during aging is correlated with defects in the proteostasis network (Hipp et al., 2019). For example, expression of hSOD1-G85R in all neurons resulted in locomotor deficits and inclusion-like structures of hSOD1-G85R-YFP (yellow fluorescent protein) in the cell bodies of motor neurons along the ventral nerve cord, observed *via* fluorescence microscopy and electron microscopy (Wang et al., 2009a). These observations correlated with reduced numbers, fluorescence intensity, and motility of pre-synaptic markers such as GFP-tagged synaptobrevin. Interestingly, despite hSOD1-YFP fluorescence being observed in most neurons, some individual neurons did not display aggregates at the fourth larval stage (the last larval stage before the adult moult; Wang et al., 2009a), although later time points were not analyzed in this study. This indicates that there are neuron-subtype-specific differences in how misfolding-prone SOD1 is sequestered in individual cells. Interestingly, expression of human SOD1 (hSOD1) mutants G85R, G93A, and G127X in *C. elegans* muscle showed limited toxic effects (after assaying motility, myofilament organization, muscle development, and egg-laying) despite all hSOD1(mutant) lines showing clear aggregate formation (Gidalevitz et al., 2009).

Wang et al. (2009a) used their hSOD1 *C. elegans* model to perform a genome-wide RNAi screen for genes that when knocked down led to an increase in fluorescence aggregates; these genes were therefore postulated to normally suppress or delay aggregation formation. Eighty-one hits were identified, of which 27.2% were factors involved in proteostasis (protein chaperones, misfolding, and turnover) including heat shock factor 1 (HSF1), Hsp110, a DnaJ family member A2 (DNJ-19), an Hsp70 family member (STC-1), E3 ubiquitin ligase components SEL-10 and RBX-1, and proteins involved in SUMOylation [small ubiquitin-like modifiers (SUMOs)] UBC-9 and GEI-17. Several of these hits were validated through genetic crosses (see Wang et al., 2009a for details).

Later studies examining the effects of transgenic hSOD1-G93A expression in GABAergic motor neurons of the ventral nerve cord (using the *unc-25* promoter) confirmed earlier findings, showing that expression of GFP-tagged hSOD1-G93A is correlated with reduced locomotion and the formation of large aggregates (Li et al., 2013, 2014). Interestingly, critical defects were observed to a similar extent in hSOD1-WT and hSOD1-G93A transgenic lines, such as age-dependent locomotor dysfunction and motor neuron degeneration; hSOD1-WT also formed aggregates in motor neurons—albeit to a lesser extent than hSOD1-G93A (Li et al., 2014). As toxicity associated with hSOD1^WT^ expression was not observed in the hSOD1 pan-neuronal lines (Wang et al., 2009a), these authors suggested that motor neurons may be more susceptible to hSOD1-linked toxicity, or that their transgene expression levels were simply higher compared with pan-neuronal lines. In both hSOD1^WT^ and hSOD1-G93A lines, cell-autonomous over-expression of key autophagy initiator UNC-51 partially alleviated axon guidance defects observed with hSOD1 expression (Li et al., 2014). UNC-51 is required for proper localization of the Netrin receptor UNC-5, a critical player in axon guidance (Ogura and Goshima, 2006), which may explain its involvement in this phenotype—although a role specifically in autophagy has not yet been explored.

Conventional microinjection protocols for generating transgenic *C. elegans* result in the formation of multi-copy transgene arrays (Mello et al., 1991), which express at high levels. Over-expression of hSOD1 has been linked to deleterious effects even for wild-type hSOD1 in *C. elegans* (Li et al., 2014). This makes it challenging to differentiate the effects of protein overexpression from that of ALS mutations, or to identify interventions that address one or the other. To overcome this issue, Baskoylu et al. (2018) generated single-copy insertions of *C. elegans* wild-type SOD-1 and A4V, H71Y, and G85R mutants (by comparison of *C. elegans* and human SOD1 sequences) *via* Mos1-mediated single copy insertion (MosSCI). Toxic effects were more subtle in MosSCI transgenic lines compared with conventional multi-copy arrays expressing hSOD1 (Baskoylu et al., 2018). One of the most pronounced effects was the formation of hSOD1^WT^-YFP inclusions when this transgene was co-expressed in the same neurons expressing single-copy insertions of mutant *C. elegans* SOD-1 (ceSOD1): single-copy insertions of mutant ceSOD1 showed significantly higher hSOD1^WT^-YFP inclusions compared with ceSOD1^WT^ or the empty vector control. The authors theorize that the hSOD1^WT^-YFP inclusions were possibly seeded by misfolded mutant *C. elegans* SOD1 protein in MosSCI models, suggesting a prion-like propagation effect (Baskoylu et al., 2018).

Mislocalization of SOD1 to the nucleus is linked to toxicity (Gertz et al., 2012). Mutations introduced in SOD1 to disrupt the consensus Nuclear Export Signal (NES) showed nuclear localization of GFP/YFP-tagged SOD1 in cultured cells and *C. elegans* neurons (Gertz et al., 2012). Although the NES-disrupting mutation L42Q by itself did not cause severe lifespan defects, in combination with the G85R mutation, expression of hSOD1^L42Q/G85R^ in *C. elegans* neurons led to significantly reduced lifespan compared with hSOD1^WT^ controls (Zhong et al., 2017).

#### Transactive Response DNA-Binding Protein 43

*C. elegans* is an excellent model system to assess prion-like spreading due to its well-defined, stereotyped anatomy and transparent nature, enabling the visualization of fluorescently tagged proteins across multiple tissues in a living organism (Nussbaum-Krammer and Morimoto, 2014). Like for SOD1, numerous studies have used overexpression of wild-type or ALS-linked variants of human TDP-43 (hTDP-43) to model aspects of pathology in *C. elegans*. Pan-neuronal expression of YFP-tagged hTDP-43(WT), ALS-associated mutant forms (Q331K and M337V), or the C-terminal 25 kDa fragment (hTDP-C25, residues 219–414) led to severe locomotor defects (Zhang et al., 2011). Analogous to human pathological data (Arai et al., 2006; Neumann et al., 2006), hTDP-C25 was found predominantly in the insoluble fraction, suggesting a high propensity for aggregation. Since an elevated temperature was linked to increased accumulation of hTDP-C25-YFP in both soluble and insoluble fractions, Zhang et al., 2011 used RNAi to knockdown components of the protein quality control network, finding that HSF1 was a major protective factor in toxicity in hTDP-43 protein aggregation. *HSF-1* partial loss-of-function mutations enhanced the locomotor defects observed in hTDP-C25-YFP transgenic lines. Besides, the insulin-like/IGF1 signaling (IIS) receptor DAF-2, which requires HSF1 for pro-longevity effects (Morley and Morimoto, 2004), also modulates the toxicity of hTDP43. *daf-2* mutants live twice as long as WT animals (Kenyon et al., 1993), and genetic crosses between *daf-2(-)* and hTDP-43 transgenic lines improved locomotion and reduced hTDP-43-YFP aggregation (Zhang et al., 2011). These data suggest that molecular chaperones and protein quality control play an important role in attenuating hTDP43 aggregation and related behavioral deficits.

Neuronal expression of untagged wild-type and mutant (G290A, A315T, and M337V) hTDP-43 also showed similar locomotion defects, with some evidence of aggregate formation in the nucleus of *C. elegans* neurons (assayed *via* immunostaining) in all transgenic lines (Liachko et al., 2010). Although both WT and mutant hTDP-43 lines showed reduced locomotion on day one of the assays, this was more severe in ALS-linked mutant lines and deteriorated more rapidly in hTDP-43(mutant) lines with increasing age. hTDP-43(mutant) lines also showed degeneration of GABAergic motor neurons, whereas hTDP-43^WT^ animals did not show significant differences compared to non-transgenic controls (Liachko et al., 2010). The authors also show that phosphorylation at S409/410 is important for toxicity in *C. elegans* models, as mutating the S409/410 sites to alanine in hTDP-43(mutant) lines was able to partially rescue the locomotion defects observed in G290A and M337V transgenic animals (Liachko et al., 2010). As TDP-43 phosphorylated at S409/410 is linked to pathological inclusions in both sporadic and fALS, as well as FTLD (Inukai et al., 2008; Neumann et al., 2009), this demonstrates that the *C. elegans* models generated in these studies recapitulate aspects of TDP-43 pathology observed in humans.

Restricting expression of wild-type and mutant (A315T) hTDP-43 to GABAergic motor neurons of *C. elegans* led to adult-onset, age-dependent paralysis and progressive motor neuron degeneration in hTDP-43(mutant) lines (Vaccaro et al., 2012b). This is distinct from the models described earlier, which already showed significant locomotor defects on the first day of adulthood (Liachko et al., 2010; Zhang et al., 2011), suggesting that motor neuron transgenic lines are not affected developmentally nor show general deficiencies. Nonetheless, motor neuron-restricted transgenic lines showed hTDP-43^A315T^ aggregate formation in the cytoplasm and nucleus of neurons in day 1 adults. The authors suggest that their model, with its strong age-dependent phenotypes, can be used for medium-throughput screens for genetic modifiers or small molecule inhibitors of hTDP-43 toxicity (Vaccaro et al., 2012b). Indeed, the compound TRVA242 was found to potently rescue locomotor defects of this *C. elegans* model in a screen of 3,765 novel small molecule derivatives of pimozide (Bose et al., 2019), a neuroleptic proposed to be used as a therapeutic for ALS (Patten et al., 2017). In addition to Methylene Blue, a compound that enhances oxidative stress responses (Stack et al., 2014), compounds known to act specifically on endoplasmic reticulum (ER) stress [such as salubrinal (Boyce et al., 2005) and guanabenz (Tsaytler et al., 2011)] also protected against hTDP-43^A315T^-mediated locomotor deficits in the *C. elegans* model (Vaccaro et al., 2013). This result adds to growing evidence that ER stress may be an attractive therapeutic target for ALS (Medinas et al., 2017a).

In a study focusing on the *C. elegans* ortholog of TDP-43 (TDP-1, referred to here as ceTDP-43), the authors showed that ceTDP-43 knockout was linked to sensitivity to oxidative and osmotic stress, as well as increased lifespan at moderate (20°C) temperatures. Unlike effects on hTDP-43 transgenic animals, the effects of the IIS pathway on ceTDP-1-mediated stress responses and lifespan are less clear—two distinct DAF-2 insulin receptor mutants have opposing effects, making it more difficult to derive a conclusive mechanistic link. Nonetheless, ceTDP-43 does appear to be required for the stress resistance in DAF-2 mutants as well as age-dependent proteotoxicity (Vaccaro et al., 2012c).

#### Fused in Sarcoma

Pan-neuronal expression of wild-type and mutant human FUS (hFUS) in *C. elegans* leads to cytoplasmic mislocalization, aggregation, and locomotor defects (Murakami et al., 2012). Murakami et al. (2012) generated a series of GFP-tagged hFUS transgenic lines, including WT FUS, clinical mutations (R514G, R521G, R522G, R524S, and P525L), and C-terminal truncations (FUS513 and FUS501). The C-terminal truncations aim to resemble human C-terminal splicing/frame-shifting truncation mutations associated with severe ALS phenotypes (Dejesus-Hernandez et al., 2010; Belzil et al., 2011). GFP-tagged hFUS R522G, R524S, P525L, FUS513, and FUS501 showed clear mislocalization of hFUS in the cytoplasm of neurons in the head and body of *C. elegans*, whereas WT hFUS and R514G or R521G mutations showed primarily nuclear localization. Cytoplasmic mislocalization of FUS is correlated with behavioral deficits, with only the strains showing cytoplasmic FUS also displaying age-dependent locomotor defects. These strains also showed a reduced lifespan compared with controls, as well as colocalization of FUS-GFP variants with cytoplasmic stress granule markers following acute heat stress (Murakami et al., 2012). To test whether mutant FUS can, under stress conditions, trigger the mislocalization of FUS^WT^ from the nucleus to the cytoplasm, the authors co-expressed TagRFP-hFUS^WT^ together with GFP-FUS^P525L^. After heat shock, TagRFP-WT-hFUS remained in the nucleus whereas GFP-FUS^P525L^ could be observed in both the nucleus and cytoplasm (in cytoplasmic structures resembling stress granules). The authors conclude that mutated FUS is likely to be pathogenic through a gain-of-function effect, rather than through titrating WT-FUS from the nucleus. In future work, it would be interesting to assess whether the colocalization of FUS with stress granules can be initiated by stressors other than acute heat stress (Huelgas-Morales et al., 2016), such as oxidative stress (Vance et al., 2013) or in the context of aging (Cao et al., 2020). *C elegans* is an ideal model system to explore the biology of stress signaling during aging due to its short lifespan and ease of growth/maintenance (Olsen, 2006).

In a *C. elegans* model expressing wild type and S57Δ mutant hFUS in GABAergic motor neurons, hFUS transgenic lines showed adult-onset, age-dependent locomotion deficits (Vaccaro et al., 2012b). These age-dependent behavioral effects correlate with motor neuron degeneration, which is more severe in hFUS^S57Δ^ transgenic animals compared with hFUS^WT^ (Vaccaro et al., 2012b). Using the same model, the neuroprotective agent Methylene Blue was able to alleviate behavioral defects in hFUS^S57Δ^ animals in a dose-dependent manner, potentially by protecting against oxidative stress (Vaccaro et al., 2012a). Given the link between oxidative stress, aging, and proteostasis, further investigation into how oxidative stress responses modulate FUS pathology will be of significant interest.

### Drosophila melanogaster

Research using the fly *Drosophila melanogaster* has made significant contributions to our understanding of nervous system development, function, and disease (Bellen et al., 2010; Owald et al., 2015). *Drosophila* has been used as a model organism for over 100 years: its genetic power combined with a relatively small brain (~250,000 neurons in adult) and complex array of behaviors that can be rapidly assessed experimentally, provides a powerful model animal to pursue investigations into the mechanisms of neuronal dysfunction (Casci and Pandey, 2015). Like *C. elegans*, *Drosophila* is amenable to genetic manipulation *via* RNAi (Dietzl et al., 2007) as well as tissue-specific transgene expression *via* the commonly-used Gal4/UAS (Upstream Activation Sequence) system (Brand and Perrimon, 1993). *Drosophila* is also cheap to grow and maintain, has a short generation time (~10 days), and is a more practical and ethical system than mammalian models to perform large-scale genetic or pharmacological screens (St Johnston, 2002; Hales et al., 2015). Another key advantage of *Drosophila* models is the high degree of genetic conservation with the human genome (~60%; Dietzl et al., 2007). Last, sophisticated imaging approaches have been developed for systematic investigation of *Drosophila* neurons, both in terms of neuroanatomy (Jenett et al., 2012) as well as “functional imaging” of neuronal activity, i.e., calcium/voltage sensors and optogenetics (Simpson and Looger, 2018). Indeed, current advances in mapping the synaptic connectivity of the *Drosophila* nervous system are well underway, and early data releases are showing exciting promise for neurobiology research in this model animal (Zheng et al., 2018).

#### Superoxide Dismutase 1

Early fly models, like those in *C. elegans*, were based on the expression of human SOD1 in *Drosophila* neurons. Flies expressing wild-type hSOD1 as well as A4V and G85R mutant forms in motor neurons showed no gross developmental or lifespan defects but showed age-dependent progressive motor defects in all lines when compared with a control line expressing *Drosophila* SOD1 (dSOD1). Interestingly, although the hSOD1-G85R line showed the most significant locomotor defect, this did not correlate with a gross reduction in the number of motor neurons in the thoracic ganglia or with the formation of insoluble species in these neurons (Watson et al., 2008). Also, expression of mutant or wild-type hSOD1 led to upregulation of Hsp70 expression in surrounding glial cells—whether this glial response is protective to motor neurons is not known (Watson et al., 2008). Indeed the role of the heat shock response in glial cells in ALS pathogenesis is not yet clear and is an area of active study (Robinson et al., 2005; Liddelow et al., 2017; San Gil et al., 2017).

Ubiquitous expression of zinc-deficient hSOD1-D83S in *Drosophila* resulted in age-dependent locomotor defects but no substantial loss of neurons or reduction in lifespan compared with non-transgenic or hSOD1^WT^ transgenic flies (Bahadorani et al., 2013). Tissue-specific expression of hSOD1-D83S in all neurons or in glia had the most potent effect on locomotion, with motor neuron-restricted expression having a moderate impact. However, no defects were observed upon expression in muscle, and no reduction in lifespan was seen compared to hSOD1^WT^ transgenic animals. These defects correlated with increased sensitivity to the oxidative stressor paraquat and dysfunction of the mitochondrial respiratory chain (Bahadorani et al., 2013). These results agree with the previously mentioned purified protein and cell culture data, suggesting that structural destabilization of SOD1 is a crucial factor in the formation of toxic species and aggregates in cells.

dSOD1 is highly similar to hSOD1, being identical at 104/153 residues. Şahin et al. (2017) engineered the ALS-associated mutations G37R, H48R, H71Y, and G85R into the endogenous locus of dSOD1 to generate mutant “knock-in” lines. These lines showed varying effects: compared with dSOD1(WTLoxP) controls, homozygous dSOD1 H71Y showed reduced lifespan, reduced fertility, and increased sensitivity to oxidative stress. dSOD1-G85R and dSOD1 H48R lines were developmentally lethal as homozygotes, but dSOD1-G85R heterozygotes showed normal lifespan and oxidative stress responses. dSOD1-G37R animals were phenotypically normal compared to controls. In larvae, dSOD1-G85R and dSOD1-H71Y homozygotes showed severe climbing defects that were suppressed in heterozygotes of each transgene. These were not correlated with a gross reduction in motor neuron number. However, dSOD1-G85R and dSOD1-H71Y animals showed reduced dSOD1 protein levels and enzymatic activity (Şahin et al., 2017). These models, which express ALS-linked mutant SOD1 from the endogenous dSOD1 locus, will be useful tools for future analysis or high-throughput genetic/pharmacological screens.

#### Transactive Response DNA-Binding Protein 43

Expression of a repeated form of amino acids 342–366 (12xQ/N) of TDP-43 in the *Drosophila* eye leads to the formation of insoluble aggregates that were by themselves not strongly neurotoxic (Cragnaz et al., 2014). Over-expression of the *Drosophila* ortholog of TDP-43 (TBPH, referred to here as dTDP-43) in the eyes leads to significant levels of tissue degeneration. This toxicity was completely reverted by the co-expression of EGFP-tagged 12xQ/N. The authors proposed that dTDP-43 may be sequestered in aggregates formed by EGFP-12xQ/N, thereby titrating excess dTDP-43 (Cragnaz et al., 2014). In this case, the formation of aggregates is protective. When EGFP-12xQ/N is expressed in fly neurons, it results in an age-associated locomotor defect and a shortened lifespan (Cragnaz et al., 2015). In the neuronal model, the authors suggest that the locomotor defect is correlated with reduced levels of endogenous dTDP-43 (protein and mRNA levels), due to dTDP-43 being sequestered in EGFP-12xQ/N aggregates (Cragnaz et al., 2015). This can be compensated in younger animals by increased protein production, but in older animals, the lower capacity for protein synthesis (Liang et al., 2014; Anisimova et al., 2018) may lead to a loss-of-function effect due to reduced dTDP-43 levels.

Over-expression models where wild-type or human hTDP-43 is expressed in fly neurons have also provided interesting insights into TDP-43-linked ALS/FTLD pathology. Miguel et al. (2011) generated *Drosophila* models expressing FLAG-tagged hTDP-43^WT^, hTDP-43 with a defective NLS (hTDP-43^ΔNLS^—predominantly cytoplasmic localization) or hTDP-43 with a defective NES (hTDP-43^ΔNES^—restricted to nuclear localization). Neuron-specific expression of these transgenes was larval lethal, so an inducible system was used to bypass developmental effects (Miguel et al., 2011). Compared with controls, all inducible transgenic flies showed a reduced lifespan. Biochemical analysis (immunoblot) demonstrated that hTDP-43, hTDP-43^ΔNES^, and hTDP-43^ΔNLS^ proteins could be detected in monomeric form or as high molecular weight (HMW, potentially aggregated) species, although hTDP-43^ΔNES^ and hTDP-43^ΔNLS^ expression showed more HMW species compared with hTDP-43^WT^. When probed with an antibody against phospho-TDP-43(S409/410), hTDP-43^ΔNES^ HMW species, but not those of hTDP-43^WT^ or hTDP-43^ΔNLS^, were strongly labeled by the pTDP-43 antibody. Interestingly, immunostaining did not reveal the presence of inclusions formed by the expression of any of these transgenes, suggesting that toxicity (reduced survival) does not require the formation of inclusions. This study also demonstrated cell-type-specific toxicity resulting from the expression of hTDP-43 transgenes: in adult neurons, both nuclear and cytoplasmic accumulations of TDP-43 result in toxicity, whereas in muscle and glial cells, only the accumulation of cytoplasmic species of TDP-43 was toxic (Miguel et al., 2011). This could be due to differences in proteostasis mechanisms in different tissues, such as the capacity for protein production, differences in post-translational modification, or sequestration of aggregated/oligomeric forms (Vilchez et al., 2014).

TDP-43 autoregulates its own levels through binding to a TDP-43 binding region (TDPBR) in its 3′ UTR (Ayala et al., 2011). Expression of hTDP-43 containing the TDPBR in *Drosophila* retinal cells led to significantly reduced protein and mRNA levels for *hTDP-43* (Pons et al., 2017), similar to observations in cultured cells and mammalian models (Ayala et al., 2011; Polymenidou et al., 2011). This *hTDP43_TDPBR* transgenic line can then be used to perform genetic crosses with *Drosophila* mutant strains to identify other factors that modulate *hTDP-43* levels through the TDPBR. Pons et al. (2017) used this method to identify *Drosophila* splicing factors that modulated *hTDP-43* levels, and in a follow-up study, identified the protein CG42724 [and its human orthologue Transcription elongation regulator 1 (TCERG1)] as a modulator of *hTDP-43* protein levels (Pons et al., 2018).

TDP-43 has been shown to associate with human Hsc70 in cultured mammalian cells (Freibaum et al., 2010). In *Drosophila*, one of the Hsc70 proteins (Hsc70–4) is highly expressed in neurons and functions in synaptic vesicle exo- and endocytosis (Bronk et al., 2001; Chang et al., 2002). Coyne et al. (2017) showed that *Drosophila* Hsc70–4 associates with transgenic hTDP-43 in fly motor neurons. As a result, over-expression of hTDP-43 in motor neurons sequesters Hsc70–4, leading to reduced synaptic expression. This ultimately inhibits synaptic vesicle endocytosis and results in defects in synaptic transmission (Coyne et al., 2017). Interestingly, expression of ALS-linked mutant forms of hTDP-43 leads to locomotor defects that can be rescued by over-expression of wild-type Hsc70–4, but not mutant forms that are defective in chaperone (Hsc70–4^D10N^) or microautophagy functions (Hsc70–4^3KA^). This suggests that hTDP-43 compromises both chaperone and microautophagy activities of Hsc70–4 (Coyne et al., 2017).

#### Fused in Sarcoma

*Drosophila* models have also been useful in investigating the pathological roles of ALS-associated protein FUS. Transgenic *Drosophila* lines were generated expressing hFUS^WT^ and two disease-related variants hFUS^P525L^ and hFUS^R495X^ (Jäckel et al., 2015). Expression in motor neurons resulted in viability defects and locomotor defects in hFUS^WT^ and hFUS^P525L^, but not in hFUS^R495X^-expressing flies. Immunostaining showed that hFUS^WT^ localized to the nucleus, whereas hFUS^P525L^ showed an additional cytoplasmic staining, and hFUS^R495X^ was observed at relatively equal levels in the nucleus and cytoplasm. The lack of phenotype observed in hFUS^R495X^ flies is surprising because the R495X mutation is linked to a highly aggressive form of ALS (Chiò et al., 2009b). The pathological role of hFUS^R495X^ in this *Drosophila* model could not be associated with its partial cytoplasmic mislocalization, which is thought to be a critical disease mechanism (Kim and Taylor, 2017), suggesting that disease-modifying factors are missing in this model.

FUS expression can autoregulate its levels in cultured cells (Zhou et al., 2013; Dini Modigliani et al., 2014). Similarly, in *Drosophila*, over-expression of hFUS in fly motor neurons leads to decreased levels of the endogenous Caz protein (the *Drosophila* ortholog of FUS—dFUS; Machamer et al., 2014; Jäckel et al., 2015). These changes are correlated with alterations in neuromuscular junction structure and defects in synaptic transmission. In contrast, Caz/dFUS null alleles show enhanced synaptic transmission as demonstrated by increased excitatory junction potential (EJP) amplitude (Machamer et al., 2014). Therefore, although hFUS over-expression (both WT and mutant) leads to lower levels of Caz/dFUS, this is likely not the mechanism by which hFUS triggers synaptic dysfunction.

Both TDP-43 and FUS are components of the biomolecular condensate compartment known as stress granules through interactions in their respective LCDs (Harrison and Shorter, 2017). A *Drosophila* study performed a systematic domain deletion screen of FUS, to identify toxicity-mediating domains (Bogaert et al., 2018). Deletion of the N-terminal QGSY prion-like LCD partially rescued toxicity. The C-terminal RGG-2 region was also essential for toxicity, and the two domains appear to act synergistically. Interestingly, the prion-like QGSY domain is not found in Caz/dFUS, which may differentiate between toxicity caused by human and fly orthologs (Bogaert et al., 2018). The nuclear-import receptor Kap-β2 (Karyopherin-β2) inhibits phase-transition and fibrillization of FUS *in vitro*. Also, transgenic expression of Kap-β2 and could rescue the shortened lifespan of *Drosophila* expressing hFUS^R521H^, thereby mitigating hFUS induced motor-neuron toxicity *in vivo* (Guo et al., 2018).

**Figure 4:**
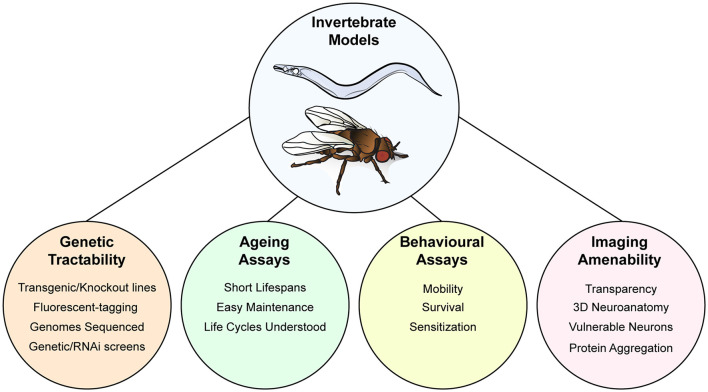
Invertebrate models of protein aggregation disorders. Invertebrate models are cheap and efficient systems that are highly amenable to genetic manipulation through the delivery of transgenes in the forms of human genes or fluorescent proteins, as well as knockout of endogenous genes. The short lifespan of these models makes them a powerful tool for assaying development and aging. Behavioral analysis can be performed to determine the effect of protein aggregation in these models, linking observed pathology to clinical phenotypes. Last, these models are highly accessible to imaging and provide access to well-understood neuroanatomy to assay prion-like spread.

For a visual summary of the advantages of using invertebrate models, see Figure 4.

### Mouse Models of ALS

Mouse models of ALS have been a key system by which our understanding of the underlying molecular pathology of ALS develops. In this section, we will describe the current mouse models used to examine both protein homeostasis and prion-like propagation in ALS.

#### Superoxide Dismutase-1

Since the seminal discovery identifying the association of SOD1 mutations with fALS (Rosen et al., 1993), several SOD1 transgenic mouse models have been generated. Despite the discovery of several other genes and a relatively small percentage of patients exhibiting SOD1 mutations, SOD1 mouse models are still the most extensively used model to investigate the pathogenesis and treatment of ALS. The first transgenic SOD1 mouse model expressed hSOD1 with the G93A mutation (denoted SOD1-G93A; Gurney et al., 1994). Transgenic SOD1-G93A mice exhibit phenotypic symptoms at 3–4 months of age and subsequently develop ubiquitinated SOD1 deposits, similar to those found in fALS post-mortem tissue (Bruijn et al., 1997). Years after its initial generation, this model provided the first *in vivo* clues indicating that SOD1 acts in a prion-like fashion. Deng et al. (2006) observed that crossing human wild-type SOD1 mice with SOD1-G93A transgenic mice accelerated ALS-like phenotypes. Furthermore, soluble human wild-type SOD1 was converted to an aggregated and detergent-insoluble conformation. Also, spinal cord homogenates from SOD1-G93A mice were used to seed recombinant purified SOD1 to form amyloid fibrils (Chia et al., 2010).

The studies described above hinted at the ability of SOD1 to propagate in a prion-like manner but lacked animal-to-animal transmission. Utilizing experimental paradigms from the prion field, Ayers et al. (2014) were the first to provide *bona fide* *in vivo* evidence of SOD1 prion-like propagation. Heterozygous mice expressing SOD1-G85R mutant C-terminally tagged with a yellow fluorescent protein (SOD1-G85R:YFP) do not develop an ALS phenotype (Wang et al., 2009b). However, when spinal cord homogenates from paralyzed SOD1-G93A mice were injected into the spinal cord of SOD1-G85R:YFP mice at PN0 (postnatal day 0), some (~50%) of these mice subsequently developed ALS-like phenotypes, including paralysis as early as 6 months of age, as well as reduced life-span (Ayers et al., 2014). However, when spinal cord homogenates from SOD1-G93A inoculated SOD1-G85R:YFP mice were subsequently re-passaged into newborn SOD1-G85R:YFP mice, all of these mice developed ALS symptoms, and at an earlier time-point. Furthermore, SOD1-G93A recipient mice exhibited SOD1-G85R:YFP protein aggregates in the spinal cord as well as in the brain stem, suggesting prion-like spread distal to the site of injection. Also, second passage inoculation of naïve adult SOD1-G85R:YFP mice resulted in paralysis of the ipsilateral hind-limb of the injection site within 1 month, followed by contralateral leg paralysis after 2 months and reaching end-stage paralysis within 3 months post-injection. Furthermore, post-mortem analysis of mice at several time points post-injection showed inclusion pathology in both neurons and astrocytes in the dorsal root ganglion, cervical spinal cord, and brain stem (Ayers et al., 2016), suggesting focal spreading throughout the CNS, a classic hallmark of pathological prion propagation. It is important to note that YFP-tagged SOD1 is not required as a template for seeding, as other work has shown untagged mutant SOD1 expressed in mice can be efficiently seeded by injection of preformed SOD1 aggregates (Bergh et al., 2015; Lang et al., 2015; Bidhendi et al., 2016; Bidhendi et al., 2018).

Whilst it is unclear how SOD1 spreads throughout the CNS, *in vitro* studies highlight the important role of exosomes in the extracellular transmission, yet *in vivo* experiments supporting such transmissibility have yet to be explored or reported. These studies may be of use to aid in the design of anti-propagation therapeutic avenues. In line with this, *in vitro* work has shown small molecules that interact with or near the Trp32 residue of SOD1 can reduce its aggregation propensity. Furthermore, Trp to Ser substitutions at the 32 position residue can suppress intra- and intercellular propagation (Grad et al., 2011, 2014), suggesting its involvement in SOD1 prion-like propagation. However, some research showed that recombinant SOD1 with a Trp32Ser (W32S) substitution produced a similar disease frequency and earlier disease onset compared to WT SOD1 when seeded in G85R-SOD1:YFP mice (Crown et al., 2020), suggesting that fibrillation through other amyloidogenic regions within SOD1 may also confer disease. The Trp32 residue is conserved to human SOD1 in beta-sheet 3 and not present within mice. To date, there are no reports of a transgenic SOD1 mouse model expressing modifications to the Trp32 residue, however, such a model may lead to further understanding its role in SOD1 aggregation and prion-like propagation.

Various prion isolates can develop a heterogeneous pathology giving rise to prion strains that are determined by variability within their tertiary conformation. Similarly, fALS SOD1 mutations display a heterogeneous disease pathology, which has been proposed in part due to their tertiary structure and propensity to aggregate (Prudencio et al., 2009). Using binary epitope mapping with several anti-peptide antibodies covering the entire SOD1 protein, Bergh et al. (2015) used insoluble material obtained from wild-type and mutant (G85R, D90A, and G93A) SOD1 mice to show evidence of two distinct strains (referred to as A and B by the authors). Strain A aggregates were observed in all SOD1 mutant mice investigated, while strain B was only observed in D90A mice. *In vitro*, biochemical/physical examination of strain A and B showed variation in their aggregation structure, kinetics, and fragility, with strain B showing increased aggregation kinetics and fragility (Lang et al., 2015). Furthermore, injection of strain A and B in the lumbar spinal cord of adult SOD1^G85R^ mice showed both strains were capable of seeding and transmissibility, with the rostral spreading of aggregates, subsequently causing end-stage death ~100 days following injection (Bidhendi et al., 2016). Later, injection of spinal cord homogenate from a patient carrying the SOD1-G127X mutation into transgenic mice carrying the same mutation showed propagation of strain A into the mice, suggesting that this aggregate conformer may exist in humans (Bidhendi et al., 2018).

Whilst the evidence and modeling are still in the early days, these data collectively support the ability of SOD1 to undergo prion-like propagation. Future experiments using SOD1 mice with various amyloidogenic regions within SOD1 deleted and/or the use of synthetic peptide samples composed of a single amyloidogenic segment of SOD1 would be useful to reveal the mechanistic details of this pathology.

#### Transactive Response DNA-Binding Protein 43

TDP-43 has been extensively implicated in ALS, in addition to FTLD, due to the high majority of both fALS and sALS patients exhibiting insoluble and ubiquitinated TDP-43 cytoplasmic inclusions within motor neurons and glia (Neumann et al., 2006). Several TDP-43 transgenic mouse models have been generated which show motor deficits, neuronal loss, and mislocalized TDP-43 inclusions (Lee et al., 2012). However, overexpression of WT TDP-43 can cause similar pathology, making it difficult to distinguish the pathological effects of various mutations without adequate control over transgene expression. To overcome these issues, several groups have produced sophisticated knock-in models to control for transgene overexpression. These models include introducing Hemi- and homologous TDP-43 transgene mutations at G298S, Q331, and M337V (Fratta et al., 2018; White et al., 2018; Ebstein et al., 2019; Gordon et al., 2019). Interestingly, these mice homologous for these transgenes show motor deficits and in some cases, motor neuron loss in the absence of mislocalized TDP-43, suggesting TDP-43 mislocalization may not be the cause of motor neuron toxicity in ALS (Fratta et al., 2018; White et al., 2018; Ebstein et al., 2019; Gordon et al., 2019).

Observations from ALS and FTLD post-mortem tissue suggests that TDP-43 also exhibits prion-like propagation and spreading throughout neuroanatomical tracts. Until recently there has been a lack of *in vivo* investigation into the prion-like propagation of TDP-43 using animal models. Employing a cell-based screening system, Porta et al. (2018) were able to apply sarkosyl-insoluble frontal cortex homogenates from FTLD-TDP subjects to identify those with seeding capacity. Subsequently, samples that showed *in vitro* seeding capacity were injected into the brains (neocortex, hippocampus, and thalamus) of doxycycline-regulatable transgenic mice expressing a human TDP-43^Δ NLS^ in forebrain neurons (under a CAMKIIa promoter). Recipient mice displayed pTDP-43 inclusions within the ipsilateral injection site within 1-month post-injection, with relatively few inclusions observed on the contralateral side. Furthermore, spatiotemporal immunohistochemical analysis of mice injected with TDP-43 seeding material showed a transneuronal-like spreading pattern of TDP-43 pathology in the 1–9 months following injection. Unsurprisingly, a large majority of TDP-43 aggregates were observed within forebrain neuronal cells where the CAMKIIa promoter controls the expression of TDP-43^Δ NLS^. However, TDP-43 aggregates were also found within the subcortical regions including the nucleus accumbens and caudate-putamen, suggesting regional spreading.

Furthermore, similar to ALS/FTLD post-mortem samples, TDP-43 aggregates were also sparsely observed in oligodendrocytes and astrocytes, suggesting local cell-to-cell transmission that is not dependent on the subcellular mislocalization of TDP-43. Further supporting this, seeding was also observed in wild-type mice injected with FTLD-derived material, however, the capacity was less than NLS-mice. Furthermore, injection of FTLD material in mice overexpressing cytoplasmic TDP-43 C-terminal fragments did not show TDP-43 pathology, indicating a role for the N-terminal sequence in TDP-43 in prion-like seeding. While this study provides initial *in vivo* evidence for the prion-like characteristics of TDP-43, further research is required to identify the specific regions of TDP-43 that are important for seeding. As mentioned previously, full-length TDP-43 and its CTFs have multiple amyloidogenic regions (Guenther et al., 2018a,b). Assessing the contribution of each of these segments would be essential to determine the sequence region by which TDP-43 aggregates propagate. This information would allow for the design of therapies that may block templating or cell-to-cell transmission of TDP-43 prion-like particles.

#### Fused in Sarcoma

FUS knockout mouse models do not show evidence of motor impairments or ALS associated histopathology, suggesting that a FUS loss-of-function mechanism is not responsible for ALS pathology. However, several groups have developed human wild-type FUS (hFUS) overexpression mouse models under various promoters. Mitchell et al. (2013) were the first to produce a FUS overexpression model, whereby hFUS levels were ~1.9 times higher than wild-type mice. These mice develop paralysis at 10–12 weeks of age (Mitchell et al., 2013). Furthermore, overexpression of hFUS resulted in the cytoplasmic mislocalization of the ectopic protein in the brain and spinal cord. However, cell loss was only observed in the spinal cord, indicating that lower motor neurons may be prone to FUS overexpression.

FUS mutations are inherited in an autosomal dominant fashion, with FUS-R521C mutations commonly observed in FUS-associated fALS patients. Qiu et al. (2014) developed a transgenic FUS-R521C mouse model that exhibited aggressive motor function deficits and reduced life span. These transgenic mice showed alterations in the transcription and splicing of the growth factor BDNF, which is regulated by FUS. Spinal cord homogenates from FUS-R521C mice showed a high propensity to form complexes with FUS-WT, consistent with experiments in HEK293T cells co-expressing mutant- and FUS-WT. Together with the autosomal dominant inheritance of FUS mutations, these data support a gain of function property of mutant FUS, which may sequester FUS-WT from carrying out its physiological role.

Recently, Zhang et al. (2020) employed two-photon imaging in combination with a knock-in mouse model of FUS-R521C. They showed that mutant FUS was incorporated and potentiated stress granule formation following arsenite challenge. Expression of mutant FUS also caused the cytoplasmic mislocalization of mutant FUS and upregulation of ubiquitin, which appeared to be also co-localized. Also, neurons that showed an inability to clear or disassemble stress granules died within days following arsenite challenge, highlighting the role of stress granule formation in the pathogenesis of ALS.

**Figure 5:**
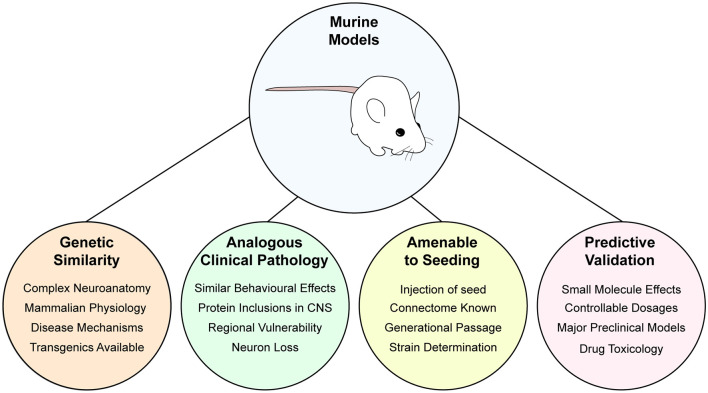
Murine models of protein aggregation and prion-like propagation. Murine models form the crux of our pathobiological understanding of the effects of protein aggregation and prion-like propagation in mammals. This is due to their genetic similarity with humans, which translates into similar physiology, similar disease susceptibility, and similar neuroanatomy. The input of disease-associated genes into mice often results in similar clinicopathological features including behavioral deficits, protein aggregation, and loss of neurons in specific tissues. Prion-like propagation can be assayed through the injection of seed protein aggregates into specific locations, and the passage of extracted prion-like seeds can be used to define strains. Finally, these models are the gateway to the clinic and are mostly used for the testing of drugs and therapies that are aimed at alleviating human disease.

To distinguish the pathological role of FUS aggregates without the confounding effects on RNA metabolism, Shelkovnikova et al., developed a transgenic mouse expressing a truncated human FUS protein (residues 1–359), which lacked the RNA recognition motif and NLS but still retained an N-terminal QGSY prion-like domain (Shelkovnikova et al., 2013). These mice developed paralysis, exhibited motor neuron loss, and reduced life-span. Furthermore, the truncated FUS protein formed aggregates, and subsequent FUS inclusions containing endogenous murine FUS were observed in the motor cortex and lower motor neurons. Collectively this study suggests that mutant FUS may exhibit a seeding capacity, which is capable of causing motor neuron degeneration in the absence of its role in RNA processing. For a visual summary of the key features of murine models, see Figure 5.

## Therapeutic Strategies to Target Proteostatic Impairment and Prion-Like Propagation in ALS

Currently, only two therapies exist for the treatment of ALS in humans. The first, riluzole, interrupts glutaminergic signaling (Bryson et al., 1996) potentially through preventing glutamate release (Kretschmer et al., 1998). Riluzole treatment led to increased motor function and survival in an initial clinical trial (Bensimon et al., 1994; Lacomblez et al., 1996); however, improvements in survival were not observed in a follow-up clinical study. Also, riluzole only extends lifespan by 3–6 months, and only in patients with moderate impairment (Bensimon et al., 1994; Lacomblez et al., 1996). The second compound, edaravone, is a free-radical scavenger (Yoshino and Kimura, 2006), which is primarily sold to promote recovery from stroke in Japan due to its ability to prevent edema, neuronal damage, and neurological defects in rats (Edaravone Acute Infarction Study Group, 2003). Edaravone is offered as a therapeutic to treat ALS in Japan, South Korea, the US, and Canada, but is not being used elsewhere (Yoshino, 2019). Clinical trials using edaravone did not demonstrate a benefit for the majority of patients, with a follow-up clinical study suggesting that edaravone only benefits patients with mild impairment [Yoshino and Kimura, 2006; Abe et al., 2014; Writing Group; Edaravone (MCI-186) ALS 19 Study Group, 2017]. The above highlights how the only two drugs currently offered to treat ALS symptoms are limited in scope, and there is a dire need for new therapies that potentially target one or more ALS-associated aggregating proteins.

### *In silico* and *In vitro* High-Throughput Identification of Potential Therapeutics

With improved computing power and rapidly-evolving methods to better examine protein structure, computational modeling is being utilized as a means for drug discovery. Due to the lack of need for biological material, and bioinformatic nature, computational screening of drugs can be carried out rapidly and efficiently. Additionally, high-throughput screens using *in vitro* models that recapitulate some aspects of ALS pathology can be utilized to rapidly screen potential drugs to determine their biological activity and therapeutic potential. A recent review of high-throughput screens investigating drugs that alter ALS pathology was published by McGown and Stopford (2018). This review describes studies that investigated reducing SOD1 transcription, protein aggregation, and oxidative stress as a therapeutic strategy, as well as the potential of anti-glutamatergic and cytoprotective drugs for the treatment of ALS. Studies focusing solely on drug candidates that modify protein aggregation that was described briefly in the review, and studies published since are discussed below.

#### Superoxide Dismutase-1

A computational screen found 16 novel compounds that bound mutant SOD1-A4V and prevented its aggregation *in vitro* (Ray et al., 2005), however, these compounds did not exert this effect in the presence of blood plasma (Nowak et al., 2010). Subsequent computational work identified 6 different novel compounds that bound SOD1 in the presence of plasma (Nowak et al., 2010), but these are yet to be tested *in vitro* or *in vivo*. Using *in vitro* aggregation assays of SOD1-G37R protein, Anzai et al. (2016) tested 640 FDA-approved drugs and identified seven hit compounds, with six able to completely suppress SOD1 aggregation (measured by turbidity). Three of these were statins (simvastatin, lovastatin, and mevastatin), another three were vitamin D derivatives (alfacalcidol, calcidiol, and calcitriol), and the final drug was miltefosine, an antimicrobial. A computational screen of ~4,400 compounds revealed that quercitrin, quercetin-3-β-D-glucoside, and epigallocatechin gallate (EGCG) bind directly to SOD1-A4V and inhibit ROS-induced SOD1 aggregation (Ip et al., 2017), but only the latter has been tested *in vitro* and *in vivo*. EGCG administered in SOD1-G93A mice improved disease compared to controls, delaying means symptom onset (128 days vs. 115 days), locomotor deficits measured by rotarod test (140 days vs. 120 days), and mortality (mean survival time 143 days vs. 123 days), however the mechanism was not described (Koh et al., 2006), and this drug has not progressed to clinical trials.

Using high-throughput *in vitro* screening using an inducible SOD1-G37R PC12 cell line, Broom et al. (2006) screened 116,680 compounds from various libraries and found 67 hit compounds. However, most compounds exhibited cytotoxicity or failed to have their effect replicated, and only 2 hits (no description available) are being investigated further. Rather than using SOD1 aggregation as a readout, Wright et al. (2012) instead investigated SOD1 transcription using PC12 cells transfected with SOD1-GFP to screen 30,000 compounds, with the most potent inhibitor of SOD1 transcription being *N*-[4-[4-(4-methyl benzoyl)-1-piperazinyl]phenyl]-2-thiophenecarboxamide. *In vivo* experiments using a SOD1-G93A mouse model showed that the drug was well tolerated, with toxicity estimated to be 75 mg/kg. These mice also showed a significant decrease in SOD1 protein levels, but aggregation was not described (Wright et al., 2012).

Finally, using iPSC-derived motor neurons from fALS patients, Imamura et al. (2017) screened 1,416 compounds and determined the Src/c-Abl tyrosine kinase inhibitor bosutinib improved iPSC-derived motor neuron survival, potentially through clearance of SOD1 aggregates by up-regulation of autophagy pathways. Intraperitoneal injection of bosutinib (5 mg/kg) into transgenic SOD1-G93A mice daily starting at 8 weeks of age for 5 weeks, reduced misfolded SOD1 and motor neuron degeneration in spinal cords, as well as delaying disease onset by 10.8 days and extending survival by 7.8 days (Imamura et al., 2017).

#### Transactive Response DNA-Binding Protein 43

Boyd et al. (2014) generated a doxycycline-inducible TDP-43-GFP expressing PC-12 cell line to screen ~75,000 small molecules to identify those that reduce sodium arsenite-induced TDP-43 aggregates as measured by microscopy. One compound, termed LDN-0130436, reduced TDP-43 aggregation in a concentration-dependent manner and was confirmed by immunoblotting. LDN-0130436 restored behavioral defects and protected against TDP-43-induced cell loss in *C. elegans* expressing mutant TDP-43^A315T^, however, effects on protein aggregation in this model were not reported (Boyd et al., 2014).

Using a luciferase-based fluorescence assay to measure TDP-43 inclusions in the murine Neuro-2a neuroblastoma cell line, Oberstadt et al. (2018) screened the LOPAC1280 library (Sigma–Aldrich) and found 16 active compounds that reduced TDP-43 dimerization by 30–82%. In an initial screen, riluzole, one of the two therapies offered to ALS patients, reduced N-terminal TDP-43 interactions by 82%, whilst chelerythrine and auranofin reduced interactions by 64% and 63%, respectively. During validation, 25 μM auranofin significantly reduced 98% of TDP-43 interactions, whilst 25 μM chelerythrine reduced 90% of TDP-43 interactions but exhibited a toxic effect at higher concentrations (Oberstadt et al., 2018). Fang et al. (2019) screened 3,350 compounds in HEK293 cells transfected with TDP-43 and 5,910 compounds in neural progenitor cells to identify drugs that affect stress granule formation. In this study, Anisomycin, mitoxantrone, and GSK2656157 significantly reduced stress granule formation, and interestingly mitoxantrone inhibited TDP-43^ΔNLS^ colocalization with stress granules (Fang et al., 2019).

Using patient-derived iPSCs differentiated into spinal motor neurons, Egawa et al. (2012) investigated the impact of four compounds on arsenite-induced TDP-43 inclusion formation and cell death. Only anacardic acid reduced TDP-43 mRNA expression, inclusion formation, and prevented cell death. Additionally, bosutinib (mentioned above for SOD1) demonstrated a similar reduction in TDP-43 inclusions in iPSC-derived motor neurons. Another study utilized a high-throughput image analysis method in iPSC-derived motor neurons from sALS patients to screen 1,757 bioactive compounds for the reduction of TDP-43 aggregation and expression levels (Burkhardt et al., 2013). This screen identified 38 hits that reduced TDP-43 aggregation, but not TDP-43 expression, and subsequent validation characterized four classes of compounds: cyclin-dependent, and c-Jun N-terminal kinase inhibitors, triptolide, and cardiac glycosides. Another study using iPSC-derived motor neurons from fALS patients expressing TDP-43 mutations screened 1,232 drugs using a high-throughput imaging system and identified the dopamine D2 receptor antagonist ropinirole as a potential therapeutic (Fujimori et al., 2018). Ropinirole prevented neurite regression, stress granule formation, TDP-43 aggregation, and prevented cell death of fALS-derived iPSCs.

Patten et al. (2017) screened 3,850 compounds in a transgenic *C. elegans* model expressing mutant TDP-43^A315T^ and identified several neuroleptics, which rescued paralysis. Further screening in a transgenic zebrafish model expressing mutant TDP-43^G348C^, led to the identification of pimozide as a potential therapeutic, which rescued paralysis, as well as motor neuron axon branching and neuro-muscular junction structures. The authors also describe that pimozide is already FDA-approved and the use of pimozide (1 mg daily) in a randomized double-blind clinical trial in Poland, led to a reduction in ALS severity (Szczudlik et al., 1998), but this is yet to be investigated elsewhere.

In a transgenic *Drosophila* model of ALS, in which motor neurons express mutant TDP-43^D169G^, TDP-43^G298S^, TDP-43^A315T^, or TDP-43^N345K^, resulting in complete mortality, Joardar et al. (2015) screened 1,200 FDA-approved drugs to determine if any improve survival. This screen identified several antidiabetic drugs such as thiazolidinediones, sulfonylureas, and biguanides, with the most potent effect exhibited by the thiazolidinedione pioglitazone. Pioglitazone prevented locomotor deficits and glial death, but could not prevent the death of motor neurons, and was ineffective in preventing disease pathology in a transgenic SOD1-G85R model. This finding contradicts two studies which have shown the benefit of pioglitazone to rescue locomotor deficits, prevent motor neuron death, and improve survival in transgenic SOD1-G93A mice, however, there was no reduction in SOD1 expression, and SOD1 aggregation was not characterized (Kiaei et al., 2005; Schütz et al., 2005).

Using *C. elegans* expressing mutant TDP-43-A315T, Bose et al. (2019) screened a library containing 3,765 novel molecules and identified 27 hit compounds. Validation in a toxic gain-of-function TDP-43-G348C zebrafish model identified four of these hits which improved locomotion. One compound, TRVA242 was identified as the most effective compound and rescued locomotion, improved motor neuron survival, and rescued neuromuscular junction deficits in the aforementioned *C. elegans* and zebrafish model, as well as in SOD1 and C9ORF72 zebrafish models of ALS, and in SOD1-G37R mice.

#### Fused in Sarcoma

High-throughput screening to investigate therapies targeting FUS in ALS using iPSC-derived motor neurons have been performed. A GFP-tagged FUS^P525L^ expressing iPSC cell line treated with sodium arsenite was used to screen ~1,000 small molecules to investigate stress granule formation *via* imaging (Marrone et al., 2018). This screen identified 69 compounds, and further assessment validated 13 molecules targeting the PI3K/AKT/mTOR pathway, with the mTOR inhibitor rapamycin reducing stress granule formation and promoting survival of motor neurons generated from the iPSCs. Whilst the effect of rapamycin on protein aggregation was not characterized, rapamycin did upregulate autophagy, a mechanism important in the clearance of protein aggregates (Ramesh and Pandey, 2017). As mentioned above, the dopamine receptor antagonist ropinirole prevented ALS pathology in iPSC-derived motor neurons from fALS patients with TDP-43 (M337V or Q343R) mutations, and similarly, ropinirole prevented neurite regression, stress granule formation, FUS aggregation, and prevented cell death in iPSC-derived motor neurons from fALS patients with FUS (H517D) mutations (Fujimori et al., 2018).

Currently, there are no high-throughput studies *in vivo* focusing solely on FUS. However, as mentioned above the *C. elegans*, *D. melanogaster* and zebrafish models of FUS represent ideal models to investigate potential drugs, with Vaccaro et al. (2012a) demonstrating methylene blue prevented oxidative stress and improved locomotor defects in both a *C. elegans* and zebrafish model. Additionally, Joardar et al. (2015) did demonstrate that pioglitazone rescues locomotor function in transgenic FUS^P525L^ *Drosophila*, but does not impact survival.

### Diacetylbis(*N*(4)-Methylthiosemicarbazonato)-CuII (CuATSM)

#### Superoxide Dismutase-1

Numerous *in vitro* models exist to explore the role of SOD1 aggregation in ALS pathogenesis. In NSC-34 cells, CuATSM decreased aggregation and increased survival in cells transiently transfected with GFP-tagged mutant A4V, C6G, E100G, G37R, G93A, and V148G SOD1, but not those transfected with H46R, G85R, or G127X (Farrawell et al., 2019). Importantly, the mutants that were not responsive to CuATSM treatment reduce or ablate the ability of SOD1 to bind copper (Carrì et al., 1994). This data suggests that the activity of CuATSM in this model is to facilitate the delivery of copper to SOD1 and thereby limit the pool of immature copper-free SOD1 that is more prone to aggregate (Tokuda et al., 2018).

Dermal application of CuATSM improved survival in a SOD1-G93A mouse model, with CuATSM-treated mice living a median 155 days, compared with vehicle-treated controls with a median lifespan of 133 days (Williams et al., 2016). Oral administration of CuATSM delayed neurological damage and improved latency to fall using a rotarod test, with CuATSM mice displaying these symptoms at 113 days compared with 100 days in vehicle-treated mice. CuATSM treated mice also demonstrated an improved mean survival time of 143 days compared with 129 days in vehicle-treated mice (Hilton et al., 2017). Conversely, CuATSM-treated mice demonstrated significantly increased relative abundance of mutant SOD1-G93A, SOD1 activity, and copper in spinal cords compared with vehicle-treated mice. This increase can be attributed to CuATSM facilitating increased copper delivery to SOD1 *via* CCS, which can improve copper-dependent SOD1 activity, an effect that protects motor neurons and improves disease outcomes (Soon et al., 2011; Roberts et al., 2014; Hilton et al., 2017). This is supported by evidence that ligand-free ATSM does not exert protective effects compared to CuATSM (Vieira et al., 2017).

Similar to the SOD1-G93A mouse model, CuATSM also improves locomotor function (rotarod test) and median survival time (212–248 days depending on dose) in SOD1-G37R mice compared to vehicle control (median survival time 196 days). Administration of riluzole in conjunction with CuATSM did not exhibit any additive effect (McAllum et al., 2013). In a more severe SOD1-G93A mouse model, where mice co-express human CCS, SOD1-G93AxhCCS mice exhibit a mean survival time of 36 days, compared to 226 days in SOD1-G93A mice (Son et al., 2007). Administration of CuATSM in SOD1-G93AxhCCS mice daily from day 5 increased lifespan to >1 year, whereas suspending treatment after 21 days of age until disease onset (80 days) generally led to reduced symptom severity, although some mice developed symptoms and survived for 302–377 days of age (Williams et al., 2016). Complete withdrawal of treatment (after 21 days of age) had a smaller effect on lifespan, with these mice surviving for 122–132 days of age (Williams et al., 2016).

#### Transactive Response DNA-Binding Protein 43

Similar to SOD1, CuATSM has also been explored as a therapy for preventing TDP-43-associated toxicity. In SH-SY5Y neuroblastoma cells, CuATSM protected against paraquat-induced cell death, inhibited aggregation of TDP-43 CTFs, and prevented stress granule formation (Parker et al., 2012). Oral administration of CuATSM also reduced both cytoplasmic accumulation of full-length and C-terminal fragments of TDP-43 in SOD1-G93A mice (Soon et al., 2011) suggesting this therapy may be effective in sporadic ALS patients demonstrating SOD1 and/or TDP-43 pathology.

#### Clinical Trials

CuATSM is currently under investigation in clinical trials, with one phase I study complete (NCT02870634), a continuation of the study ongoing (NCT04313166), and a placebo-controlled study currently recruiting (NCT04082832) at the time of publication.

### Anti-sense Oligonucleotides

Anti-sense oligonucleotides (ASO) can bind to mRNA and reduce protein expression, representing a potential therapeutic strategy (Bennett and Swayze, 2010), and using ASOs targeted at proteins such as SOD1 and TDP-43 can prevent protein aggregation and ALS.

#### Superoxide Dismutase-1

Smith (2006) designed ISIS 333611, an ASO which exhibits a high binding affinity for SOD1. Intraperitoneal injection of ISIS 333611 reduced both mRNA and protein levels of SOD1 in transgenic rats expressing human SOD1-G93A. Also, injection of ISIS 333611 extended the survival of transgenic SOD1-G93A rats to 132 days, compared with rats injected with sham controls, with a mean survival of 122 days. Finally, the transfection of fibroblasts sourced from a human SOD1-A4V ALS patient with ISIS 333611 demonstrated degradation of SOD1 protein (Smith, 2006).

The ASO Tofersen, which targets and degrades SOD1 mRNA, prevented neuron loss in SOD1-G93A mice extending survival by 40 days, and more than 50 days in SOD1-G93A rats (McCampbell et al., 2018). Tofersen was recently used in a Phase-I/II clinical trial, which reduced SOD1 mRNA concentrations at the highest Tofersen concentration administered (100 mg), but was not sufficiently powered to report on motor improvements or survival (Miller et al., 2020), and requires further investigation.

#### Transactive Response DNA-Binding Protein 43

TDP-43 is an essential protein for cellular function, therefore, TDP-43 expression cannot be completely prevented or cell death will occur (Sephton et al., 2012). However, some therapeutic strategies have overcome this by targeting important interactors of TDP-43, such as ataxin-2. Crossing an ataxin-2 deficient mouse strain with a TDP-43 transgenic line led to reduced TDP-43 inclusion formation, reduced stress granule formation, and prolonged survival (Becker et al., 2017). Furthermore, a single intracerebroventricular injection of an ASO that targets ataxin-2 (3 μl of a 15 μg/μl) resulted in reduced ataxin-2 mRNA levels with no effect on TDP-43 mRNA expression. However, there were reduced numbers of TDP-43 inclusions in spinal cords, as well as improved locomotor function and significantly increased mean survival time (>300 days) compared with vehicle-treated mice (29 days).

#### Clinical Trials

Intrathecal injection of ISIS 333611 (doses of 0.15, 0.5, 1.5, and 3 mg) over 11.5 h in phase I, randomized trial of SOD1 fALS was demonstrated to be well tolerated, however, the therapeutic benefits were not measured (Miller et al., 2013). With regards to ASO targeting ataxin-2, due to the injection route chosen and the timing of injection in mice (neonatal), the feasibility of this ASO needs to be carefully considered.

### CLR01

Protein folding in cells is dysregulated in ALS, resulting in protein aggregation. The design of “molecular tweezers” that interact with assembling proteins and acts as a pharmacological chaperone represents a strategy to stabilize this process and prevent aggregation (Klärner and Kahlert, 2003). The molecular tweezer CLR01 inhibits amyloid-protein self-assembly (Fokkens et al., 2005), is well-tolerated in mice (Attar et al., 2014), and represents a potential therapy for ALS.

#### Superoxide Dismutase-1

Malik et al. (2019) demonstrated that CLR01 binds to SOD1 and, using *in vitro* fibrillation assays, showed that CLR01 treatment reduced aggregation of wild-type SOD1 as well as mutant SOD1-E21K, SOD1-H46R, SOD1-D76Y, and SOD1-G93A. Immunohistochemical analysis demonstrated that CLR01 reduced SOD1 aggregates in microglia, but failed to improve locomotor function or survival in SOD1-G93A mice irrespective of the dose used (0.5 or 5 mg/kg—daily subcutaneous injection; Malik et al., 2019).

#### Transactive Response DNA-Binding Protein 43

In a recent study, Monaco et al. (2020) demonstrated that TDP-43 is present in amyloid aggregates in a murine model of the lysosomal storage disease mucopolysaccharidosis (MPS) type IIIA, and aggregates could be reduced by CLR01; however, there was no evidence of CLR01 directly interacting with TDP-43.

## 4,5-Bis[(*N*-Carboxy Methyl Imidazolium)Methylacridine] Dibromide (AIM4)

### Transactive Response DNA-Binding Protein 43

Prasad et al. (2016) screened a range of acridine derivatives to determine the effect on TDP-43 aggregation. They found that the acridine derivative, [4,5-bis[(N-carboxy methyl imidazolium)methylacridine] dibromide (AIM4) reduced aggregation of C-terminal TDP-43 in yeast. A recent follow-up study found that AIM4 could prevent the fibrillation of purified TDP-43^(194–414)^ (Girdhar et al., 2020). However, further validation of the therapeutic potential and assessment of the toxicity of this drug *in vivo* has not been conducted at this time.

## Therapies Targeting Proteostatic Impairment

### Arimoclomol

#### Superoxide Dismutase-1

Hydroxylamine derivatives are a family of compounds that up-regulate the expression of heat shock proteins (HSPs; Vígh et al., 1997). The hydroxylamine derivative arimoclomol differs from other derivatives in that it up-regulates HSP expression only in cells under stress (Hargitai et al., 2003), and works by activating heat shock factor 1 and promoting up-regulation of HSP70 and HSP90 (Kalmar et al., 2014). The up-regulation of these HSPs by arimoclomol reduces SOD1 aggregation. Intraperitoneal injection of arimoclomol into SOD1-G93A mice improved motor neuron survival and increased mean survival to 153 days, compared with untreated SOD1-G93A mice (with a mean survival of 125 days; Kieran et al., 2004; Kalmar et al., 2008). Importantly, this effect was observed if treatment was administered early or at disease onset, and the effect was observed regardless of sex (Kieran et al., 2004; Kalmar et al., 2008).

#### Transactive Response DNA-Binding Protein 43

Activation of HIF-1 is important in maintaining the solubility of TDP-43 and preventing aggregation, as well as maintaining cell survival in SH-SY5Y neuroblastoma cells (Chen et al., 2016; Lin et al., 2016). In a murine model of TDP-43-acetylation, which causes TDP-43 to localize in the cytosol and form inclusions, activation of HIF-1 reduced TDP-43 inclusion formation. The authors suggest this could be achieved with the use of arimoclomol, however, these experiments are yet to be performed (Wang et al., 2017).

#### Fused in Sarcoma

In iPSC-derived spinal motor neurons, arimoclomol was able to retain FUS in the nucleus, and restore the DNA-damage response in cells expressing ALS-mutant FUS^R521H^ (Kuta et al., 2020).

#### Clinical Trials

Arimoclomol has been used in clinical trials for ALS and is well-tolerated in doses up to 200 mg thrice daily, however, the evidence of a benefit in patients remains inconclusive (Benatar et al., 2018). For a recent review of proteostasis-targeting therapies in clinical trials see McAlary et al. (2019a).

### Pridopidine

The Sigma-1 receptor (S1R) is an important chaperone that prevents protein aggregation (Hong et al., 2017), suppresses ROS production (Meunier and Hayashi, 2010), and maintains cell survival (Hayashi and Su, 2007). S1Rs are reduced in spinal cords of ALS patients (Prause et al., 2013), and S1R KO mice exhibit locomotor deficiencies and MN loss (Bernard-Marissal et al., 2015). Pridopidine is a Sigma-1 receptor agonist (Sahlholm et al., 2015), and represents a potential therapeutic for ALS.

#### Superoxide Dismutase-1

Ionescu et al. (2019) demonstrated that incubation of primary SOD1-G93A motor neuron cultures with pridopidine reduces MN death and restores axonal transport and MN junction activity. Furthermore, subcutaneous administration of pridopidine (daily, 3 or 30 mg/kg) in SOD1-G93A mice reduced SOD1 aggregation in spinal cords, prevented muscle fiber atrophy, and preserved NM junctions, but pridopidine treatment alone did not lead to improved survival of SOD1-G93A mice (Ionescu et al., 2019).

#### Clinical Trials

Pridopidine is well tolerated (45, 67.5, 90, and 112.5 mg taken orally twice daily) in Huntington’s disease patients (Reilmann et al., 2019). A clinical trial of pridopidine in ALS is set to commence at Massachusetts General Hospital.

## Conclusion

Years of intensive study into the disease mechanisms of ALS strongly implicates a pathological role for both declining proteostasis and prion-like propagation. There are intimate links between both of these concepts that explain much of the pathological manifestation of ALS. For example, the observation of proteinaceous inclusions in affected tissues, the apparent spread of pathology through neuroanatomically connected tracts, and the preferential degeneration of motor neurons over other cell types. SOD1 and TDP-43 can misfold and aggregate into conformations that can propagate within and between cells in a prion-like manner, as evidenced by the studies mentioned above ranging from biophysical assays of protein stability to whole-organism studies of aggregate seeding. Indeed, this knowledge of proteostasis dysfunction and protein aggregation has led to the development and examination of multiple therapeutics for ALS. The questions that remain, however, are what the conformation of these spreading strains are, the precise mechanism by which they contribute to pathology, and if targeted therapies can be designed to prevent their formation or spread. Also, a question of significant importance to the field is whether FUS can spread pathology from cell-to-cell in a pathological prion-like manner. This knowledge would unify our understanding of the underlying pathomechanisms in ALS.

## Author Contributions

LM directed the manuscript. All authors wrote and edited the manuscript. NRC and JJY conceptualized the manuscript. All authors contributed to the article and approved the submitted version.

## Conflict of Interest

The authors declare that the research was conducted in the absence of any commercial or financial relationships that could be construed as a potential conflict of interest.

## References

[B1] Abdolvahabi A. Shi Y. Chuprin A. Rasouli S. Shaw B. F. (2016). Stochastic formation of fibrillar and amorphous superoxide dismutase oligomers linked to amyotrophic lateral sclerosis. ACS Chem. Neurosci. 7, 799–810. 10.1021/acschemneuro.6b00048 26979728

[B2] Abdolvahabi A. Shi Y. Rasouli S. Croom C. M. Aliyan A. Martí A. A.. (2017). Kaplan-meier meets chemical kinetics: intrinsic rate of SOD1 amyloidogenesis decreased by subset of als mutations and cannot fully explain age of disease onset. ACS Chem. Neurosci. 8, 1378–1389. 10.1021/acschemneuro.7b00029 28290665

[B3] Abdolvahabi A. Shi Y. Rhodes N. R. Cook N. P. Martí A. A. Shaw B. F. (2015). Arresting amyloid with Coulomb’s law: acetylation of ALS-linked SOD1 by aspirin impedes aggregation. Biophys. J. 108, 1199–1212. 10.1016/j.bpj.2015.01.014 25762331 PMC4375441

[B4] Abe K. Itoyama Y. Sobue G. Tsuji S. Aoki M. Doyu M.. (2014). Confirmatory double-blind, parallel-group, placebo-controlled study of efficacy and safety of edaravone (MCI-186) in amyotrophic lateral sclerosis patients. Amyotroph. Lateral Scler. Frontotemporal Degener. 15, 610–617. 10.3109/21678421.2014.959024 25286015 PMC4266079

[B5] Abel O. Shatunov A. Jones A. R. Andersen P. M. Powell J. F. Al-Chalabi A. (2013). Development of a smartphone app for a genetics website: the amyotrophic lateral sclerosis online genetics database (ALSoD). JMIR Mhealth Uhealth 1:e18. 10.2196/mhealth.2706 25098641 PMC4114449

[B6] Afroz T. Hock E.-M. Ernst P. Foglieni C. Jambeau M. Gilhespy L. A. B.. (2017). Functional and dynamic polymerization of the ALS-linked protein TDP-43 antagonizes its pathologic aggregation. Nat. Commun. 8:45. 10.1038/s41467-017-00062-0 28663553 PMC5491494

[B7] Alberti S. Saha S. Woodruff J. B. Franzmann T. M. Wang J. Hyman A. A. (2018). A user’s guide for phase separation assays with purified proteins. J. Mol. Biol. 430, 4806–4820. 10.1016/j.jmb.2018.06.038 29944854 PMC6215329

[B8] Andersson M. K. Ståhlberg A. Arvidsson Y. Olofsson A. Semb H. Stenman G.. (2008). The multifunctional FUS, EWS and TAF15 proto-oncoproteins show cell type-specific expression patterns and involvement in cell spreading and stress response. BMC Cell Biol. 9:37. 10.1186/1471-2121-9-37 18620564 PMC2478660

[B9] Anisimova A. S. Alexandrov A. I. Makarova N. E. Gladyshev V. N. Dmitriev S. E. (2018). Protein synthesis and quality control in aging. Aging 10, 4269–4288. 10.18632/aging.101721 30562164 PMC6326689

[B10] Anzai I. Toichi K. Tokuda E. Mukaiyama A. Akiyama S. Furukawa Y. (2016). Screening of drugs inhibiting *in vitro* oligomerization of Cu/Zn-superoxide dismutase with a mutation causing amyotrophic lateral sclerosis. Front. Mol. Biosci. 3:40. 10.3389/fmolb.2016.00040 27556028 PMC4977284

[B11] Anzai I. Tokuda E. Mukaiyama A. Akiyama S. Endo F. Yamanaka K.. (2017). A misfolded dimer of Cu/Zn-superoxide dismutase leading to pathological oligomerization in amyotrophic lateral sclerosis. Protein Sci. 26, 484–496. 10.1002/pro.3094 27977888 PMC5326558

[B12] Arai T. Hasegawa M. Akiyama H. Ikeda K. Nonaka T. Mori H.. (2006). TDP-43 is a component of ubiquitin-positive tau-negative inclusions in frontotemporal lobar degeneration and amyotrophic lateral sclerosis. Biochem. Biophys. Res. Commun. 351, 602–611. 10.1016/j.bbrc.2006.10.093 17084815

[B13] Arnesano F. Banci L. Bertini I. Martinelli M. Furukawa Y. O’Halloran T. V. (2004). The unusually stable quaternary structure of human Cu,Zn-superoxide dismutase 1 is controlled by both metal occupancy and disulfide status. J. Biol. Chem. 279, 47998–48003. 10.1074/jbc.M406021200 15326189

[B14] Arosio P. Knowles T. P. J. Linse S. (2015). On the lag phase in amyloid fibril formation. Phys. Chem. Chem. Phys. 17, 7606–7618. 10.1039/c4cp05563b 25719972 PMC4498454

[B15] Attar A. Chan W.-T. C. Klärner F.-G. Schrader T. Bitan G. (2014). Safety and pharmacological characterization of the molecular tweezer CLR01—a broad-spectrum inhibitor of amyloid proteins’ toxicity. BMC Pharmacol. Toxicol. 15:23. 10.1186/2050-6511-15-23 24735982 PMC3996151

[B16] Axelrod D. Koppel D. E. Schlessinger J. Elson E. Webb W. W. (1976). Mobility measurement by analysis of fluorescence photobleaching recovery kinetics. Biophys. J. 16, 1055–1069. 10.1016/S0006-3495(76)85755-4 786399 PMC1334945

[B17] Ayala Y. M. De Conti L. Avendaño-Vázquez S. E. Dhir A. Romano M. D’Ambrogio A.. (2011). TDP-43 regulates its mRNA levels through a negative feedback loop. EMBO J. 30, 277–288. 10.1038/emboj.2010.310 21131904 PMC3025456

[B18] Ayers J. I. Fromholt S. Koch M. Debosier A. Mcmahon B. Xu G.. (2014). Experimental transmissibility of mutant SOD1 motor neuron disease. Acta Neuropathol. 128, 791–803. 10.1007/s00401-014-1342-7 25262000

[B19] Ayers J. I. Fromholt S. E. O’Neal V. M. Diamond J. H. Borchelt D. R. (2016). Prion-like propagation of mutant SOD1 misfolding and motor neuron disease spread along neuroanatomical pathways. Acta Neuropathol. 131, 103–114. 10.1007/s00401-015-1514-0 26650262 PMC4699876

[B20] Ayers J. I. McMahon B. Gill S. Lelie H. L. Fromholt S. Brown H.. (2017). Relationship between mutant Cu/Zn superoxide dismutase 1 maturation and inclusion formation in cell models. J. Neurochem. 140, 140–150. 10.1111/jnc.13864 27727458 PMC5283795

[B21] Babin P. J. Goizet C. Raldúa D. (2014). Zebrafish models of human motor neuron diseases: advantages and limitations. Prog. Neurobiol. 118, 36–58. 10.1016/j.pneurobio.2014.03.001 24705136

[B22] Babinchak W. M. Haider R. Dumm B. K. Sarkar P. Surewicz K. Choi J.-K.. (2019). The role of liquid-liquid phase separation in aggregation of the TDP-43 low-complexity domain. J. Biol. Chem. 294, 6306–6317. 10.1074/jbc.RA118.007222 30814253 PMC6484124

[B23] Bahadorani S. Mukai S. T. Rabie J. Beckman J. S. Phillips J. P. Hilliker A. J. (2013). Expression of zinc-deficient human superoxide dismutase in *Drosophila* neurons produces a locomotor defect linked to mitochondrial dysfunction. Neurobiol. Aging 34, 2322–2330. 10.1016/j.neurobiolaging.2013.03.024 23601674 PMC4145400

[B24] Balchin D. Hayer-Hartl M. Hartl F. U. (2016). *In vivo* aspects of protein folding and quality control. Science 353:aac4354. 10.1126/science.aac4354 27365453

[B25] Banani S. F. Lee H. O. Hyman A. A. Rosen M. K. (2017). Biomolecular condensates: organizers of cellular biochemistry. Nat. Rev. Mol. Cell Biol. 18, 285–298. 10.1038/nrm.2017.7 28225081 PMC7434221

[B26] Baskoylu S. N. Yersak J. O’Hern P. Grosser S. Simon J. Kim S.. (2018). Single copy/knock-in models of ALS SOD1 in *C. elegans* suggest loss and gain of function have different contributions to cholinergic and glutamatergic neurodegeneration. PLoS Genet. 14:e1007682. 10.1371/journal.pgen.1007682 30296255 PMC6200258

[B27] Baughman H. E. R. Clouser A. F. Klevit R. E. Nath A. (2018). HspB1 and Hsc70 chaperones engage distinct tau species and have different inhibitory effects on amyloid formation. J. Biol. Chem. 293, 2687–2700. 10.1074/jbc.M117.803411 29298892 PMC5827454

[B28] Becker L. A. Huang B. Bieri G. Ma R. Knowles D. A. Jafar-Nejad P.. (2017). Therapeutic reduction of ataxin-2 extends lifespan and reduces pathology in TDP-43 mice. Nature 544, 367–371. 10.1038/nature22038 28405022 PMC5642042

[B29] Bellen H. J. Tong C. Tsuda H. (2010). 100 years of *Drosophila* research and its impact on vertebrate neuroscience: a history lesson for the future. Nat. Rev. Neurosci. 11, 514–522. 10.1038/nrn2839 20383202 PMC4022039

[B30] Belly A. Moreau-Gachelin F. Sadoul R. Goldberg Y. (2005). Delocalization of the multifunctional RNA splicing factor TLS/FUS in hippocampal neurones: exclusion from the nucleus and accumulation in dendritic granules and spine heads. Neurosci. Lett. 379, 152–157. 10.1016/j.neulet.2004.12.071 15843054

[B31] Belzil V. V. St-Onge J. Daoud H. Desjarlais A. Bouchard J.-P. Dupré N.. (2011). Identification of a FUS splicing mutation in a large family with amyotrophic lateral sclerosis. J. Hum. Genet. 56, 247–249. 10.1038/jhg.2010.162 21160488

[B32] Benatar M. Wuu J. Andersen P. M. Atassi N. David W. Cudkowicz M.. (2018). Randomized, double-blind, placebo-controlled trial of arimoclomol in rapidly progressive ALS. Neurology 90, e565–e574. 10.1212/WNL.0000000000004960 29367439 PMC5818014

[B33] Bennett C. F. Swayze E. E. (2010). RNA targeting therapeutics: molecular mechanisms of antisense oligonucleotides as a therapeutic platform. Annu. Rev. Pharmacol. Toxicol. 50, 259–293. 10.1146/annurev.pharmtox.010909.105654 20055705

[B34] Bensimon G. Lacomblez L. Meininger V. (1994). A controlled trial of riluzole in amyotrophic lateral sclerosis. N. Engl. J. Med. 330, 585–591. 10.1056/NEJM199403033300901 8302340

[B35] Bergh J. Zetterström P. Andersen P. M. Brännström T. Graffmo K. S. Jonsson P. A.. (2015). Structural and kinetic analysis of protein-aggregate strains *in vivo* using binary epitope mapping. Proc. Natl. Acad. Sci. U S A 112, 4489–4494. 10.1073/pnas.1419228112 25802384 PMC4394267

[B36] Bernard-Marissal N. Médard J.-J. Azzedine H. Chrast R. (2015). Dysfunction in endoplasmic reticulum-mitochondria crosstalk underlies SIGMAR1 loss of function mediated motor neuron degeneration. Brain 138, 875–890. 10.1093/brain/awv008 25678561

[B37] Berning B. A. Walker A. K. (2019). The pathobiology of TDP-43 C-terminal fragments in ALS and FTLD. Front. Neurosci. 13:335. 10.3389/fnins.2019.00335 31031584 PMC6470282

[B38] Bhatia N. K. Modi P. Sharma S. Deep S. (2020). Quercetin and baicalein act as potent antiamyloidogenic and fibril destabilizing agents for SOD1 fibrils. ACS Chem. Neurosci. 11, 1129–1138. 10.1021/acschemneuro.9b00677 32208672

[B39] Bhatia N. K. Srivastava A. Katyal N. Jain N. Khan M. A. I. Kundu B.. (2015). Curcumin binds to the pre-fibrillar aggregates of Cu/Zn superoxide dismutase (SOD1) and alters its amyloidogenic pathway resulting in reduced cytotoxicity. Biochim. Biophys. Acta 1854, 426–436. 10.1016/j.bbapap.2015.01.014 25666897

[B40] Bidhendi E. Bergh J. Zetterström P. Forsberg K. Pakkenberg B. Andersen P. M.. (2018). Mutant superoxide dismutase aggregates from human spinal cord transmit amyotrophic lateral sclerosis. Acta Neuropathol. 136, 939–953. 10.1007/s00401-018-1915-y 30284034 PMC6280858

[B41] Bidhendi E. E. Bergh J. Zetterström P. Andersen P. M. Marklund S. L. Brännström T. (2016). Two superoxide dismutase prion strains transmit amyotrophic lateral sclerosis-like disease. J. Clin. Invest. 126, 2249–2253. 10.1172/JCI84360 27140399 PMC4887173

[B42] Bigio E. H. Wu J. Y. Deng H.-X. Bit-Ivan E. N. Mao Q. Ganti R.. (2013). Inclusions in frontotemporal lobar degeneration with TDP-43 proteinopathy (FTLD-TDP) and amyotrophic lateral sclerosis (ALS), but not FTLD with FUS proteinopathy (FTLD-FUS), have properties of amyloid. Acta Neuropathol. 125, 463–465. 10.1007/s00401-013-1089-6 23378033 PMC3593646

[B43] Bille A. Jensen K. S. Mohanty S. Akke M. Irbäck A. (2019). Stability and local unfolding of SOD1 in the presence of protein crowders. J. Phys. Chem. B 123, 1920–1930. 10.1021/acs.jpcb.8b10774 30753785

[B44] Binger K. J. Ecroyd H. Yang S. Carver J. A. Howlett G. J. Griffin M. D. W. (2013). Avoiding the oligomeric state: αB-crystallin inhibits fragmentation and induces dissociation of apolipoprotein C-II amyloid fibrils. FASEB J. 27, 1214–1222. 10.1096/fj.12-220657 23159935

[B45] Bogaert E. Boeynaems S. Kato M. Guo L. Caulfield T. R. Steyaert J.. (2018). Molecular dissection of FUS points at synergistic effect of low-complexity domains in toxicity. Cell Rep. 24, 529.4–537.4. 10.1016/j.celrep.2018.06.070 30021151 PMC6077250

[B46] Bose P. Tremblay E. Maois C. Narasimhan V. Armstrong G. A. B. Liao M.. (2019). The novel small molecule TRVA242 stabilizes neuromuscular junction defects in multiple animal models of amyotrophic lateral sclerosis. Neurotherapeutics 16, 1149–1166. 10.1007/s13311-019-00765-w 31342410 PMC6985319

[B47] Boyce M. Bryant K. F. Jousse C. Long K. Harding H. P. Scheuner D.. (2005). A selective inhibitor of eIF2α dephosphorylation protects cells from ER stress. Science 307, 935–939. 10.1126/science.1101902 15705855

[B48] Boyd J. D. Lee P. Feiler M. S. Zauur N. Liu M. Concannon J.. (2014). A high-content screen identifies novel compounds that inhibit stress-induced TDP-43 cellular aggregation and associated cytotoxicity. J. Biomol. Screen. 19, 44–56. 10.1177/1087057113501553 24019256 PMC3913261

[B49] Braak H. Brettschneider J. Ludolph A. C. Lee V. M. Trojanowski J. Q. Del Tredici K. (2013). Amyotrophic lateral sclerosis—a model of corticofugal axonal spread. Nat. Rev. Neurol. 9, 708–714. 10.1038/nrneurol.2013.221 24217521 PMC3943211

[B50] Brand A. H. Perrimon N. (1993). Targeted gene expression as a means of altering cell fates and generating dominant phenotypes. Development 118, 401–415. 8223268 10.1242/dev.118.2.401

[B51] Brettschneider J. Arai K. Del Tredici K. Toledo J. B. Robinson J. L. Lee E. B.. (2014). TDP-43 pathology and neuronal loss in amyotrophic lateral sclerosis spinal cord. Acta Neuropathol. 128, 423–437. 10.1007/s00401-014-1299-6 24916269 PMC4384652

[B52] Brettschneider J. Del Tredici K. Toledo J. B. Robinson J. L. Irwin D. J. Grossman M.. (2013). Stages of pTDP-43 pathology in amyotrophic lateral sclerosis. Ann. Neurol. 74, 20–38. 10.1002/ana.23937 23686809 PMC3785076

[B53] Britton S. Dernoncourt E. Delteil C. Froment C. Schiltz O. Salles B.. (2014). DNA damage triggers SAF-A and RNA biogenesis factors exclusion from chromatin coupled to R-loops removal. Nucleic Acids Res. 42, 9047–9062. 10.1093/nar/gku601 25030905 PMC4132723

[B54] Bronk P. Wenniger J. J. Dawson-Scully K. Guo X. Hong S. Atwood H. L.. (2001). *Drosophila* Hsc70–4 is critical for neurotransmitter exocytosis *in vivo*. Neuron 30, 475–488. 10.1016/s0896-6273(01)00292-6 11395008

[B55] Broom H. R. Rumfeldt J. A. O. Vassall K. A. Meiering E. M. (2015). Destabilization of the dimer interface is a common consequence of diverse ALS-associated mutations in metal free SOD1. Protein Sci. 24, 2081–2089. 10.1002/pro.2803 26362407 PMC4815230

[B56] Broom H. R. Vassall K. A. Rumfeldt J. A. O. Doyle C. M. Tong M. S. Bonner J. M.. (2016). Combined isothermal titration and differential scanning calorimetry define three-state thermodynamics of fALS-associated mutant apo SOD1 dimers and an increased population of folded monomer. Biochemistry 55, 519–533. 10.1021/acs.biochem.5b01187 26710831

[B57] Broom W. J. Auwarter K. E. Ni J. Russel D. E. Yeh L.-A. Maxwell M. M.. (2006). Two approaches to drug discovery in SOD1-mediated ALS. J. Biomol. Screen. 11, 729–735. 10.1177/1087057106290937 16928982

[B58] Bruijn L. I. Becher M. W. Lee M. K. Anderson K. L. Jenkins N. A. Copeland N. G.. (1997). ALS-linked SOD1 mutant G85R mediates damage to astrocytes and promotes rapidly progressive disease with SOD1-containing inclusions. Neuron 18, 327–338. 10.1016/s0896-6273(00)80272-x 9052802

[B59] Bruijn L. I. Houseweart M. K. Kato S. Anderson K. L. Anderson S. D. Ohama E.. (1998). Aggregation and motor neuron toxicity of an ALS-linked SOD1 mutant independent from wild-type SOD1. Science 281, 1851–1854. 10.1126/science.281.5384.1851 9743498

[B60] Bryson H. M. Fulton B. Benfield P. (1996). Riluzole. A review of its pharmacodynamic and pharmacokinetic properties and therapeutic potential in amyotrophic lateral sclerosis. Drugs 52, 549–563. 10.2165/00003495-199652040-00010 8891467

[B61] Buratti E. Brindisi A. Giombi M. Tisminetzky S. Ayala Y. M. Baralle F. E. (2005). TDP-43 binds heterogeneous nuclear ribonucleoprotein A/B through its C-terminal tail: an important region for the inhibition of cystic fibrosis transmembrane conductance regulator exon 9 splicing. J. Biol. Chem. 280, 37572–37584. 10.1074/jbc.M505557200 16157593

[B62] Buratti E. Dörk T. Zuccato E. Pagani F. Romano M. Baralle F. E. (2001). Nuclear factor TDP-43 and SR proteins promote *in vitro* and *in vivo* CFTR exon 9 skipping. EMBO J. 20, 1774–1784. 10.1093/emboj/20.7.1774 11285240 PMC145463

[B63] Burk K. Pasterkamp R. J. (2019). Disrupted neuronal trafficking in amyotrophic lateral sclerosis. Acta Neuropathol. 137, 859–877. 10.1007/s00401-019-01964-7 30721407 PMC6531423

[B64] Burkhardt M. F. Martinez F. J. Wright S. Ramos C. Volfson D. Mason M.. (2013). A cellular model for sporadic ALS using patient-derived induced pluripotent stem cells. Mol. Cell. Neurosci. 56, 355–364. 10.1016/j.mcn.2013.07.007 23891805 PMC4772428

[B65] Byström R. Andersen P. M. Gröbner G. Oliveberg M. (2010). SOD1 mutations targeting surface hydrogen bonds promote amyotrophic lateral sclerosis without reducing apo-state stability. J. Biol. Chem. 285, 19544–19552. 10.1007/s00216-015-8947-0 20189984 PMC2885233

[B66] Cao Q. Boyer D. R. Sawaya M. R. Ge P. Eisenberg D. S. (2019). Cryo-EM structures of four polymorphic TDP-43 amyloid cores. Nat. Struct. Mol. Biol. 26, 619–627. 10.1038/s41594-019-0248-4 31235914 PMC7047951

[B67] Cao X. Jin X. Liu B. (2020). The involvement of stress granules in aging and aging-associated diseases. Aging Cell 19:e13136. 10.1111/acel.13136 32170904 PMC7189987

[B68] Capper M. J. Wright G. S. A. Barbieri L. Luchinat E. Mercatelli E. McAlary L.. (2018). The cysteine-reactive small molecule ebselen facilitates effective SOD1 maturation. Nat. Commun. 9:1693. 10.1038/s41467-018-04114-x 29703933 PMC5923229

[B69] Carrì M. T. Teresa Carrì M. Battistoni A. Polizio F. Desideri A. Rotilio G. (1994). Impaired copper binding by the H46R mutant of human Cu,Zn superoxide dismutase, involved in amyotrophic lateral sclerosis. FEBS Lett. 356, 314–316. 10.1016/0014-5793(94)01295-4 7805862

[B70] Carrì M. T. Valle C. Bozzo F. Cozzolino M. (2015). Oxidative stress and mitochondrial damage: importance in non-SOD1 ALS. Front. Cell. Neurosci. 9:41. 10.3389/fncel.2015.00041 25741238 PMC4330888

[B71] Casci I. Pandey U. B. (2015). A fruitful endeavor: modeling ALS in the fruit fly. Brain Res. 1607, 47–74. 10.1016/j.brainres.2014.09.064 25289585 PMC4385417

[B72] Cashman N. R. Durham H. D. Blusztajn J. K. Oda K. Tabira T. Shaw I. T.. (1992). Neuroblastoma × spinal cord (NSC) hybrid cell lines resemble developing motor neurons. Dev. Dyn. 194, 209–221. 10.1002/aja.1001940306 1467557

[B73] Ceroni M. Safar J. Piccardo P. Liberski P. P. Pergami P. Gibbs C. J. (1996). “Cellular and scrapie prion protein immunolocalization and *in vitro* amyloid formation,” in Bovine Spongiform Encephalopathy, ed. Gibbs C. J. (New York, NY: Springer), 338–356.

[B74] Chakrabarti O. Ashok A. Hegde R. S. (2009). Prion protein biosynthesis and its emerging role in neurodegeneration. Trends Biochem. Sci. 34, 287–295. 10.1016/j.tibs.2009.03.001 19447626 PMC3132587

[B75] Chan P. K. Chattopadhyay M. Sharma S. Souda P. Gralla E. B. Borchelt D. R.. (2013). Structural similarity of wild-type and ALS-mutant superoxide dismutase-1 fibrils using limited proteolysis and atomic force microscopy. Proc. Natl. Acad. Sci. U S A 110, 10934–10939. 10.1073/pnas.1309613110 23781106 PMC3704032

[B78] Chang L. Monteiro M. J. (2015). Defective proteasome delivery of polyubiquitinated proteins by ubiquilin-2 proteins containing ALS mutations. PLoS One 10:e0130162. 10.1371/journal.pone.0130162 26075709 PMC4468220

[B77] Chang H. C. Newmyer S. L. Hull M. J. Ebersold M. Schmid S. L. Mellman I. (2002). Hsc70 is required for endocytosis and clathrin function in *Drosophila*. J. Cell Biol. 159, 477–487. 10.1083/jcb.200205086 12427870 PMC2173062

[B76] Chang C.-K. Wu T.-H. Wu C.-Y. Chiang M.-H. Toh E. K.-W. Hsu Y.-C.. (2012). The N-terminus of TDP-43 promotes its oligomerization and enhances DNA binding affinity. Biochem. Biophys. Res. Commun. 425, 219–224. 10.1016/j.bbrc.2012.07.071 22835933

[B79] Chantadul V. Wright G. S. A. Amporndanai K. Shahid M. Antonyuk S. V. Washbourn G.. (2020). Ebselen as template for stabilization of A4V mutant dimer for motor neuron disease therapy. Commun. Biol. 3:97. 10.1038/s42003-020-0826-3 32139772 PMC7058017

[B80] Chattopadhyay M. Durazo A. Sohn S. H. Strong C. D. Gralla E. B. Whitelegge J. P.. (2008). Initiation and elongation in fibrillation of ALS-linked superoxide dismutase. Proc. Natl. Acad. Sci. U S A 105, 18663–18668. 10.1073/pnas.0807058105 19022905 PMC2585484

[B81] Chattopadhyay M. Nwadibia E. Strong C. D. Gralla E. B. Valentine J. S. Whitelegge J. P. (2015). The disulfide bond, but not zinc or dimerization, controls initiation and seeded growth in amyotrophic lateral sclerosis-linked Cu, Zn superoxide dismutase (SOD1) fibrillation. J. Biol. Chem. 290, 30624–30636. 10.1074/jbc.M115.666503 26511321 PMC4683282

[B85] Chen Y. Cohen T. J. (2019). Aggregation of the nucleic acid-binding protein TDP-43 occurs *via* distinct routes that are coordinated with stress granule formation. J. Biol. Chem. 294, 3696–3706. 10.1074/jbc.RA118.006351 30630951 PMC6416430

[B82] Chen A. K.-H. Lin R. Y.-Y. Hsieh E. Z.-J. Tu P.-H. Chen R. P.-Y. Liao T.-Y.. (2010). Induction of amyloid fibrils by the C-terminal fragments of TDP-43 in amyotrophic lateral sclerosis. J. Am. Chem. Soc. 132, 1186–1187. 10.1021/ja9066207 20055380

[B83] Chen H.-J. Mitchell J. C. Novoselov S. Miller J. Nishimura A. L. Scotter E. L.. (2016). The heat shock response plays an important role in TDP-43 clearance: evidence for dysfunction in amyotrophic lateral sclerosis. Brain 139, 1417–1432. 10.1093/brain/aww028 26936937 PMC4845254

[B84] Chen H.-J. Topp S. D. Hui H. S. Zacco E. Katarya M. Mcloughlin C.. (2019). RRM adjacent TARDBP mutations disrupt RNA binding and enhance TDP-43 proteinopathy. Brain 142, 3753–3770. 10.1093/brain/awz313 31605140 PMC6885686

[B86] Cheng J. North B. J. Zhang T. Dai X. Tao K. Guo J.. (2018). The emerging roles of protein homeostasis-governing pathways in Alzheimer’s disease. Aging Cell 17:e12801. 10.1111/acel.12801 29992725 PMC6156496

[B87] Chia R. Tattum M. H. Jones S. Collinge J. Fisher E. M. C. Jackson G. S. (2010). Superoxide dismutase 1 and tgSOD1 mouse spinal cord seed fibrils, suggesting a propagative cell death mechanism in amyotrophic lateral sclerosis. PLoS One 5:e10627. 10.1371/journal.pone.0010627 20498711 PMC2869360

[B88] Chiang C.-H. Grauffel C. Wu L.-S. Kuo P.-H. Doudeva L. G. Lim C.. (2016). Structural analysis of disease-related TDP-43 D169G mutation: linking enhanced stability and caspase cleavage efficiency to protein accumulation. Sci. Rep. 6:21581. 10.1038/srep21581 26883171 PMC4756693

[B89] Chiò A. Logroscino G. Hardiman O. Swingler R. Mitchell D. Beghi E.. (2009a). Prognostic factors in ALS: a critical review. Amyotroph. Lateral Scler. 10, 310–323. 10.3109/17482960802566824 19922118 PMC3515205

[B90] Chiò A. Restagno G. Brunetti M. Ossola I. Calvo A. Mora G.. (2009b). Two Italian kindreds with familial amyotrophic lateral sclerosis due to FUS mutation. Neurobiol. Aging 30, 1272–1275. 10.1016/j.neurobiolaging.2009.05.001 19450904 PMC2771748

[B91] Chiti F. Dobson C. M. (2017). Protein misfolding, amyloid formation, and human disease: a summary of progress over the last decade. Annu. Rev. Biochem. 86, 27–68. 10.1146/annurev-biochem-061516-045115 28498720

[B92] Ciryam P. Lambert-Smith I. A. Bean D. M. Freer R. Cid F. Tartaglia G. G.. (2017). Spinal motor neuron protein supersaturation patterns are associated with inclusion body formation in ALS. Proc. Natl. Acad. Sci. U S A 114, E3935–E3943. 10.1073/pnas.1613854114 28396410 PMC5441770

[B93] Cohen S. I. A. Vendruscolo M. Dobson C. M. Knowles T. P. J. (2011). Nucleated polymerisation in the presence of pre-formed seed filaments. Int. J. Mol. Sci. 12, 5844–5852. 10.3390/ijms12095844 22016630 PMC3189754

[B94] Conicella A. E. Zerze G. H. Mittal J. Fawzi N. L. (2016). ALS mutations disrupt phase separation mediated by α-helical structure in the TDP-43 low-complexity C-terminal domain. Structure 24, 1537–1549. 10.1016/j.str.2016.07.007 27545621 PMC5014597

[B96] Cook S. J. Jarrell T. A. Brittin C. A. Wang Y. Bloniarz A. E. Yakovlev M. A.. (2019). Whole-animal connectomes of both *Caenorhabditis elegans* sexes. Nature 571, 63–71. 10.1038/s41586-019-1352-7 31270481 PMC6889226

[B95] Cook C. N. Wu Y. Odeh H. M. Gendron T. F. Jansen-West K. Del Rosso G.. (2020). *C9orf72* poly(GR) aggregation induces TDP-43 proteinopathy. Sci. Transl. Med. 12:eabb3774. 10.1126/scitranslmed.abb3774 32878979 PMC7989020

[B97] Cox D. Whiten D. R. Brown J. W. P. Horrocks M. H. San Gil R. Dobson C. M.. (2018). The small heat shock protein Hsp27 binds α-synuclein fibrils, preventing elongation and cytotoxicity. J. Biol. Chem. 293, 4486–4497. 10.1074/jbc.M117.813865 29382725 PMC5868268

[B98] Coyne A. N. Lorenzini I. Chou C.-C. Torvund M. Rogers R. S. Starr A.. (2017). Post-transcriptional inhibition of Hsc70–4/HSPA8 expression leads to synaptic vesicle cycling defects in multiple models of ALS. Cell Rep. 21, 110–125. 10.1016/j.celrep.2017.09.028 28978466 PMC5679478

[B99] Cragnaz L. Klima R. De Conti L. Romano G. Feiguin F. Buratti E.. (2015). An age-related reduction of brain TBPH/TDP-43 levels precedes the onset of locomotion defects in a *Drosophila* ALS model. Neuroscience 311, 415–421. 10.1016/j.neuroscience.2015.10.037 26518462

[B100] Cragnaz L. Klima R. Skoko N. Budini M. Feiguin F. Baralle F. E. (2014). Aggregate formation prevents dTDP-43 neurotoxicity in the *Drosophila melanogaster* eye. Neurobiol. Dis. 71, 74–80. 10.1016/j.nbd.2014.07.009 25088712

[B101] Crosby K. Crown A. M. Roberts B. L. Brown H. Ayers J. I. Borchelt D. R. (2018). Loss of charge mutations in solvent exposed Lys residues of superoxide dismutase 1 do not induce inclusion formation in cultured cell models. PLoS One 13:e0206751. 10.1371/journal.pone.0206751 30399166 PMC6219784

[B102] Crown A. McAlary L. Fagerli E. Brown H. Yerbury J. J. Galaleldeen A.. (2020). Tryptophan residue 32 in human Cu-Zn superoxide dismutase modulates prion-like propagation and strain selection. PLoS One 15:e0227655. 10.1371/journal.pone.0227655 31999698 PMC6991973

[B103] Culik R. M. Sekhar A. Nagesh J. Deol H. Rumfeldt J. A. O. Meiering E. M.. (2018). Effects of maturation on the conformational free-energy landscape of SOD1. Proc. Natl. Acad. Sci. U S A 115, E2546–E2555. 10.1073/pnas.1721022115 29483249 PMC5856554

[B104] Danielsson J. Mu X. Lang L. Wang H. Binolfi A. Theillet F.-X.. (2015). Thermodynamics of protein destabilization in live cells. Proc. Natl. Acad. Sci. U S A 112, 12402–12407. 10.1073/pnas.1511308112 26392565 PMC4603463

[B105] Dao T. P. Kolaitis R. M. Kim H. J. O’Donovan K. Martyniak B. Colicino E.. (2018). Ubiquitin modulates liquid-liquid phase separation of UBQLN2 *via* disruption of multivalent interactions. Mol. Cell 69, 965.e6–978.e6. 10.1016/j.molcel.2018.02.004 29526694 PMC6181577

[B106] Day R. N. Davidson M. W. (2009). The fluorescent protein palette: tools for cellular imaging. Chem. Soc. Rev. 38, 2887–2921. 10.1039/b901966a 19771335 PMC2910338

[B107] Dejesus-Hernandez M. Kocerha J. Finch N. Crook R. Baker M. Desaro P.. (2010). *De novo* truncating FUS gene mutation as a cause of sporadic amyotrophic lateral sclerosis. Hum. Mutat. 31, E1377–E1389. 10.1002/humu.21241 20232451 PMC2922682

[B108] Dejesus-Hernandez M. Mackenzie I. R. Boeve B. F. Boxer A. L. Baker M. Rutherford N. J.. (2011). Expanded GGGGCC hexanucleotide repeat in noncoding region of C9ORF72 causes chromosome 9p-linked FTD and ALS. Neuron 72, 245–256. 10.1016/j.neuron.2011.09.011 21944778 PMC3202986

[B109] Deng H.-X. Chen W. Hong S.-T. Boycott K. M. Gorrie G. H. Siddique N.. (2011). Mutations in UBQLN2 cause dominant X-linked juvenile and adult-onset ALS and ALS/dementia. Nature 477, 211–215. 10.1038/nature10353 21857683 PMC3169705

[B110] Deng H.-X. Shi Y. Furukawa Y. Zhai H. Fu R. Liu E.. (2006). Conversion to the amyotrophic lateral sclerosis phenotype is associated with intermolecular linked insoluble aggregates of SOD1 in mitochondria. Proc. Natl. Acad. Sci. U S A 103, 7142–7147. 10.1073/pnas.0602046103 16636275 PMC1447523

[B111] DiDonato M. Craig L. Huff M. E. Thayer M. M. Cardoso R. M. F. Kassmann C. J.. (2003). ALS mutants of human superoxide dismutase form fibrous aggregates *via* framework destabilization. J. Mol. Biol. 332, 601–615. 10.1016/s0022-2836(03)00889-1 12963370

[B112] Dietzl G. Chen D. Schnorrer F. Su K.-C. Barinova Y. Fellner M.. (2007). A genome-wide transgenic RNAi library for conditional gene inactivation in *Drosophila*. Nature 448, 151–156. 10.1038/nature05954 17625558

[B113] Dikic I. Elazar Z. (2018). Mechanism and medical implications of mammalian autophagy. Nat. Rev. Mol. Cell Biol. 19, 349–364. 10.1038/s41580-018-0003-4 29618831

[B114] Dimitriadi M. Hart A. C. (2010). Neurodegenerative disorders: insights from the nematode *Caenorhabditis elegans*. Neurobiol. Dis. 40, 4–11. 10.1016/j.nbd.2010.05.012 20493260 PMC2926245

[B115] Dini Modigliani S. Morlando M. Errichelli L. Sabatelli M. Bozzoni I. (2014). An ALS-associated mutation in the FUS 3′-UTR disrupts a microRNA-FUS regulatory circuitry. Nat. Commun. 5:4335. 10.1038/ncomms5335 25004804

[B116] Durham H. D. Roy J. Dong L. Figlewicz D. A. (1997). Aggregation of mutant Cu/Zn superoxide dismutase proteins in a culture model of ALS. J. Neuropathol. Exp. Neurol. 56, 523–530. 10.1097/00005072-199705000-00008 9143265

[B117] Ebstein S. Y. Yagudayeva I. Shneider N. A. (2019). Mutant TDP-43 causes early-stage dose-dependent motor neuron degeneration in a TARDBP knockin mouse model of ALS. Cell Rep. 26, 364.e4–373.e4. 10.1016/j.celrep.2018.12.045 30625319

[B148] Edaravone Acute Infarction Study Group. (2003). Effect of a novel free radical scavenger, edaravone (MCI-186), on acute brain infarction. Randomized, placebo-controlled, double-blind study at multicenters. Cerebrovasc. Dis. 15, 222–229. 10.1159/000069318 12715790

[B118] Egawa N. Kitaoka S. Tsukita K. Naitoh M. Takahashi K. Yamamoto T.. (2012). Drug screening for ALS using patient-specific induced pluripotent stem cells. Sci. Transl. Med. 4:145ra104. 10.1126/scitranslmed.3004052 22855461

[B119] Eisenberg D. Jucker M. (2012). The amyloid state of proteins in human diseases. Cell 148, 1188–1203. 10.1016/j.cell.2012.02.022 22424229 PMC3353745

[B120] Eisenberg D. S. Sawaya M. R. (2017). Structural studies of amyloid proteins at the molecular level. Annu. Rev. Biochem. 86, 69–95. 10.1146/annurev-biochem-061516-045104 28125289

[B121] Fang M. Y. Markmiller S. Vu A. Q. Javaherian A. Dowdle W. E. Jolivet P.. (2019). Small-molecule modulation of TDP-43 recruitment to stress granules prevents persistent TDP-43 accumulation in ALS/FTD. Neuron 103, 802.e11–819.e11. 10.1016/j.neuron.2019.05.048 31272829 PMC6728177

[B122] Farrawell N. E. Lambert-Smith I. Mitchell K. McKenna J. McAlary L. Ciryam P.. (2018). SOD1A4Vaggregation alters ubiquitin homeostasis in a cell model of ALS. J. Cell Sci. 131:jcs209122. 10.1242/jcs.209122 29748379 PMC6919637

[B123] Farrawell N. E. Lambert-Smith I. A. Warraich S. T. Blair I. P. Saunders D. N. Hatters D. M.. (2015). Distinct partitioning of ALS associated TDP-43, FUS and SOD1 mutants into cellular inclusions. Sci. Rep. 5:13416. 10.1038/srep13416 26293199 PMC4544019

[B124] Farrawell N. E. Yerbury M. R. Plotkin S. S. Mcalary L. Yerbury J. J. (2019). CuATSM protects against the *in vitro* cytotoxicity of wild-type-like copper-zinc superoxide dismutase mutants but not mutants that disrupt metal binding. ACS Chem. Neurosci. 10, 1555–1564. 10.1021/acschemneuro.8b00527 30462490

[B125] Fatima M. Tan R. Halliday G. M. Kril J. J. (2015). Spread of pathology in amyotrophic lateral sclerosis: assessment of phosphorylated TDP-43 along axonal pathways. Acta Neuropathol. Commun. 3:47. 10.1186/s40478-015-0226-y 26216351 PMC4517552

[B126] Feiler M. S. Strobel B. Freischmidt A. Helferich A. M. Kappel J. Brewer B. M.. (2015). TDP-43 is intercellularly transmitted across axon terminals. J. Cell Biol. 211, 897–911. 10.1083/jcb.201504057 26598621 PMC4657165

[B127] Fitzpatrick A. W. P. Falcon B. He S. Murzin A. G. Murshudov G. Garringer H. J.. (2017). Cryo-EM structures of tau filaments from Alzheimer’s disease. Nature 547, 185–190. 10.1038/nature23002 28678775 PMC5552202

[B128] Fokkens M. Schrader T. Klärner F.-G. (2005). A molecular tweezer for lysine and arginine. J. Am. Chem. Soc. 127, 14415–14421. 10.1021/ja052806a 16218636

[B129] Fratta P. Sivakumar P. Humphrey J. Lo K. Ricketts T. Oliveira H.. (2018). Mice with endogenous TDP-43 mutations exhibit gain of splicing function and characteristics of amyotrophic lateral sclerosis. EMBO J. 37:e98684. 10.15252/embj.201798684 29764981 PMC5983119

[B130] Freibaum B. D. Taylor J. P. (2017). The role of dipeptide repeats in C9ORF72-related ALS-FTD. Front. Mol. Neurosci. 10:35. 10.3389/fnmol.2017.00035 28243191 PMC5303742

[B131] Freibaum B. D. Chitta R. K. High A. A. Taylor J. P. (2010). Global analysis of TDP-43 interacting proteins reveals strong association with RNA splicing and translation machinery. J. Proteome Res. 9, 1104–1120. 10.1021/pr901076y 20020773 PMC2897173

[B132] Fujii R. Takumi T. (2005). TLS facilitates transport of mRNA encoding an actin-stabilizing protein to dendritic spines. J. Cell Sci. 118, 5755–5765. 10.1242/jcs.02692 16317045

[B133] Fujimori K. Ishikawa M. Otomo A. Atsuta N. Nakamura R. Akiyama T.. (2018). Modeling sporadic ALS in iPSC-derived motor neurons identifies a potential therapeutic agent. Nat. Med. 24, 1579–1589. 10.1038/s41591-018-0140-5 30127392

[B134] Furukawa Y. Kaneko K. Watanabe S. Yamanaka K. Nukina N. (2011). A seeding reaction recapitulates intracellular formation of Sarkosyl-insoluble transactivation response element (TAR) DNA-binding protein-43 inclusions. J. Biol. Chem. 286, 18664–18672. 10.1074/jbc.M111.231209 21454603 PMC3099683

[B135] Furukawa Y. Kaneko K. Yamanaka K. Nukina N. (2010). Mutation-dependent polymorphism of Cu,Zn-superoxide dismutase aggregates in the familial form of amyotrophic lateral sclerosis. J. Biol. Chem. 285, 22221–22231. 10.1074/jbc.M110.113597 20404329 PMC2903422

[B136] Furukawa Y. Kaneko K. Yamanaka K. O’Halloran T. V. Nukina N. (2008). Complete loss of post-translational modifications triggers fibrillar aggregation of SOD1 in the familial form of amyotrophic lateral sclerosis. J. Biol. Chem. 283, 24167–24176. 10.1074/jbc.M802083200 18552350 PMC3259764

[B137] Garnier C. Devred F. Byrne D. Puppo R. Roman A. Y. Malesinski S.. (2017). Zinc binding to RNA recognition motif of TDP-43 induces the formation of amyloid-like aggregates. Sci. Rep. 7:6812. 10.1038/s41598-017-07215-7 28754988 PMC5533730

[B138] Gasset-Rosa F. Lu S. Yu H. Chen C. Melamed Z. E. Guo L.. (2019). Cytoplasmic TDP-43 de-mixing independent of stress granules drives inhibition of nuclear import, loss of nuclear TDP-43, and cell death. Neuron 102, 339.e7–357.e7. 10.1016/j.neuron.2019.02.038 30853299 PMC6548321

[B139] Gertz B. Wong M. Martin L. J. (2012). Nuclear localization of human SOD1 and mutant SOD1-specific disruption of survival motor neuron protein complex in transgenic amyotrophic lateral sclerosis mice. J. Neuropathol. Exp. Neurol. 71, 162–177. 10.1097/NEN.0b013e318244b635 22249462 PMC3432922

[B140] Geser F. Brandmeir N. J. Kwong L. K. Martinez-Lage M. Elman L. McCluskey L.. (2008). Evidence of multisystem disorder in whole-brain map of pathological TDP-43 in amyotrophic lateral sclerosis. Arch. Neurol. 65, 636–641. 10.1001/archneur.65.5.636 18474740

[B141] Gidalevitz T. Krupinski T. Garcia S. Morimoto R. I. (2009). Destabilizing protein polymorphisms in the genetic background direct phenotypic expression of mutant SOD1 toxicity. PLoS Genet. 5:e1000399. 10.1371/journal.pgen.1000399 19266020 PMC2642731

[B142] Girdhar A. Bharathi V. Tiwari V. R. Abhishek S. Deeksha W. Mahawar U. S.. (2020). Computational insights into mechanism of AIM4-mediated inhibition of aggregation of TDP-43 protein implicated in ALS and evidence for *in vitro* inhibition of liquid-liquid phase separation (LLPS) of TDP-43^2C^–A315T by AIM4. Int. J. Biol. Macromol. 147, 117–130. 10.1016/j.ijbiomac.2020.01.032 31917988

[B143] Goldberg A. L. (2003). Protein degradation and protection against misfolded or damaged proteins. Nature 426, 895–899. 10.1038/nature02263 14685250

[B144] Gordon D. Dafinca R. Scaber J. Alegre-Abarrategui J. Farrimond L. Scott C.. (2019). Single-copy expression of an amyotrophic lateral sclerosis-linked TDP-43 mutation (M337V) in BAC transgenic mice leads to altered stress granule dynamics and progressive motor dysfunction. Neurobiol. Dis. 121, 148–162. 10.1016/j.nbd.2018.09.024 30290270

[B145] Grad L. I. Guest W. C. Yanai A. Pokrishevsky E. O’Neill M. A. Gibbs E.. (2011). Intermolecular transmission of superoxide dismutase 1 misfolding in living cells. Proc. Natl. Acad. Sci. U S A 108, 16398–16403. 10.1073/pnas.1102645108 21930926 PMC3182705

[B146] Grad L. I. Yerbury J. J. Turner B. J. Guest W. C. Pokrishevsky E. O’Neill M. A.. (2014). Intercellular propagated misfolding of wild-type Cu/Zn superoxide dismutase occurs *via* exosome-dependent and -independent mechanisms. Proc. Natl. Acad. Sci. U S A 111, 3620–3625. 10.1073/pnas.1312245111 24550511 PMC3948312

[B147] Gregory J. M. Whiten D. R. Brown R. A. Barros T. P. Kumita J. R. Yerbury J. J.. (2017). Clusterin protects neurons against intracellular proteotoxicity. Acta Neuropathol. Commun. 5:81. 10.1186/s40478-017-0481-1 29115989 PMC5678579

[B149] Guenther E. L. Cao Q. Trinh H. Lu J. Sawaya M. R. Cascio D.. (2018a). Atomic structures of TDP-43 LCD segments and insights into reversible or pathogenic aggregation. Nat. Struct. Mol. Biol. 25, 463–471. 10.1038/s41594-018-0064-2 29786080 PMC5990464

[B150] Guenther E. L. Ge P. Trinh H. Sawaya M. R. Cascio D. Boyer D. R.. (2018b). Atomic-level evidence for packing and positional amyloid polymorphism by segment from TDP-43 RRM2. Nat. Struct. Mol. Biol. 25, 311–319. 10.1038/s41594-018-0045-5 29531287 PMC6056015

[B151] Guerrero-Ferreira R. Taylor N. M. Arteni A.-A. Kumari P. Mona D. Ringler P.. (2019). Two new polymorphic structures of human full-length α-synuclein fibrils solved by cryo-electron microscopy. eLife 8:e48907. 10.7554/eLife.48907 31815671 PMC6957273

[B152] Guo L. Kim H. J. Wang H. Monaghan J. Freyermuth F. Sung J. C.. (2018). Nuclear-import receptors reverse aberrant phase transitions of RNA-binding proteins with prion-like domains. Cell 173, 677.e20–692.e20. 10.1016/j.cell.2018.03.002 29677512 PMC5911940

[B153] Guo W. Chen Y. Zhou X. Kar A. Ray P. Chen X.. (2011). An ALS-associated mutation affecting TDP-43 enhances protein aggregation, fibril formation and neurotoxicity. Nat. Struct. Mol. Biol. 18, 822–830. 10.1038/nsmb.2053 21666678 PMC3357956

[B154] Guo W. Fumagalli L. Prior R. Van Den Bosch L. (2017). Current advances and limitations in modeling ALS/FTD in a dish using induced pluripotent stem cells. Front. Neurosci. 11:671. 10.3389/fnins.2017.00671 29326542 PMC5733489

[B155] Gurney M. E. Pu H. Chiu A. Y. Dal Canto M. C. Polchow C. Y. Alexander D. D.. (1994). Motor neuron degeneration in mice that express a human Cu,Zn superoxide dismutase mutation. Science 264, 1772–1775. 10.1126/science.8209258 8209258

[B156] Hales K. G. Korey C. A. Larracuente A. M. Roberts D. M. (2015). Genetics on the Fly: a primer on thedrosophilamodel system. Genetics 201, 815–842. 10.1534/genetics.115.183392 26564900 PMC4649653

[B157] Hallewell R. A. Masiarz F. R. Najarian R. C. Purna J. P. Quiroga M. R. Randolph A.. (1985). Human Cu/Zn superoxide dismutase cDNA: isolation of clones synthesising high levels of active or inactive enzyme from an expression library. Nucleic Acids Res. 13, 2017–2034. 10.1093/nar/13.6.2017 3889846 PMC341132

[B158] Hallewell R. A. Mills R. Tekamp-Olson P. Blacher R. Rosenberg S. Ötting F.. (1987). Amino terminal acetylation of authentic human Cu,Zn superoxide dismutase produced in yeast. Nat. Biotechnol. 5, 363–366. 10.1038/nbt0487-363

[B159] Han T. W. Kato M. Xie S. Wu L. C. Mirzaei H. Pei J.. (2012). Cell-free formation of RNA granules: bound RNAs identify features and components of cellular assemblies. Cell 149, 768–779. 10.1016/j.cell.2012.04.016 22579282

[B160] Hargitai J. Lewis H. Boros I. Rácz T. Fiser A. Kurucz I.. (2003). Bimoclomol, a heat shock protein co-inducer, acts by the prolonged activation of heat shock factor-1. Biochem. Biophys. Res. Commun. 307, 689–695. 10.1016/s0006-291x(03)01254-3 12893279

[B161] Harrison A. F. Shorter J. (2017). RNA-binding proteins with prion-like domains in health and disease. Biochem. J. 474, 1417–1438. 10.1042/BCJ20160499 28389532 PMC5639257

[B162] Hartl F. U. Ulrich Hartl F. Bracher A. Hayer-Hartl M. (2011). Molecular chaperones in protein folding and proteostasis. Nature 475, 324–332. 10.1038/nature10317 21776078

[B163] Hatahet F. Ruddock L. W. (2009). Protein disulfide isomerase: a critical evaluation of its function in disulfide bond formation. Antioxid. Redox Signal. 11, 2807–2850. 10.1089/ars.2009.2466 19476414

[B164] Hayashi T. Su T.-P. (2007). Sigma-1 receptor chaperones at the ER- mitochondrion interface regulate Ca^2+^ signaling and cell survival. Cell 131, 596–610. 10.1016/j.cell.2007.08.036 17981125

[B165] Hefferon T. W. Groman J. D. Yurk C. E. Cutting G. R. (2004). A variable dinucleotide repeat in the CFTR gene contributes to phenotype diversity by forming RNA secondary structures that alter splicing. Proc. Natl. Acad. Sci. U S A 101, 3504–3509. 10.1073/pnas.0400182101 14993601 PMC373492

[B166] Heppert J. K. Dickinson D. J. Pani A. M. Higgins C. D. Steward A. Ahringer J.. (2016). Comparative assessment of fluorescent proteins for *in vivo* imaging in an animal model system. Mol. Biol. Cell 27, 3385–3394. 10.1091/mbc.E16-01-0063 27385332 PMC5221575

[B167] Hilton J. B. Mercer S. W. Lim N. K. H. Faux N. G. Buncic G. Beckman J. S.. (2017). Cu^II^(atsm) improves the neurological phenotype and survival of SOD1^G93A^ mice and selectively increases enzymatically active SOD1 in the spinal cord. Sci. Rep. 7:42292. 10.1038/srep42292 28205575 PMC5304223

[B168] Hipp M. S. Kasturi P. Hartl F. U. (2019). The proteostasis network and its decline in ageing. Nat. Rev. Mol. Cell Biol. 20, 421–435. 10.1038/s41580-019-0101-y 30733602

[B169] Hobert O. (2013). “The neuronal genome of *Caenorhabditis elegans*,” in WormBook, ed. The C. elegans Research Community, WormBook. Available online at: http://www.wormbook.org. 10.1895/wormbook.1.161.1 PMC4781646 24081909

[B170] Hock E.-M. Polymenidou M. (2016). Prion-like propagation as a pathogenic principle in frontotemporal dementia. J. Neurochem. 138, 163–183. 10.1111/jnc.13668 27502124 PMC6680357

[B171] Hofweber M. Hutten S. Bourgeois B. Spreitzer E. Niedner-Boblenz A. Schifferer M.. (2018). Phase separation of FUS is suppressed by its nuclear import receptor and arginine methylation. Cell 173, 706.e13–719.e13. 10.1016/j.cell.2018.03.004 29677514

[B172] Hong J. Wang L. Zhang T. Zhang B. Chen L. (2017). Sigma-1 receptor knockout increases α-synuclein aggregation and phosphorylation with loss of dopaminergic neurons in substantia nigra. Neurobiol. Aging 59, 171–183. 10.1016/j.neurobiolaging.2017.08.007 28870519

[B173] Huang Y.-C. Lin K.-F. He R.-Y. Tu P.-H. Koubek J. Hsu Y.-C.. (2013). Inhibition of TDP-43 aggregation by nucleic acid binding. PLoS One 8:e64002. 10.1371/journal.pone.0064002 23737961 PMC3667863

[B174] Huelgas-Morales G. Silva-García C. G. Salinas L. S. Greenstein D. Navarro R. E. (2016). The stress granule RNA-binding protein TIAR-1 protects female germ cells from heat shock in *Caenorhabditis elegans*. G3 6, 1031–1047. 10.1534/g3.115.026815 26865701 PMC4825639

[B175] Hughes M. P. Sawaya M. R. Boyer D. R. Goldschmidt L. Rodriguez J. A. Cascio D.. (2018). Atomic structures of low-complexity protein segments reveal kinked β sheets that assemble networks. Science 359, 698–701. 10.1126/science.aan6398 29439243 PMC6192703

[B176] Iko Y. Kodama T. S. Kasai N. Oyama T. Morita E. H. Muto T.. (2004). Domain architectures and characterization of an RNA-binding protein, TLS. J. Biol. Chem. 279, 44834–44840. 10.1074/jbc.M408552200 15299008

[B177] Imamura K. Izumi Y. Watanabe A. Tsukita K. Woltjen K. Yamamoto T.. (2017). The Src/c-Abl pathway is a potential therapeutic target in amyotrophic lateral sclerosis. Sci. Transl. Med. 9:eaaf3962. 10.1126/scitranslmed.aaf3962 28539470

[B178] Ince P. G. Highley J. R. Kirby J. Wharton S. B. Takahashi H. Strong M. J.. (2011). Molecular pathology and genetic advances in amyotrophic lateral sclerosis: an emerging molecular pathway and the significance of glial pathology. Acta Neuropathol. 122, 657–671. 10.1007/s00401-011-0913-0 22105541

[B179] Inukai Y. Nonaka T. Arai T. Yoshida M. Hashizume Y. Beach T. G.. (2008). Abnormal phosphorylation of Ser409/410 of TDP-43 in FTLD-U and ALS. FEBS Lett. 582, 2899–2904. 10.1016/j.febslet.2008.07.027 18656473

[B180] Ionescu A. Gradus T. Altman T. Maimon R. Avraham N. S. Geva M.. (2019). Targeting the sigma-1 receptor *via* pridopidine ameliorates central features of ALS pathology in a SOD1G93A model. Cell Death Dis. 10:210. 10.1038/s41419-019-1451-2 30824685 PMC6397200

[B181] Ip P. Sharda P. R. Cunningham A. Chakrabartty S. Pande V. Chakrabartty A. (2017). Quercitrin and quercetin 3-β-d-glucoside as chemical chaperones for the A4V SOD1 ALS-causing mutant. Protein Eng. Des. Sel. 30, 431–440. 10.1093/protein/gzx025 28475686 PMC5939853

[B182] Ivanova M. I. Sievers S. A. Guenther E. L. Johnson L. M. Winkler D. D. Galaleldeen A.. (2014). Aggregation-triggering segments of SOD1 fibril formation support a common pathway for familial and sporadic ALS. Proc. Natl. Acad. Sci. U S A 111, 197–201. 10.1073/pnas.1320786110 24344300 PMC3890817

[B183] Jäckel S. Summerer A. K. Thömmes C. M. Pan X. Voigt A. Schulz J. B.. (2015). Nuclear import factor transportin and arginine methyltransferase 1 modify FUS neurotoxicity in *Drosophila*. Neurobiol. Dis. 74, 76–88. 10.1016/j.nbd.2014.11.003 25447237

[B184] Jenett A. Rubin G. M. Ngo T.-T. B. Shepherd D. Murphy C. Dionne H.. (2012). A GAL4-driver line resource for *Drosophila* neurobiology. Cell Rep. 2, 991–1001. 10.1016/j.celrep.2012.09.011 23063364 PMC3515021

[B185] Jiang L.-L. Che M.-X. Zhao J. Zhou C.-J. Xie M.-Y. Li H.-Y.. (2013). Structural transformation of the amyloidogenic core region of TDP-43 protein initiates its aggregation and cytoplasmic inclusion. J. Biol. Chem. 288, 19614–19624. 10.1074/jbc.M113.463828 23689371 PMC3707662

[B186] Jiang L.-L. Zhao J. Yin X.-F. He W.-T. Yang H. Che M.-X.. (2016). Two mutations G335D and Q343R within the amyloidogenic core region of TDP-43 influence its aggregation and inclusion formation. Sci. Rep. 6:23928. 10.1038/srep23928 27030292 PMC4814915

[B187] Joardar A. Menzl J. Podolsky T. C. Manzo E. Estes P. S. Ashford S.. (2015). PPAR γ activation is neuroprotective in a *Drosophila* model of ALS based on TDP-43. Hum. Mol. Genet. 24, 1741–1754. 10.1093/hmg/ddu587 25432537 PMC4381760

[B188] Jones G. W. Tuite M. F. (2005). Chaperoning prions: the cellular machinery for propagating an infectious protein? Bioessays 27, 823–832. 10.1002/bies.20267 16015602

[B189] Kaganovich D. Kopito R. Frydman J. (2008). Misfolded proteins partition between two distinct quality control compartments. Nature 454, 1088–1095. 10.1038/nature07195 18756251 PMC2746971

[B190] Kaletta T. Hengartner M. O. (2006). Finding function in novel targets: *C. elegans* as a model organism. Nat. Rev. Drug Discov. 5, 387–398. 10.1038/nrd2031 16672925

[B191] Kalmar B. Lu C.-H. Greensmith L. (2014). The role of heat shock proteins in Amyotrophic lateral sclerosis: the therapeutic potential of arimoclomol. Pharmacol. Ther. 141, 40–54. 10.1016/j.pharmthera.2013.08.003 23978556

[B192] Kalmar B. Novoselov S. Gray A. Cheetham M. E. Margulis B. Greensmith L. (2008). Late stage treatment with arimoclomol delays disease progression and prevents protein aggregation in the SOD1G93A mouse model of ALS. J. Neurochem. 107, 339–350. 10.1111/j.1471-4159.2008.05595.x 18673445

[B193] Kato M. Han T. W. Xie S. Shi K. Du X. Wu L. C.. (2012). Cell-free formation of RNA granules: low complexity sequence domains form dynamic fibers within hydrogels. Cell 149, 753–767. 10.1016/j.cell.2012.04.017 22579281 PMC6347373

[B194] Kato S. Takikawa M. Nakashima K. Hirano A. Cleveland D. W. Kusaka H.. (2000). New consensus research on neuropathological aspects of familial amyotrophic lateral sclerosis with superoxide dismutase 1 (SOD1) gene mutations: inclusions containing SOD1 in neurons and astrocytes. Amyotroph. Lateral Scler. Other Motor Neuron Disord. 1, 163–184. 10.1080/14660820050515160 11464950

[B195] Kenyon C. Chang J. Gensch E. Rudner A. Tabtiang R. (1993). A *C. elegans* mutant that lives twice as long as wild type. Nature 366, 461–464. 10.1038/366461a0 8247153

[B196] Kerman A. Liu H.-N. Croul S. Bilbao J. Rogaeva E. Zinman L.. (2010). Amyotrophic lateral sclerosis is a non-amyloid disease in which extensive misfolding of SOD1 is unique to the familial form. Acta Neuropathol. 119, 335–344. 10.1007/s00401-010-0646-5 20111867

[B197] Khan M. A. I. Respondek M. Kjellström S. Deep S. Linse S. Akke M. (2017). Cu/Zn superoxide dismutase forms amyloid fibrils under near-physiological quiescent conditions: the roles of disulfide bonds and effects of denaturant. ACS Chem. Neurosci. 8, 2019–2026. 10.1021/acschemneuro.7b00162 28585802

[B198] Kiaei M. Kipiani K. Chen J. Calingasan N. Y. Beal M. F. (2005). Peroxisome proliferator-activated receptor-γ agonist extends survival in transgenic mouse model of amyotrophic lateral sclerosis. Exp. Neurol. 191, 331–336. 10.1016/j.expneurol.2004.10.007 15649489

[B199] Kieran D. Kalmar B. Dick J. R. T. Riddoch-Contreras J. Burnstock G. Greensmith L. (2004). Treatment with arimoclomol, a coinducer of heat shock proteins, delays disease progression in ALS mice. Nat. Med. 10, 402–405. 10.1038/nm1021 15034571

[B201] Kim T.-Y. Kim E. Yoon S. K. Yoon J.-B. (2008). Herp enhances ER-associated protein degradation by recruiting ubiquilins. Biochem. Biophys. Res. Commun. 369, 741–746. 10.1016/j.bbrc.2008.02.086 18307982

[B202] King O. D. Gitler A. D. Shorter J. (2012). The tip of the iceberg: RNA-binding proteins with prion-like domains in neurodegenerative disease. Brain Res. 1462, 61–80. 10.1016/j.brainres.2012.01.016 22445064 PMC3372647

[B200] Kim H. J. Taylor J. P. (2017). Lost in transportation: nucleocytoplasmic transport defects in ALS and other neurodegenerative diseases. Neuron 96, 285–297. 10.1016/j.neuron.2017.07.029 29024655 PMC5678982

[B203] Klärner F.-G. Kahlert B. (2003). Molecular tweezers and clips as synthetic receptors. Molecular recognition and dynamics in receptor-substrate complexes. Acc. Chem. Res. 36, 919–932. 10.1021/ar0200448 14674783

[B204] Kleiger G. Mayor T. (2014). Perilous journey: a tour of the ubiquitin-proteasome system. Trends Cell Biol. 24, 352–359. 10.1016/j.tcb.2013.12.003 24457024 PMC4037451

[B205] Kleijnen M. F. Shih A. H. Zhou P. Kumar S. Soccio R. E. Kedersha N. L.. (2000). The hPLIC proteins may provide a link between the ubiquitination machinery and the proteasome. Mol. Cell 6, 409–419. 10.1016/s1097-2765(00)00040-x 10983987

[B206] Knowles T. P. J. Buehler M. J. (2011). Nanomechanics of functional and pathological amyloid materials. Nat. Nanotechnol. 6, 469–479. 10.1038/nnano.2011.102 21804553

[B207] Kodali R. Williams A. D. Chemuru S. Wetzel R. (2010). Aβ(1–40) forms five distinct amyloid structures whose β-sheet contents and fibril stabilities are correlated. J. Mol. Biol. 401, 503–517. 10.1016/j.jmb.2010.06.023 20600131 PMC2919579

[B208] Koh S.-H. Lee S. M. Kim H. Y. Lee K.-Y. Lee Y. J. Kim H.-T.. (2006). The effect of epigallocatechin gallate on suppressing disease progression of ALS model mice. Neurosci. Lett. 395, 103–107. 10.1016/j.neulet.2005.10.056 16356650

[B209] Kollmer M. Close W. Funk L. Rasmussen J. Bsoul A. Schierhorn A.. (2019). Cryo-EM structure and polymorphism of Aβ amyloid fibrils purified from Alzheimer’s brain tissue. Nat. Commun. 10:4760. 10.1038/s41467-019-12683-8 31664019 PMC6820800

[B210] Kretschmer B. D. Kratzer U. Schmidt W. J. (1998). Riluzole, a glutamate release inhibitor and motor behavior. Naunyn. Schmiedebergs. Arch. Pharmacol. 358, 181–190. 10.1007/pl00005241 9750003

[B211] Kuo P.-H. Doudeva L. G. Wang Y.-T. Shen C.-K. J. Yuan H. S. (2009). Structural insights into TDP-43 in nucleic-acid binding and domain interactions. Nucleic Acids Res. 37, 1799–1808. 10.1093/nar/gkp013 19174564 PMC2665213

[B212] Kurtishi A. Rosen B. Patil K. S. Alves G. W. Møller S. G. (2019). Cellular proteostasis in neurodegeneration. Mol. Neurobiol. 56, 3676–3689. 10.1007/s12035-018-1334-z 30182337

[B213] Kuta R. Larochelle N. Fernandez M. Pal A. Minotti S. Tibshirani M.. (2020). Depending on the stress, histone deacetylase inhibitors act as heat shock protein co-inducers in motor neurons and potentiate arimoclomol, exerting neuroprotection through multiple mechanisms in ALS models. Cell Stress Chaperones 25, 173–191. 10.1007/s12192-019-01064-1 31900865 PMC6985055

[B214] Kwiatkowski T. J. Jr. Bosco D. A. Leclerc A. L. Tamrazian E. Vanderburg C. R. Russ C.. (2009). Mutations in the FUS/TLS gene on chromosome 16 cause familial amyotrophic lateral sclerosis. Science 323, 1205–1208. 10.1126/science.1166066 19251627

[B215] Kwon I. Kato M. Xiang S. Wu L. Theodoropoulos P. Mirzaei H.. (2013). Phosphorylation-regulated binding of RNA polymerase II to fibrous polymers of low-complexity domains. Cell 155, 1049–1060. 10.1016/j.cell.2013.10.033 24267890 PMC4010232

[B216] Lacomblez L. Bensimon G. Leigh P. N. Guillet P. Meininger V. (1996). Dose-ranging study of riluzole in amyotrophic lateral sclerosis. Amyotrophic Lateral Sclerosis/Riluzole Study Group II. Lancet 347, 1425–1431. 10.1016/s0140-6736(96)91680-3 8676624

[B217] Laferriere F. Polymenidou M. (2015). Advances and challenges in understanding the multifaceted pathogenesis of amyotrophic lateral sclerosis. Swiss Med. Wkly 145:w14054. 10.4414/smw.2015.14054 25635517

[B218] Laferrière F. Maniecka Z. Pérez-Berlanga M. Hruska-Plochan M. Gilhespy L. Hock E.-M.. (2019). TDP-43 extracted from frontotemporal lobar degeneration subject brains displays distinct aggregate assemblies and neurotoxic effects reflecting disease progression rates. Nat. Neurosci. 22, 65–77. 10.1038/s41593-018-0294-y 30559480

[B219] Lai C. H. Chou C. Y. Ch’ang L. Y. Liu C. S. Lin W. (2000). Identification of novel human genes evolutionarily conserved in *Caenorhabditis elegans* by comparative proteomics. Genome Res. 10, 703–713. 10.1101/gr.10.5.703 10810093 PMC310876

[B220] Lang L. Kurnik M. Danielsson J. Oliveberg M. (2012). Fibrillation precursor of superoxide dismutase 1 revealed by gradual tuning of the protein-folding equilibrium. Proc. Natl. Acad. Sci. U S A 109, 17868–17873. 10.1073/pnas.1201795109 22797895 PMC3497812

[B221] Lang L. Zetterström P. Brännström T. Marklund S. L. Danielsson J. Oliveberg M. (2015). SOD1 aggregation in ALS mice shows simplistic test tube behavior. Proc. Natl. Acad. Sci. U S A 112, 9878–9883. 10.1073/pnas.1503328112 26221023 PMC4538623

[B222] Lee E. B. Lee V. M. Y. Trojanowski J. Q. (2012). Gains or losses: molecular mechanisms of TDP43-mediated neurodegeneration. Nat. Rev. Neurosci. 13, 38–50. 10.1038/nrn3121 22127299 PMC3285250

[B223] Lehtonen Š. Sonninen T.-M. Wojciechowski S. Goldsteins G. Koistinaho J. (2019). Dysfunction of cellular proteostasis in Parkinson’s disease. Front. Neurosci. 13:457. 10.3389/fnins.2019.00457 31133790 PMC6524622

[B224] Lepock J. R. Frey H. E. Hallewell R. A. (1990). Contribution of conformational stability and reversibility of unfolding to the increased thermostability of human and bovine superoxide dismutase mutated at free cysteines. J. Biol. Chem. 265, 21612–21618. 2254318

[B226] Li H.-R. Chiang W.-C. Chou P.-C. Wang W.-J. Huang J.-R. (2018). TAR DNA-binding protein 43 (TDP-43) liquid-liquid phase separation is mediated by just a few aromatic residues. J. Biol. Chem. 293, 6090–6098. 10.1074/jbc.AC117.001037 29511089 PMC5912450

[B225] Li B. Ge P. Murray K. A. Sheth P. Zhang M. Nair G.. (2018). Cryo-EM of full-length α-synuclein reveals fibril polymorphs with a common structural kernel. Nat. Commun. 9:3609. 10.1038/s41467-018-05971-2 30190461 PMC6127345

[B227] Li J. Le W. (2013). Modeling neurodegenerative diseases in *Caenorhabditis elegans*. Exp. Neurol. 250, 94–103. 10.1016/j.expneurol.2013.09.024 24095843

[B229] Li J. Li T. Zhang X. Tang Y. Yang J. Le W. (2014). Human superoxide dismutase 1 overexpression in motor neurons of *Caenorhabditis elegans* causes axon guidance defect and neurodegeneration. Neurobiol. Aging 35, 837–846. 10.1016/j.neurobiolaging.2013.09.003 24126158

[B228] Li J. Huang K.-X. Le W.-D. (2013). Establishing a novel *C. elegans* model to investigate the role of autophagy in amyotrophic lateral sclerosis. Acta Pharmacol. Sin. 34, 644–650. 10.1038/aps.2012.190 23503474 PMC3647213

[B230] Li W. Reeb A. N. Lin B. Subramanian P. Fey E. E. Knoverek C. R.. (2017). Heat Shock-induced phosphorylation of TAR DNA-binding protein 43 (TDP-43) by MAPK/ERK kinase regulates TDP-43 function. J. Biol. Chem. 292, 5089–5100. 10.1074/jbc.M116.753913 28167528 PMC5377819

[B231] Liachko N. F. Guthrie C. R. Kraemer B. C. (2010). Phosphorylation promotes neurotoxicity in a *Caenorhabditis elegans* model of TDP-43 proteinopathy. J. Neurosci. 30, 16208–16219. 10.1523/JNEUROSCI.2911-10.2010 21123567 PMC3075589

[B232] Liang V. Ullrich M. Lam H. Chew Y. L. Banister S. Song X.. (2014). Altered proteostasis in aging and heat shock response in *C. elegans* revealed by analysis of the global and de novo synthesized proteome. Cell. Mol. Life Sci. 71, 3339–3361. 10.1007/s00018-014-1558-7 24458371 PMC4131143

[B233] Liddelow S. A. Guttenplan K. A. Clarke L. E. Bennett F. C. Bohlen C. J. Schirmer L.. (2017). Neurotoxic reactive astrocytes are induced by activated microglia. Nature 541, 481–487. 10.1038/nature21029 28099414 PMC5404890

[B235] Lin W.-L. Dickson D. W. (2008). Ultrastructural localization of TDP-43 in filamentous neuronal inclusions in various neurodegenerative diseases. Acta Neuropathol. 116, 205–213. 10.1007/s00401-008-0408-9 18607609 PMC2706695

[B234] Lin P.-Y. Folorunso O. Taglialatela G. Pierce A. (2016). Overexpression of heat shock factor 1 maintains TAR DNA binding protein 43 solubility *via* induction of inducible heat shock protein 70 in cultured cells. J. Neurosci. Res. 94, 671–682. 10.1002/jnr.23725 26994698

[B236] Lindberg M. J. Byström R. Boknäs N. Andersen P. M. Oliveberg M. (2005). Systematically perturbed folding patterns of amyotrophic lateral sclerosis (ALS)-associated SOD1 mutants. Proc. Natl. Acad. Sci. U S A 102, 9754–9759. 10.1073/pnas.0501957102 15987780 PMC1174986

[B237] Lindberg M. J. Tibell L. Oliveberg M. (2002). Common denominator of Cu/Zn superoxide dismutase mutants associated with amyotrophic lateral sclerosis: decreased stability of the apo state. Proc. Natl. Acad. Sci. U S A 99, 16607–16612. 10.1073/pnas.262527099 12482932 PMC139191

[B238] Ling S.-C. Polymenidou M. Cleveland D. W. (2013). Converging mechanisms in ALS and FTD: disrupted RNA and protein homeostasis. Neuron 79, 416–438. 10.1016/j.neuron.2013.07.033 23931993 PMC4411085

[B239] Lippincott-Schwartz J. Altan-Bonnet N. Patterson G. H. (2003). Photobleaching and photoactivation: following protein dynamics in living cells. Nat. Cell Biol. S7–S14. 14562845

[B240] Luchinat E. Barbieri L. Banci L. (2017). A molecular chaperone activity of CCS restores the maturation of SOD1 fALS mutants. Sci. Rep. 7:17433. 10.1038/s41598-017-17815-y 29234142 PMC5727297

[B241] Luchinat E. Barbieri L. Rubino J. T. Kozyreva T. Cantini F. Banci L. (2014). In-cell NMR reveals potential precursor of toxic species from SOD1 fALS mutants. Nat. Commun. 5:5502. 10.1038/ncomms6502 25429517

[B242] Lukavsky P. J. Daujotyte D. Tollervey J. R. Ule J. Stuani C. Buratti E.. (2013). Molecular basis of UG-rich RNA recognition by the human splicing factor TDP-43. Nat. Struct. Mol. Biol. 20, 1443–1449. 10.1038/nsmb.2698 24240615

[B243] Machamer J. B. Collins S. E. Lloyd T. E. (2014). The ALS gene FUS regulates synaptic transmission at the *Drosophila* neuromuscular junction. Hum. Mol. Genet. 23, 3810–3822. 10.1093/hmg/ddu094 24569165 PMC4065154

[B244] Maharana S. Wang J. Papadopoulos D. K. Richter D. Pozniakovsky A. Poser I.. (2018). RNA buffers the phase separation behavior of prion-like RNA binding proteins. Science 360, 918–921. 10.1126/science.aar7366 29650702 PMC6091854

[B245] Malik R. Meng H. Wongkongkathep P. Corrales C. I. Sepanj N. Atlasi R. S.. (2019). The molecular tweezer CLR01 inhibits aberrant superoxide dismutase 1 (SOD1) self-assembly *in vitro* and in the G93A-SOD1 mouse model of ALS. J. Biol. Chem. 294, 3501–3513. 10.1074/jbc.RA118.005940 30602569 PMC6416427

[B246] Mann J. R. Gleixner A. M. Mauna J. C. Gomes E. Dechellis-Marks M. R. Needham P. G.. (2019). RNA binding antagonizes neurotoxic phase transitions of TDP-43. Neuron 102, 321.e8–338.e8. 10.1016/j.neuron.2019.01.048 30826182 PMC6472983

[B247] Marchante R. Beal D. M. Koloteva-Levine N. Purton T. J. Tuite M. F. Xue W.-F. (2017). The physical dimensions of amyloid aggregates control their infective potential as prion particles. eLife 6:e27109. 10.7554/eLife.27109 28880146 PMC5589414

[B248] Marrone L. Poser I. Casci I. Japtok J. Reinhardt P. Janosch A.. (2018). Isogenic FUS-eGFP iPSC reporter lines enable quantification of fus stress granule pathology that is rescued by drugs inducing autophagy. Stem Cell Reports 10, 375–389. 10.1016/j.stemcr.2017.12.018 29358088 PMC5857889

[B249] Mastrocola A. S. Kim S. H. Trinh A. T. Rodenkirch L. A. Tibbetts R. S. (2013). The RNA-binding protein fused in sarcoma (FUS) functions downstream of poly(ADP-ribose) polymerase (PARP) in response to DNA damage. J. Biol. Chem. 288, 24731–24741. 10.1074/jbc.M113.497974 23833192 PMC3750169

[B250] Matsumoto G. Kim S. Morimoto R. I. (2006). Huntingtin and mutant SOD1 form aggregate structures with distinct molecular properties in human cells. J. Biol. Chem. 281, 4477–4485. 10.1074/jbc.M509201200 16371362

[B251] Matsumoto G. Stojanovic A. Holmberg C. I. Kim S. Morimoto R. I. (2005). Structural properties and neuronal toxicity of amyotrophic lateral sclerosis-associated Cu/Zn superoxide dismutase 1 aggregates. J. Cell Biol. 171, 75–85. 10.1083/jcb.200504050 16216923 PMC2171239

[B252] Matus S. Valenzuela V. Medinas D. B. Hetz C. (2013). ER dysfunction and protein folding stress in ALS. Int. J. Cell Biol. 2013:674751. 10.1155/2013/674751 24324498 PMC3845333

[B253] McAlary L. Aquilina J. A. Yerbury J. J. (2016). Susceptibility of mutant SOD1 to form a destabilized monomer predicts cellular aggregation and toxicity *in vitro* but not aggregation propensity. Front. Neurosci. 10:499. 10.3389/fnins.2016.00499 27867347 PMC5095133

[B254] McAlary L. Plotkin S. S. Cashman N. R. (2019a). Emerging developments in targeting proteotoxicity in neurodegenerative diseases. CNS Drugs 33, 883–904. 10.1007/s40263-019-00657-9 31414322 PMC6776490

[B255] McAlary L. Plotkin S. S. Yerbury J. J. Cashman N. R. (2019b). Prion-like propagation of protein misfolding and aggregation in amyotrophic lateral sclerosis. Front. Mol. Neurosci. 12:262. 10.3389/fnmol.2019.00262 31736708 PMC6838634

[B256] McAlary L. Yerbury J. J. Aquilina J. A. (2013). Glutathionylation potentiates benign superoxide dismutase 1 variants to the toxic forms associated with amyotrophic lateral sclerosis. Sci. Rep. 3:3275. 10.1038/srep03275 24253732 PMC3834562

[B257] McAllum E. J. Lim N. K. H. Hickey J. L. Paterson B. M. Donnelly P. S. Li Q.-X.. (2013). Therapeutic effects of CuII(atsm) in the SOD1–G37R mouse model of amyotrophic lateral sclerosis. Amyotroph. Lateral Scler. Frontotemporal Degener. 14, 586–590. 10.3109/21678421.2013.824000 23952668

[B258] McCampbell A. Cole T. Wegener A. J. Tomassy G. S. Setnicka A. Farley B. J.. (2018). Antisense oligonucleotides extend survival and reverse decrement in muscle response in ALS models. J. Clin. Invest. 128, 3558–3567. 10.1172/JCI99081 30010620 PMC6063493

[B259] Mccord J. M. Fridovich I. (1969). Superoxide dismutase. An enzymic function for erythrocuprein (hemocuprein). J. Biol. Chem. 244, 6049–6055. 5389100

[B260] McGown A. Stopford M. J. (2018). High-throughput drug screens for amyotrophic lateral sclerosis drug discovery. Expert Opin. Drug Discov. 13, 1015–1025. 10.1080/17460441.2018.1533953 30317895

[B261] McKinley M. P. Meyer R. K. Kenaga L. Rahbar F. Cotter R. Serban A.. (1991). Scrapie prion rod formation *in vitro* requires both detergent extraction and limited proteolysis. J. Virol. 65, 1340–1351. 10.1128/JVI.65.3.1340-1351.1991 1704926 PMC239910

[B262] Medinas D. B. González J. V. Falcon P. Hetz C. (2017a). Fine-tuning ER stress signal transducers to treat amyotrophic lateral sclerosis. Front. Mol. Neurosci. 10:216. 10.3389/fnmol.2017.00216 28725179 PMC5496948

[B263] Medinas D. B. Valenzuela V. Hetz C. (2017b). Proteostasis disturbance in amyotrophic lateral sclerosis. Hum. Mol. Genet. 26, R91–R104. 10.1093/hmg/ddx274 28977445

[B264] Mejzini R. Flynn L. L. Pitout I. L. Fletcher S. Wilton S. D. Akkari P. A. (2019). ALS genetics, mechanisms and therapeutics: where are we now? Front. Neurosci. 13:1310. 10.3389/fnins.2019.01310 31866818 PMC6909825

[B265] Mello C. C. Kramer J. M. Stinchcomb D. Ambros V. (1991). Efficient gene transfer in *C. elegans*: extrachromosomal maintenance and integration of transforming sequences. EMBO J. 10, 3959–3970. 1935914 10.1002/j.1460-2075.1991.tb04966.x PMC453137

[B266] Mercado P. A. Ayala Y. M. Romano M. Buratti E. Baralle F. E. (2005). Depletion of TDP 43 overrides the need for exonic and intronic splicing enhancers in the human ApoA-II gene. Nucleic Acids Res. 33, 6000–6010. 10.1093/nar/gki897 16254078 PMC1270946

[B267] Meunier J. Hayashi T. (2010). Sigma-1 receptors regulate Bcl-2 expression by reactive oxygen species-dependent transcriptional regulation of nuclear factor κB. J. Pharmacol. Exp. Ther. 332, 388–397. 10.1124/jpet.109.160960 19855099 PMC2812109

[B268] Miguel L. Frébourg T. Campion D. Lecourtois M. (2011). Both cytoplasmic and nuclear accumulations of the protein are neurotoxic in *Drosophila* models of TDP-43 proteinopathies. Neurobiol. Dis. 41, 398–406. 10.1016/j.nbd.2010.10.007 20951205

[B269] Miklos A. C. Sarkar M. Wang Y. Pielak G. J. (2011). Protein crowding tunes protein stability. J. Am. Chem. Soc. 133, 7116–7120. 10.1021/ja200067p 21506571

[B270] Miller T. Cudkowicz M. Shaw P. J. Andersen P. M. Atassi N. Bucelli R. C.. (2020). Phase 1-2 trial of antisense oligonucleotide tofersen for *SOD1* ALS. N. Engl. J. Med. 383, 109–119. 10.1056/NEJMoa2003715 32640130

[B271] Miller T. M. Pestronk A. David W. Rothstein J. Simpson E. Appel S. H.. (2013). An antisense oligonucleotide against SOD1 delivered intrathecally for patients with SOD1 familial amyotrophic lateral sclerosis: a phase 1, randomised, first-in-man study. Lancet Neurol. 12, 435–442. 10.1016/S1474-4422(13)70061-9 23541756 PMC3712285

[B272] Mitchell J. C. Mcgoldrick P. Vance C. Hortobagyi T. Sreedharan J. Rogelj B.. (2013). Overexpression of human wild-type FUS causes progressive motor neuron degeneration in an age- and dose-dependent fashion. Acta Neuropathol. 125, 273–288. 10.1007/s00401-012-1043-z 22961620 PMC3549237

[B273] Mompeán M. Buratti E. Guarnaccia C. Brito R. M. M. Chakrabartty A. Baralle F. E.. (2014). Structural characterization of the minimal segment of TDP-43 competent for aggregation. Arch. Biochem. Biophys. 545, 53–62. 10.1016/j.abb.2014.01.007 24440310

[B274] Mompeán M. Romano V. Pantoja-Uceda D. Stuani C. Baralle F. E. Buratti E.. (2016). The TDP-43 N-terminal domain structure at high resolution. FEBS J. 283, 1242–1260. 10.1111/febs.13651 26756435

[B275] Mompeán M. Romano V. Pantoja-Uceda D. Stuani C. Baralle F. E. Buratti E.. (2017). Point mutations in the N-terminal domain of transactive response DNA-binding protein 43 kDa (TDP-43) compromise its stability, dimerization and functions. J. Biol. Chem. 292, 11992–12006. 10.1074/jbc.M117.775965 28566288 PMC5512090

[B276] Monaco A. Maffia V. Sorrentino N. C. Sambri I. Ezhova Y. Giuliano T.. (2020). The amyloid inhibitor clr01 relieves autophagy and ameliorates neuropathology in a severe lysosomal storage disease. Mol. Ther. 28, 1167–1176. 10.1016/j.ymthe.2020.02.005 32087148 PMC7132627

[B277] Monahan Z. Ryan V. H. Janke A. M. Burke K. A. Rhoads S. N. Zerze G. H.. (2017). Phosphorylation of the FUS low-complexity domain disrupts phase separation, aggregation and toxicity. EMBO J. 36, 2951–2967. 10.15252/embj.201696394 28790177 PMC5641905

[B278] Morales R. (2017). Prion strains in mammals: different conformations leading to disease. PLoS Pathog. 13:e1006323. 10.1371/journal.ppat.1006323 28683090 PMC5500360

[B279] Mori F. Tanji K. Zhang H.-X. Nishihira Y. Tan C.-F. Takahashi H.. (2008). Maturation process of TDP-43-positive neuronal cytoplasmic inclusions in amyotrophic lateral sclerosis with and without dementia. Acta Neuropathol. 116, 193–203. 10.1007/s00401-008-0396-9 18560845

[B280] Morley J. F. Morimoto R. I. (2004). Regulation of longevity in *Caenorhabditis elegans* by heat shock factor and molecular chaperones. Mol. Biol. Cell 15, 657–664. 10.1091/mbc.e03-07-0532 14668486 PMC329286

[B281] Morrice J. R. Gregory-Evans C. Y. Shaw C. A. (2018). Animal models of amyotrophic lateral sclerosis: a comparison of model validity. Neural Regen. Res. 13, 2050–2054. 10.4103/1673-5374.241445 30323119 PMC6199948

[B282] Münch C. O’Brien J. Bertolotti A. (2011). Prion-like propagation of mutant superoxide dismutase-1 misfolding in neuronal cells. Proc. Natl. Acad. Sci. U S A 108, 3548–3553. 10.1073/pnas.1017275108 21321227 PMC3048161

[B283] Murakami T. Yang S.-P. Xie L. Kawano T. Fu D. Mukai A.. (2012). ALS mutations in FUS cause neuronal dysfunction and death in *Caenorhabditis elegans* by a dominant gain-of-function mechanism. Hum. Mol. Genet. 21, 1–9. 10.1093/hmg/ddr417 21949354 PMC3235006

[B284] Murray D. T. Kato M. Lin Y. Thurber K. R. Hung I. Mcknight S. L.. (2017). Structure of FUS protein fibrils and its relevance to self-assembly and phase separation of low-complexity domains. Cell 171, 615.e6–627.e6. 10.1016/j.cell.2017.08.048 28942918 PMC5650524

[B285] Murthy A. C. Dignon G. L. Kan Y. Zerze G. H. Parekh S. H. Mittal J.. (2019). Molecular interactions underlying liquid-liquid phase separation of the FUS low-complexity domain. Nat. Struct. Mol. Biol. 26, 637–648. 10.1038/s41594-019-0250-x 31270472 PMC6613800

[B286] Naiki H. Higuchi K. Hosokawa M. Takeda T. (1989). Fluorometric determination of amyloid fibrils *in vitro* using the fluorescent dye, thioflavin T1. Anal. Biochem. 177, 244–249. 10.1016/0003-2697(89)90046-8 2729542

[B287] N’Diaye E. N. Kajihara K. K. Hsieh I. Morisaki H. Debnath J. Brown E. J. (2009). PLIC proteins or ubiquilins regulate autophagy-dependent cell survival during nutrient starvation. EMBO Rep. 10, 173–179. 10.1038/embor.2008.238 19148225 PMC2637314

[B288] Nedelsky N. B. Taylor J. P. (2019). Bridging biophysics and neurology: aberrant phase transitions in neurodegenerative disease. Nat. Rev. Neurol. 15, 272–286. 10.1038/s41582-019-0157-5 30890779

[B289] Neumann M. Kwong L. K. Lee E. B. Kremmer E. Flatley A. Xu Y.. (2009). Phosphorylation of S409/410 of TDP-43 is a consistent feature in all sporadic and familial forms of TDP-43 proteinopathies. Acta Neuropathol. 117, 137–149. 10.1007/s00401-008-0477-9 19125255 PMC2693625

[B290] Neumann M. Sampathu D. M. Kwong L. K. Truax A. C. Micsenyi M. C. Chou T. T.. (2006). Ubiquitinated TDP-43 in frontotemporal lobar degeneration and amyotrophic lateral sclerosis. Science 314, 130–133. 10.1126/science.1134108 17023659

[B291] Nguyen H. P. Van Broeckhoven C. Van Der Zee J. (2018). ALS genes in the genomic era and their implications for FTD. Trends Genet. 34, 404–423. 10.1016/j.tig.2018.03.001 29605155

[B292] Nomura T. Watanabe S. Kaneko K. Yamanaka K. Nukina N. Furukawa Y. (2014). Intranuclear aggregation of mutant FUS/TLS as a molecular pathomechanism of amyotrophic lateral sclerosis. J. Biol. Chem. 289, 1192–1202. 10.1074/jbc.M113.516492 24280224 PMC3887186

[B293] Nonaka T. Masuda-Suzukake M. Arai T. Hasegawa Y. Akatsu H. Obi T.. (2013). Prion-like properties of pathological TDP-43 aggregates from diseased brains. Cell Rep. 4, 124–134. 10.1016/j.celrep.2013.06.007 23831027

[B294] Nowak R. J. Cuny G. D. Choi S. Lansbury P. T. Ray S. S. (2010). Improving binding specificity of pharmacological chaperones that target mutant superoxide dismutase-1 linked to familial amyotrophic lateral sclerosis using computational methods. J. Med. Chem. 53, 2709–2718. 10.1021/jm901062p 20232802 PMC2881568

[B295] Nussbaum-Krammer C. I. Morimoto R. I. (2014). *Caenorhabditis elegans* as a model system for studying non-cell-autonomous mechanisms in protein-misfolding diseases. Dis. Model. Mech. 7, 31–39. 10.1242/dmm.013011 24396152 PMC3882046

[B296] Oberstadt M. Stieler J. Simpong D. L. Römuß U. Urban N. Schaefer M.. (2018). TDP-43 self-interaction is modulated by redox-active compounds Auranofin, Chelerythrine and Riluzole. Sci. Rep. 8:2248. 10.1038/s41598-018-20565-0 29396541 PMC5797228

[B297] Ogura K.-I. Goshima Y. (2006). The autophagy-related kinase UNC-51 and its binding partner UNC-14 regulate the subcellular localization of the Netrin receptor UNC-5 in *Caenorhabditis elegans*. Development 133, 3441–3450. 10.1242/dev.02503 16887826

[B298] Olsen A. (2006). Using *Caenorhabditis elegans* as a model for aging and age-related diseases. Ann. N Y Acad. Sci. 1067, 120–128. 10.1196/annals.1354.015 16803977

[B299] Osaka M. Ito D. Suzuki N. (2016). Disturbance of proteasomal and autophagic protein degradation pathways by amyotrophic lateral sclerosis-linked mutations in ubiquilin 2. Biochem. Biophys. Res. Commun. 472, 324–331. 10.1016/j.bbrc.2016.02.107 26944018

[B300] Owald D. Lin S. Waddell S. (2015). Light, heat, action: neural control of fruit fly behaviour. Philos. Trans. R. Soc. Lond. B Biol. Sci. 370:20140211. 10.1098/rstb.2014.0211 26240426 PMC4528823

[B301] Owen M. C. Gnutt D. Gao M. Wärmländer S. K. T. S. Jarvet J. Gräslund A.. (2019). Effects of *in vivo* conditions on amyloid aggregation. Chem. Soc. Rev. 48, 3946–3996. 10.1039/c8cs00034d 31192324

[B302] Pan K. M. Baldwin M. Nguyen J. Gasset M. Serban A. Groth D.. (1993). Conversion of α-helices into β-sheets features in the formation of the scrapie prion proteins. Proc. Natl. Acad. Sci. U S A 90, 10962–10966. 10.1073/pnas.90.23.10962 7902575 PMC47901

[B303] Park J.-H. Jang H. R. Lee I. Y. Oh H. K. Choi E.-J. Rhim H.. (2017). Amyotrophic lateral sclerosis-related mutant superoxide dismutase 1 aggregates inhibit 14–3-3-mediated cell survival by sequestration into the JUNQ compartment. Hum. Mol. Genet. 26, 3615–3629. 10.1093/hmg/ddx250 28666328

[B304] Parker S. J. Meyerowitz J. James J. L. Liddell J. R. Nonaka T. Hasegawa M.. (2012). Inhibition of TDP-43 accumulation by bis(thiosemicarbazonato)-copper complexes. PLoS One 7:e42277. 10.1371/journal.pone.0042277 22879928 PMC3411774

[B305] Patel A. Lee H. O. Jawerth L. Maharana S. Jahnel M. Hein M. Y.. (2015). A liquid-to-solid phase transition of the ALS protein FUS accelerated by disease mutation. Cell 162, 1066–1077. 10.1016/j.cell.2015.07.047 26317470

[B306] Patten S. A. Aggad D. Martinez J. Tremblay E. Petrillo J. Armstrong G. A.. (2017). Neuroleptics as therapeutic compounds stabilizing neuromuscular transmission in amyotrophic lateral sclerosis. JCI Insight 2:e97152. 10.1172/jci.insight.97152 29202456 PMC5752378

[B307] Perri E. R. Thomas C. J. Parakh S. Spencer D. M. Atkin J. D. (2016). The unfolded protein response and the role of protein disulfide isomerase in neurodegeneration. Front. Cell Dev. Biol. 3:80. 10.3389/fcell.2015.00080 26779479 PMC4705227

[B308] Petkova A. T. (2005). Self-propagating, molecular-level polymorphism in Alzheimer’s β-amyloid fibrils. Science 307, 262–265. 10.1126/science.1105850 15653506

[B309] Philips T. Bento-Abreu A. Nonneman A. Haeck W. Staats K. Geelen V.. (2013). Oligodendrocyte dysfunction in the pathogenesis of amyotrophic lateral sclerosis. Brain 136, 471–482. 10.1093/brain/aws339 23378219 PMC3572934

[B310] Pokrishevsky E. Hong R. H. Mackenzie I. R. Cashman N. R. (2017). Spinal cord homogenates from SOD1 familial amyotrophic lateral sclerosis induce SOD1 aggregation in living cells. PLoS One 12:e0184384. 10.1371/journal.pone.0184384 28877271 PMC5587256

[B311] Pokrishevsky E. Mcalary L. Farrawell N. E. Zhao B. Sher M. Yerbury J. J.. (2018). Tryptophan 32-mediated SOD1 aggregation is attenuated by pyrimidine-like compounds in living cells. Sci. Rep. 8:15590. 10.1038/s41598-018-32835-y 30349065 PMC6197196

[B312] Polling S. Mok Y.-F. Ramdzan Y. M. Turner B. J. Yerbury J. J. Hill A. F.. (2014). Misfolded polyglutamine, polyalanine and superoxide dismutase 1 aggregate *via* distinct pathways in the cell. J. Biol. Chem. 289, 6669–6680. 10.1074/jbc.M113.520189 24425868 PMC3945328

[B313] Polymenidou M. Lagier-Tourenne C. Hutt K. R. Huelga S. C. Moran J. Liang T. Y.. (2011). Long pre-mRNA depletion and RNA missplicing contribute to neuronal vulnerability from loss of TDP-43. Nat. Neurosci. 14, 459–468. 10.1038/nn.2779 21358643 PMC3094729

[B314] Pons M. Miguel L. Miel C. Avequin T. Juge F. Frebourg T.. (2017). Splicing factors act as genetic modulators of TDP-43 production in a new autoregulatory TDP-43 *Drosophila* model. Hum. Mol. Genet. 26, 3396–3408. 10.1093/hmg/ddx229 28854702

[B315] Pons M. Prieto S. Miguel L. Frebourg T. Campion D. Suñé C.. (2018). Identification of TCERG1 as a new genetic modulator of TDP-43 production in *Drosophila*. Acta Neuropathol. Commun. 6:138. 10.1186/s40478-018-0639-5 30541625 PMC6292132

[B316] Porta S. Xu Y. Restrepo C. R. Kwong L. K. Zhang B. Brown H. J.. (2018). Patient-derived frontotemporal lobar degeneration brain extracts induce formation and spreading of TDP-43 pathology *in vivo*. Nat. Commun. 9:4220. 10.1038/s41467-018-06548-9 30310141 PMC6181940

[B317] Prasad A. Raju G. Sivalingam V. Girdhar A. Verma M. Vats A.. (2016). An acridine derivative, [4,5-bis(*N*-carboxy methyl imidazolium)methylacridine] dibromide, shows anti-TDP-43 aggregation effect in ALS disease models. Sci. Rep. 6:39490. 10.1038/srep39490 28000730 PMC5175139

[B318] Prause J. Goswami A. Katona I. Roos A. Schnizler M. Bushuven E.. (2013). Altered localization, abnormal modification and loss of function of Sigma receptor-1 in amyotrophic lateral sclerosis. Hum. Mol. Genet. 22, 1581–1600. 10.1093/hmg/ddt008 23314020

[B319] Prudencio M. Borchelt D. R. (2011). Superoxide dismutase 1 encoding mutations linked to ALS adopts a spectrum of misfolded states. Mol. Neurodegener. 6:77. 10.1186/1750-1326-6-77 22094223 PMC3248846

[B320] Prudencio M. Hart P. J. Borchelt D. R. Andersen P. M. (2009). Variation in aggregation propensities among ALS-associated variants of SOD1: correlation to human disease. Hum. Mol. Genet. 18, 3217–3226. 10.1093/hmg/ddp260 19483195 PMC2722984

[B321] Prusiner S. B. (1982). Novel proteinaceous infectious particles cause scrapie. Science 216, 136–144. 10.1126/science.6801762 6801762

[B322] Prusiner S. B. (2001). Neurodegenerative diseases and prions. N. Engl. J. Med. 344, 1516–1526. 10.1056/NEJM200105173442006 11357156

[B323] Qamar S. Wang G. Randle S. J. Ruggeri F. S. Varela J. A. Lin J. Q.. (2018). FUS phase separation is modulated by a molecular chaperone and methylation of arginine cation-π interactions. Cell 173, 720.e15–734.e15. 10.1016/j.cell.2018.03.056 29677515 PMC5927716

[B324] Qin H. Lim L.-Z. Wei Y. Song J. (2014). TDP-43 N terminus encodes a novel ubiquitin-like fold and its unfolded form in equilibrium that can be shifted by binding to ssDNA. Proc. Natl. Acad. Sci. U S A 111, 18619–18624. 10.1073/pnas.1413994112 25503365 PMC4284588

[B325] Qiu H. Lee S. Shang Y. Wang W.-Y. Au K. F. Kamiya S.. (2014). ALS-associated mutation FUS-R521C causes DNA damage and RNA splicing defects. J. Clin. Invest. 124, 981–999. 10.1172/JCI72723 24509083 PMC3938263

[B326] Ramdzan Y. M. Polling S. Chia C. P. Z. Ng I. H. W. Ormsby A. R. Croft N. P.. (2012). Tracking protein aggregation and mislocalization in cells with flow cytometry. Nat. Methods 9, 467–470. 10.1038/nmeth.1930 22426490

[B327] Ramesh N. Pandey U. B. (2017). Autophagy dysregulation in ALS: when protein aggregates get out of hand. Front. Mol. Neurosci. 10:263. 10.3389/fnmol.2017.00263 28878620 PMC5572252

[B328] Rasouli S. Abdolvahabi A. Croom C. M. Plewman D. L. Shi Y. Ayers J. I.. (2017). Lysine acylation in superoxide dismutase-1 electrostatically inhibits formation of fibrils with prion-like seeding. J. Biol. Chem. 292, 19366–19380. 10.1074/jbc.M117.805283 28974578 PMC5702675

[B330] Ravits J. M. La Spada A. R. (2009). ALS motor phenotype heterogeneity, focality and spread: deconstructing motor neuron degeneration. Neurology 73, 805–811. 10.1212/WNL.0b013e3181b6bbbd 19738176 PMC2739608

[B329] Ravits J. Paul P. Jorg C. (2007). Focality of upper and lower motor neuron degeneration at the clinical onset of ALS. Neurology 68, 1571–1575. 10.1212/01.wnl.0000260965.20021.47 17485643

[B331] Ray S. S. Nowak R. J. Brown R. H. Jr. Lansbury P. T. Jr. (2005). Small-molecule-mediated stabilization of familial amyotrophic lateral sclerosis-linked superoxide dismutase mutants against unfolding and aggregation. Proc. Natl. Acad. Sci. U S A 102, 3639–3644. 10.1073/pnas.0408277102 15738401 PMC553303

[B332] Redler R. L. Wilcox K. C. Proctor E. A. Fee L. Caplow M. Dokholyan N. V. (2011). Glutathionylation at Cys-111 induces dissociation of wild type and FALS mutant SOD1 dimers. Biochemistry 50, 7057–7066. 10.1021/bi200614y 21739997 PMC3281512

[B333] Reilmann R. Mcgarry A. Grachev I. D. Savola J.-M. Borowsky B. Eyal E.. (2019). Safety and efficacy of pridopidine in patients with Huntington’s disease (PRIDE-HD): a phase 2, randomised, placebo-controlled, multicentre, dose-ranging study. Lancet Neurol. 18, 165–176. 10.1016/S1474-4422(18)30391-0 30563778

[B334] Renaud L. Picher-Martel V. Codron P. Julien J.-P. (2019). Key role of UBQLN2 in pathogenesis of amyotrophic lateral sclerosis and frontotemporal dementia. Acta Neuropathol. Commun. 7:103. 10.1186/s40478-019-0758-7 31319884 PMC6889556

[B335] Renton A. E. Chiò A. Traynor B. J. (2014). State of play in amyotrophic lateral sclerosis genetics. Nat. Neurosci. 17, 17–23. 10.1038/nn.3584 24369373 PMC4544832

[B336] Renton A. E. Majounie E. Waite A. Simón-Sánchez J. Rollinson S. Gibbs J. R.. (2011). A hexanucleotide repeat expansion in C9ORF72 is the cause of chromosome 9p21-linked ALS-FTD. Neuron 72, 257–268. 10.1016/j.neuron.2011.09.010 21944779 PMC3200438

[B337] Roberts B. R. Lim N. K. H. Mcallum E. J. Donnelly P. S. Hare D. J. Doble P. A.. (2014). Oral treatment with Cu(II; atsm) increases mutant SOD1 *in vivo* but protects motor neurons and improves the phenotype of a transgenic mouse model of amyotrophic lateral sclerosis. J. Neurosci. 34, 8021–8031. 10.1523/JNEUROSCI.4196-13.2014 24899723 PMC6608261

[B338] Robinson J. L. Geser F. Stieber A. Umoh M. Kwong L. K. Van Deerlin V. M.. (2013). TDP-43 skeins show properties of amyloid in a subset of ALS cases. Acta Neuropathol. 125, 121–131. 10.1007/s00401-012-1055-8 23124365 PMC3536927

[B339] Robinson M. B. Tidwell J. L. Gould T. Taylor A. R. Newbern J. M. Graves J.. (2005). Extracellular heat shock protein 70: a critical component for motoneuron survival. J. Neurosci. 25, 9735–9745. 10.1523/JNEUROSCI.1912-05.2005 16237177 PMC6725726

[B340] Rodriguez J. A. Valentine J. S. Eggers D. K. Roe J. A. Tiwari A. Brown R. H. Jr.. (2002). Familial amyotrophic lateral sclerosis-associated mutations decrease the thermal stability of distinctly metallated species of human copper/zinc superoxide dismutase. J. Biol. Chem. 277, 15932–15937. 10.1074/jbc.M112088200 11854285

[B341] Rosen D. R. Siddique T. Patterson D. Figlewicz D. A. Sapp P. Hentati A.. (1993). Mutations in Cu/Zn superoxide dismutase gene are associated with familial amyotrophic lateral sclerosis. Nature 362, 59–62. 10.1038/362059a0 8446170

[B342] Sackmann C. Sackmann V. Hallbeck M. (2020). TDP-43 is efficiently transferred between neuron-like cells in a manner enhanced by preservation of its N-terminus but independent of extracellular vesicles. Front. Neurosci. 14:540. 10.3389/fnins.2020.00540 32595443 PMC7301158

[B343] Safar J. Wille H. Itri V. Groth D. Serban H. Torchia M.. (1998). Eight prion strains have PrP(Sc) molecules with different conformations. Nat. Med. 4, 1157–1165. 10.1038/2654 9771749

[B344] Safar J. G. Xiao X. Kabir M. E. Chen S. Kim C. Haldiman T.. (2015). Structural determinants of phenotypic diversity and replication rate of human prions. PLoS Pathog. 11:e1004832. 10.1371/journal.ppat.1004832 25875953 PMC4397081

[B345] Şahin A. Held A. Bredvik K. Major P. Achilli T.-M. Kerson A. G.. (2017). Human SOD1 ALS mutations in a *Drosophila* knock-in model cause severe phenotypes and reveal dosage-sensitive gain- and loss-of-function components. Genetics 205, 707–723. 10.1534/genetics.116.190850 27974499 PMC5289846

[B346] Sahlholm K. Sijbesma J. W. A. Maas B. Kwizera C. Marcellino D. Ramakrishnan N. K.. (2015). Pridopidine selectively occupies sigma-1 rather than dopamine D2 receptors at behaviorally active doses. Psychopharmacology 232, 3443–3453. 10.1007/s00213-015-3997-8 26159455 PMC4537502

[B347] Saini A. Chauhan V. S. (2011). Delineation of the core aggregation sequences of TDP-43 C-terminal fragment. Chembiochem 12, 2495–2501. 10.1002/cbic.201100427 21905193

[B348] Saini A. Chauhan V. S. (2014). Self-assembling properties of peptides derived from TDP-43 C-terminal fragment. Langmuir 30, 3845–3856. 10.1021/la404710w 24559403

[B349] San Gil R. Ooi L. Yerbury J. J. Ecroyd H. (2017). The heat shock response in neurons and astroglia and its role in neurodegenerative diseases. Mol. Neurodegener. 12:65. 10.1186/s13024-017-0208-6 28923065 PMC5604514

[B350] Sangwan S. Zhao A. Adams K. L. Jayson C. K. Sawaya M. R. Guenther E. L.. (2017). Atomic structure of a toxic, oligomeric segment of SOD1 linked to amyotrophic lateral sclerosis (ALS). Proc. Natl. Acad. Sci. U S A 114, 8770–8775. 10.1073/pnas.1705091114. 28760994 PMC5565441

[B351] Sarkar M. Monteith W. B. Wang Y. Pielak G. J. (2012). Protein stability and macromolecular crowding. Biophys. J. 102:55a. 10.1016/j.bpj.2011.11.329 22954326

[B352] Schmidt M. Rohou A. Lasker K. Yadav J. K. Schiene-Fischer C. Fändrich M.. (2015). Peptide dimer structure in an Aβ(1–42) fibril visualized with cryo-EM. Proc. Natl. Acad. Sci. U S A 112, 11858–11863. 10.1073/pnas.1503455112 26351699 PMC4586870

[B353] Schmitz M. Cramm M. Llorens F. Müller-Cramm D. Collins S. Atarashi R.. (2016). The real-time quaking-induced conversion assay for detection of human prion disease and study of other protein misfolding diseases. Nat. Protoc. 11, 2233–2242. 10.1038/nprot.2016.120 27735933

[B354] Schütz B. Reimann J. Dumitrescu-Ozimek L. Kappes-Horn K. Landreth G. E. Schürmann B.. (2005). The oral antidiabetic pioglitazone protects from neurodegeneration and amyotrophic lateral sclerosis-like symptoms in superoxide dismutase-G93A transgenic mice. J. Neurosci. 25, 7805–7812. 10.1523/JNEUROSCI.2038-05.2005 16120782 PMC6725264

[B355] Scior A. Buntru A. Arnsburg K. Ast A. Iburg M. Juenemann K.. (2018). Complete suppression of Htt fibrilization and disaggregation of Htt fibrils by a trimeric chaperone complex. EMBO J. 37, 282–299. 10.15252/embj.201797212 29212816 PMC5770855

[B356] Sekhar A. Rumfeldt J. A. O. Broom H. R. Doyle C. M. Bouvignies G. Meiering E. M.. (2015). Thermal fluctuations of immature SOD1 lead to separate folding and misfolding pathways. eLife 4:e07296. 10.7554/eLife.07296 26099300 PMC4475725

[B357] Sekhar A. Rumfeldt J. A. O. Broom H. R. Doyle C. M. Sobering R. E. Meiering E. M.. (2016). Probing the free energy landscapes of ALS disease mutants of SOD1 by NMR spectroscopy. Proc. Natl. Acad. Sci. U S A 113, E6939–E6945. 10.1073/pnas.1611418113 27791136 PMC5111666

[B358] Senoo Y. Katoh K. Nakai Y. Hashimoto Y. Bando K. Teramoto S. (1988). Activity and stability of recombinant human superoxide dismutase in buffer solutions and hypothermic perfusates. Acta Med. Okayama 42, 169–174. 10.18926/AMO/31026 3041738

[B359] Sephton C. F. Cenik B. Cenik B. K. Herz J. Yu G. (2012). TDP-43 in central nervous system development and function: clues to TDP-43-associated neurodegeneration. Biol. Chem. 393, 589–594. 10.1515/hsz-2012-0115 22944662 PMC3537500

[B360] Shammas S. L. Waudby C. A. Wang S. Buell A. K. Knowles T. P. J. Ecroyd H.. (2011). Binding of the molecular chaperone αB-crystallin to Aβ amyloid fibrils inhibits fibril elongation. Biophys. J. 101, 1681–1689. 10.1016/j.bpj.2011.07.056 21961594 PMC3183811

[B361] Shelkovnikova T. A. Peters O. M. Deykin A. V. Connor-Robson N. Robinson H. Ustyugov A. A.. (2013). Fused in sarcoma (FUS) protein lacking nuclear localization signal (NLS) and major RNA binding motifs triggers proteinopathy and severe motor phenotype in transgenic mice. J. Biol. Chem. 288, 25266–25274. 10.1074/jbc.M113.492017 23867462 PMC3757190

[B362] Shenoy J. El Mammeri N. Dutour A. Berbon M. Saad A. Lends A.. (2020). Structural dissection of amyloid aggregates of TDP-43 and its C-terminal fragments TDP-35 and TDP-16. FEBS J. 287, 2449–2467. 10.1111/febs.15159 31782904

[B363] Shi Y. Rhodes N. R. Abdolvahabi A. Kohn T. Cook N. P. Marti A. A.. (2013). Deamidation of asparagine to aspartate destabilizes Cu, Zn superoxide dismutase, accelerates fibrillization and mirrors ALS-linked mutations. J. Am. Chem. Soc. 135, 15897–15908. 10.1021/ja407801x 24066782

[B364] Shibata N. Hirano A. Kobayashi M. Asayama K. Umahara T. Komori T.. (1993). Immunohistochemical demonstration of Cu/Zn superoxide dismutase in the spinal cord of patients with familial amyotro-phic lateral sclerosis. Acta Histochem. Cytochem. 26, 619–624.

[B365] Shimonaka S. Nonaka T. Suzuki G. Hisanaga S.-I. Hasegawa M. (2016). Templated aggregation of TAR DNA-binding protein of 43 kDa (TDP-43) by seeding with TDP-43 peptide fibrils. J. Biol. Chem. 291, 8896–8907. 10.1074/jbc.M115.713552 26887947 PMC4861459

[B366] Shinder G. A. Lacourse M. C. Minotti S. Durham H. D. (2001). Mutant Cu/Zn-superoxide dismutase proteins have altered solubility and interact with heat shock/stress proteins in models of amyotrophic lateral sclerosis. J. Biol. Chem. 276, 12791–12796. 10.1074/jbc.M010759200 11278741

[B367] Sievers S. A. Karanicolas J. Chang H. W. Zhao A. Jiang L. Zirafi O.. (2011). Structure-based design of non-natural amino-acid inhibitors of amyloid fibril formation. Nature 475, 96–100. 10.1038/nature10154 21677644 PMC4073670

[B368] Simpson J. H. Looger L. L. (2018). Functional imaging and optogenetics in *Drosophila*. Genetics 208, 1291–1309. 10.1534/genetics.117.300228 29618589 PMC5887132

[B369] Smith R. A. (2006). Antisense oligonucleotide therapy for neurodegenerative disease. J. Clin. Invest. 116, 2290–2296. 10.1172/JCI25424 16878173 PMC1518790

[B370] Son M. Puttaparthi K. Kawamata H. Rajendran B. Boyer P. J. Manfredi G.. (2007). Overexpression of CCS in G93A-SOD1 mice leads to accelerated neurological deficits with severe mitochondrial pathology. Proc. Natl. Acad. Sci. U S A 104, 6072–6077. 10.1073/pnas.0610923104 17389365 PMC1851618

[B371] Soon C. P. W. Donnelly P. S. Turner B. J. Hung L. W. Crouch P. J. Sherratt N. A.. (2011). Diacetylbis(*N*(4)-methylthiosemicarbazonato) copper(II) [CuII(atsm)] protects against peroxynitrite-induced nitrosative damage and prolongs survival in amyotrophic lateral sclerosis mouse model. J. Biol. Chem. 286, 44035–44044. 10.1074/jbc.M111.274407 22033929 PMC3243559

[B372] St Johnston D. (2002). The art and design of genetic screens: *Drosophila melanogaster*. Nat. Rev. Genet. 3, 176–188. 10.1038/nrg751 11972155

[B373] Stack C. Jainuddin S. Elipenahli C. Gerges M. Starkova N. Starkov A. A.. (2014). Methylene blue upregulates Nrf2/ARE genes and prevents tau-related neurotoxicity. Hum. Mol. Genet. 23, 3716–3732. 10.1093/hmg/ddu080 24556215 PMC4065148

[B374] Stathopulos P. B. Rumfeldt J. A. O. Scholz G. A. Irani R. A. Frey H. E. Hallewell R. A.. (2003). Cu/Zn superoxide dismutase mutants associated with amyotrophic lateral sclerosis show enhanced formation of aggregates *in vitro*. Proc. Natl. Acad. Sci. U S A 100, 7021–7026. 10.1073/pnas.1237797100 12773627 PMC165823

[B375] Stevens J. C. Chia R. Hendriks W. T. Bros-Facer V. Van Minnen J. Martin J. E.. (2010). Modification of superoxide dismutase 1 (SOD1) properties by a GFP Tag—implications for research into amyotrophic lateral sclerosis (ALS). PLoS One 5:e9541. 10.1371/journal.pone.0009541 20221404 PMC2833207

[B376] Strohäker T. Jung B. C. Liou S.-H. Fernandez C. O. Riedel D. Becker S.. (2019). Structural heterogeneity of α-synuclein fibrils amplified from patient brain extracts. Nat. Commun. 10:5535. 10.1038/s41467-019-13564-w 31797870 PMC6893031

[B377] Sulston J. E. Schierenberg E. White J. G. Thomson J. N. (1983). The embryonic cell lineage of the nematode *Caenorhabditis elegans*. Dev. Biol. 100, 64–119. 10.1016/0012-1606(83)90201-4 6684600

[B379] Sun Y. Arslan P. E. Won A. Yip C. M. Chakrabartty A. (2014). Binding of TDP-43 to the 3′UTR of its cognate mRNA enhances its solubility. Biochemistry 53, 5885–5894. 10.1021/bi500617x 25171271

[B378] Sun C.-S. Wang C. Y.-H. Chen B. P.-W. He R.-Y. Liu G. C.-H. Wang C.-H.. (2014). The influence of pathological mutations and proline substitutions in TDP-43 glycine-rich peptides on its amyloid properties and cellular toxicity. PLoS One 9:e103644. 10.1371/journal.pone.0103644 25090004 PMC4121164

[B380] Szczudlik A. Tomik B. Słowik A. Kasprzyk K. (1998). Assessment of the efficacy of treatment with pimozide in patients with amyotrophic lateral sclerosis. Introductory notes. Neurol. Neurochir. Pol. 32, 821–829. 9864711

[B381] Takahashi A. Nagao C. Murakami K. Kuroi K. Nakabayashi T. (2020). Effects of molecular crowding environment on the acquisition of toxic properties of wild-type SOD1. Biochim. Biophys. Acta Gen. Subj. 1864:129401. 10.1016/j.bbagen.2019.07.010 31348988

[B382] Taylor J. P. Brown R. H. Jr. Cleveland D. W. (2016). Decoding ALS: from genes to mechanism. Nature 539, 197–206. 10.1038/nature20413 27830784 PMC5585017

[B383] Telling G. C. Parchi P. Dearmond S. J. Cortelli P. Montagna P. Gabizon R.. (1996). Evidence for the conformation of the pathologic isoform of the prion protein enciphering and propagating prion diversity. Science 274, 2079–2082. 10.1126/science.274.5295.2079 8953038

[B384] Therrien M. Parker J. A. (2014). Worming forward: amyotrophic lateral sclerosis toxicity mechanisms and genetic interactions in *Caenorhabditis elegans*. Front. Genet. 5:85. 10.3389/fgene.2014.00085 24860590 PMC4029022

[B385] Thorpe J. R. Tang H. Atherton J. Cairns N. J. (2008). Fine structural analysis of the neuronal inclusions of frontotemporal lobar degeneration with TDP-43 proteinopathy. J. Neural Transm. 115, 1661–1671. 10.1007/s00702-008-0137-1 18974920 PMC2789307

[B386] Tokuda E. Nomura T. Ohara S. Watanabe S. Yamanaka K. Morisaki Y.. (2018). A copper-deficient form of mutant Cu/Zn-superoxide dismutase as an early pathological species in amyotrophic lateral sclerosis. Biochim. Biophys. Acta Mol. Basis Dis. 1864, 2119–2130. 10.1016/j.bbadis.2018.03.015 29551730

[B387] Trexler A. J. Rhoades E. (2012). N-Terminal acetylation is critical for forming α-helical oligomer of α-synuclein. Protein Sci. 21, 601–605. 10.1002/pro.2056 22407793 PMC3403458

[B388] Trist B. Hilton J. B. Crouch P. J. Hare D. J. Double K. L. (2020). Superoxide dismutase 1 in health and disease: how a front-line antioxidant becomes neurotoxic. Angew. Chem. Int. Ed Engl. [Epub ahead of print]. 10.1002/anie.202000451 32144830 PMC8247289

[B389] Tsaytler P. Harding H. P. Ron D. Bertolotti A. (2011). Selective inhibition of a regulatory subunit of protein phosphatase 1 restores proteostasis. Science 332, 91–94. 10.1126/science.1201396 21385720

[B390] Tsoi P. S. Choi K.-J. Leonard P. G. Sizovs A. Moosa M. M. Mackenzie K. R.. (2017). The N-terminal domain of ALS-linked TDP-43 assembles without misfolding. Angew. Chem. Int. Ed Engl. 56, 12590–12593. 10.1002/anie.201706769 28833982

[B391] Turner B. J. Atkin J. D. Farg M. A. Zang D. W. Rembach A. Lopes E. C.. (2005). Impaired extracellular secretion of mutant superoxide dismutase 1 associates with neurotoxicity in familial amyotrophic lateral sclerosis. J. Neurosci. 25, 108–117. 10.1523/JNEUROSCI.4253-04.2005 15634772 PMC6725218

[B392] Vaccaro A. Patten S. A. Aggad D. Julien C. Maios C. Kabashi E.. (2013). Pharmacological reduction of ER stress protects against TDP-43 neuronal toxicity *in vivo*. Neurobiol. Dis. 55, 64–75. 10.1016/j.nbd.2013.03.015 23567652

[B393] Vaccaro A. Patten S. A. Ciura S. Maios C. Therrien M. Drapeau P.. (2012a). Methylene blue protects against TDP-43 and FUS neuronal toxicity in *C. elegans* and *D. rerio*. PLoS One 7:e42117. 10.1371/journal.pone.0042117 22848727 PMC3407135

[B394] Vaccaro A. Tauffenberger A. Aggad D. Rouleau G. Drapeau P. Alex Parker J. (2012b). Mutant TDP-43 and FUS Cause age-dependent paralysis and neurodegeneration in *C. elegans*. PLoS One 7:e31321. 10.1371/journal.pone.0031321 22363618 PMC3283630

[B395] Vaccaro A. Tauffenberger A. Ash P. E. A. Carlomagno Y. Petrucelli L. Parker J. A. (2012c). TDP-1/TDP-43 regulates stress signaling and age-dependent proteotoxicity in *Caenorhabditis elegans*. PLoS Genet. 8:e1002806. 10.1371/journal.pgen.1002806 22792076 PMC3390363

[B397] Van Damme P. Robberecht W. Van Den Bosch L. (2017). Modelling amyotrophic lateral sclerosis: progress and possibilities. Dis. Model. Mech. 10, 537–549. 10.1242/dmm.029058 28468939 PMC5451175

[B398] Van Es M. A. Hardiman O. Chio A. Al-Chalabi A. Jeroen Pasterkamp R. Veldink J. H.. (2017). Amyotrophic lateral sclerosis. Lancet 390, 2084–2098. 10.1016/S0140-6736(17)31287-4 28552366

[B399] Vance C. Rogelj B. Hortobágyi T. De Vos K. J. Nishimura A. L. Sreedharan J.. (2009). Mutations in FUS, an RNA processing protein, cause familial amyotrophic lateral sclerosis type 6. Science 323, 1208–1211. 10.1126/science.1165942 19251628 PMC4516382

[B400] Vance C. Scotter E. L. Nishimura A. L. Troakes C. Mitchell J. C. Kathe C.. (2013). ALS mutant FUS disrupts nuclear localization and sequesters wild-type FUS within cytoplasmic stress granules. Hum. Mol. Genet. 22, 2676–2688. 10.1093/hmg/ddt117 23474818 PMC3674807

[B401] Vaquer-Alicea J. Diamond M. I. (2019). Propagation of protein aggregation in neurodegenerative diseases. Annu. Rev. Biochem. 88, 785–810. 10.1146/annurev-biochem-061516-045049 30917002

[B402] Vargas J. Y. Grudina C. Zurzolo C. (2019). The prion-like spreading of α-synuclein: from *in vitro* to *in vivo* models of Parkinson’s disease. Ageing Res. Rev. 50, 89–101. 10.1016/j.arr.2019.01.012 30690184

[B403] Vassall K. A. Stubbs H. R. Primmer H. A. Tong M. S. Sullivan S. M. Sobering R.. (2011). Decreased stability and increased formation of soluble aggregates by immature superoxide dismutase do not account for disease severity in ALS. Proc. Natl. Acad. Sci. U S A 108, 2210–2215. 10.1073/pnas.0913021108 21257910 PMC3038722

[B404] Vicencio E. Beltrán S. Labrador L. Manque P. Nassif M. Woehlbier U. (2020). Implications of selective autophagy dysfunction for ALS pathology. Cells 9:381. 10.3390/cells9020381 32046060 PMC7072226

[B1000] Vieira F. G. Hatzipetros T. Thompson K. Moreno A. J. Kidd J. D. Tassinari V. R.. (2017). CuATSM efficacy is independently replicated in a SOD1 mouse model of ALS while unmetallated ATSM therapy fails to reveal benefits. IBRO Reports 2, 47–53. 10.1016/j.ibror.2017.03.001 30135932 PMC6084867

[B405] Vígh L. Literáti P. N. Horváth I. Török Z. Balogh G. Glatz A.. (1997). Bimoclomol: a nontoxic, hydroxylamine derivative with stress protein-inducing activity and cytoprotective effects. Nat. Med. 3, 1150–1154. 10.1002/JLB.4MA0820-649R 9334730

[B406] Vilchez D. Saez I. Dillin A. (2014). The role of protein clearance mechanisms in organismal ageing and age-related diseases. Nat. Commun. 5:5659. 10.1038/ncomms6659 25482515

[B407] Vivoli Vega M. Nigro A. Luti S. Capitini C. Fani G. Gonnelli L.. (2019). Isolation and characterization of soluble human full-length TDP-43 associated with neurodegeneration. FASEB J. 33, 10780–10793. 10.1096/fj.201900474R 31287959

[B408] Walker A. K. Farg M. A. Bye C. R. Mclean C. A. Horne M. K. Atkin J. D. (2010). Protein disulphide isomerase protects against protein aggregation and is S-nitrosylated in amyotrophic lateral sclerosis. Brain 133, 105–116. 10.1093/brain/awp267 19903735

[B409] Walker A. K. Soo K. Y. Sundaramoorthy V. Parakh S. Ma Y. Farg M. A.. (2013). ALS-associated TDP-43 induces endoplasmic reticulum stress, which drives cytoplasmic TDP-43 accumulation and stress granule formation. PLoS One 8:e81170. 10.1371/journal.pone.0081170 24312274 PMC3843686

[B410] Walker D. S. Chew Y. L. Schafer W. R. (2019). “Genetics of behavior in *C. elegans*,” in The Oxford Handbook of Invertebrate Neurobiology, ed. Byrne J. H. (New York, NY: Oxford University Press), 150–170.

[B411] Wang A. Conicella A. E. Schmidt H. B. Martin E. W. Rhoads S. N. Reeb A. N.. (2018). A single N-terminal phosphomimic disrupts TDP-43 polymerization, phase separation and RNA splicing. EMBO J. 37:e97452. 10.15252/embj.201797452 29438978 PMC5830921

[B412] Wang J. Farr G. W. Hall D. H. Li F. Furtak K. Dreier L.. (2009a). An ALS-linked mutant SOD1 produces a locomotor defect associated with aggregation and synaptic dysfunction when expressed in neurons of *Caenorhabditis elegans*. PLoS Genet. 5:e1000350. 10.1371/journal.pgen.1000350 19165329 PMC2621352

[B413] Wang J. Farr G. W. Zeiss C. J. Rodriguez-Gil D. J. Wilson J. H. Furtak K.. (2009b). Progressive aggregation despite chaperone associations of a mutant SOD1-YFP in transgenic mice that develop ALS. Proc. Natl. Acad. Sci. U S A 106, 1392–1397. 10.1073/pnas.0813045106 19171884 PMC2631083

[B416] Wang Y.-T. Kuo P.-H. Chiang C.-H. Liang J.-R. Chen Y.-R. Wang S.. (2013). The truncated C-terminal RNA recognition motif of TDP-43 protein plays a key role in forming proteinaceous aggregates. J. Biol. Chem. 288, 9049–9057. 10.1074/jbc.M112.438564 23372158 PMC3610977

[B415] Wang W.-Y. Pan L. Su S. C. Quinn E. J. Sasaki M. Jimenez J. C.. (2013). Interaction of FUS and HDAC1 regulates DNA damage response and repair in neurons. Nat. Neurosci. 16, 1383–1391. 10.1038/nn.3514 24036913 PMC5564396

[B414] Wang P. Wander C. M. Yuan C.-X. Bereman M. S. Cohen T. J. (2017). Acetylation-induced TDP-43 pathology is suppressed by an HSF1-dependent chaperone program. Nat. Commun. 8:82. 10.1038/s41467-017-00088-4 28724966 PMC5517419

[B417] Watson M. D. Lee J. C. (2019). N-terminal acetylation affects α-synuclein fibril polymorphism. Biochemistry 58, 3630–3633. 10.1021/acs.biochem.9b00629 31424918 PMC6721997

[B418] Watson M. R. Lagow R. D. Xu K. Zhang B. Bonini N. M. (2008). A *Drosophila* model for amyotrophic lateral sclerosis reveals motor neuron damage by human SOD1. J. Biol. Chem. 283, 24972–24981. 10.1074/jbc.M804817200 18596033 PMC2529125

[B419] Watts J. C. Prusiner S. B. (2018). β-amyloid prions and the pathobiology of Alzheimer’s disease. Cold Spring Harb. Perspect. Med. 8:a023507. 10.1101/cshperspect.a023507 28193770 PMC5554751

[B420] Weisberg S. J. Lyakhovetsky R. Werdiger A.-C. Gitler A. D. Soen Y. Kaganovich D. (2012). Compartmentalization of superoxide dismutase 1 (SOD1G93A) aggregates determines their toxicity. Proc. Natl. Acad. Sci. U S A 109, 15811–15816. 10.1073/pnas.1205829109 22967507 PMC3465386

[B421] White J. G. Southgate E. Thomson J. N. Brenner S. (1986). The structure of the nervous system of the nematode *Caenorhabditis elegans*. Philos. Trans. R. Soc. Lond. B Biol. Sci. 314, 1–340. 10.1098/rstb.1986.0056 22462104

[B422] White M. A. Kim E. Duffy A. Adalbert R. Phillips B. U. Peters O. M.. (2018). TDP-43 gains function due to perturbed autoregulation in a Tardbp knock-in mouse model of ALS-FTD. Nat. Neurosci. 21, 552–563. 10.1038/s41593-018-0113-5 29556029 PMC5884423

[B423] White M. R. Mitrea D. M. Zhang P. Stanley C. B. Cassidy D. E. Nourse A.. (2019). C9orf72 Poly(PR) dipeptide repeats disturb biomolecular phase separation and disrupt nucleolar function. Mol. Cell 74, 713.e6–728.e6. 10.1016/j.molcel.2019.03.019 30981631 PMC6525025

[B424] Whiten D. R. San Gil R. Mcalary L. Yerbury J. J. Ecroyd H. Wilson M. R. (2016). Rapid flow cytometric measurement of protein inclusions and nuclear trafficking. Sci. Rep. 6:31138. 10.1038/srep31138 27516358 PMC4981889

[B425] Williams J. R. Trias E. Beilby P. R. Lopez N. I. Labut E. M. Samuel Bradford C.. (2016). Copper delivery to the CNS by CuATSM effectively treats motor neuron disease in SODG93A mice co-expressing the Copper-Chaperone-for-SOD. Neurobiol. Dis. 89, 1–9. 10.1016/j.nbd.2016.01.020 26826269 PMC4785045

[B426] Wright G. S. A. Antonyuk S. V. Hasnain S. S. (2019). The biophysics of superoxide dismutase-1 and amyotrophic lateral sclerosis. Q. Rev. Biophys. 52:e12. 10.1017/S003358351900012X 31760962

[B427] Wright G. S. A. Antonyuk S. V. Kershaw N. M. Strange R. W. Samar Hasnain S. (2013). Ligand binding and aggregation of pathogenic SOD1. Nat. Commun. 4:1758. 10.1038/ncomms2750 23612299 PMC3644087

[B428] Wright G. S. A. Watanabe T. F. Amporndanai K. Plotkin S. S. Cashman N. R. Antonyuk S. V.. (2020). Purification and structural characterization of aggregation-prone human TDP-43 involved in neurodegenerative diseases. iScience 23:101159. 10.1016/j.isci.2020.101159 32480125 PMC7262455

[B429] Wright P. D. Wightman N. Huang M. Weiss A. Sapp P. C. Cuny G. D.. (2012). A high-throughput screen to identify inhibitors of SOD1 transcription. Front. Biosci. 4, 2701–2708. 10.2741/e584 22652679 PMC4083181

[B430] Writing Group; Edaravone (MCI-186) ALS 19 Study Group. (2017). Safety and efficacy of edaravone in well defined patients with amyotrophic lateral sclerosis: a randomised, double-blind, placebo-controlled trial. Lancet Neurol. 16, 505–512. 10.1016/S1474-4422(17)30115-1 28522181

[B431] Xia G. Benmohamed R. Kim J. Arvanites A. C. Morimoto R. I. Ferrante R. J.. (2011). Pyrimidine-2,4,6-trione derivatives and their inhibition of mutant SOD1-dependent protein aggregation. toward a treatment for amyotrophic lateral sclerosis. J. Med. Chem. 54, 2409–2421. 10.1021/jm101549k 21375347 PMC3074252

[B433] Yang L. Gal J. Chen J. Zhu H. (2014). Self-assembled FUS binds active chromatin and regulates gene transcription. Proc. Natl. Acad. Sci. U S A 111, 17809–17814. 10.1073/pnas.1414004111 25453086 PMC4273402

[B432] Yang C. Tan W. Whittle C. Qiu L. Cao L. Akbarian S.. (2010). The C-terminal TDP-43 fragments have a high aggregation propensity and harm neurons by a dominant-negative mechanism. PLoS One 5:e15878. 10.1371/journal.pone.0015878 21209826 PMC3013128

[B434] Yerbury J. J. Farrawell N. E. McAlary L. (2020). Proteome homeostasis dysfunction: a unifying principle in ALS pathogenesis. Trends Neurosci. 43, 274–284. 10.1016/j.tins.2020.03.002 32353332

[B435] Yerbury J. J. Gower D. Vanags L. Roberts K. Lee J. A. Ecroyd H. (2013). The small heat shock proteins αB-crystallin and Hsp27 suppress SOD1 aggregation *in vitro*. Cell Stress Chaperones 18, 251–257. 10.1007/s12192-012-0371-1 22993064 PMC3581626

[B436] Yerbury J. J. Ooi L. Dillin A. Saunders D. N. Hatters D. M. Beart P. M.. (2016). Walking the tightrope: proteostasis and neurodegenerative disease. J. Neurochem. 137, 489–505. 10.1111/jnc.13575 26872075

[B437] Yoon G. Kim Y. K. Eom K. Na S. (2013). Relationship between disease-specific structures of amyloid fibrils and their mechanical properties. Appl. Phys. Lett. 102:011914. 10.1063/1.4774296

[B438] Yoshino H. (2019). Edaravone for the treatment of amyotrophic lateral sclerosis. Expert Rev. Neurother. 19, 185–193. 10.1080/14737175.2019.1581610 30810406

[B439] Yoshino H. Kimura A. (2006). Investigation of the therapeutic effects of edaravone, a free radical scavenger, on amyotrophic lateral sclerosis (Phase II study). Amyotroph. Lateral Scler. 7, 241–245. 10.1080/17482960600881870 17127563

[B440] Zacco E. Graña-Montes R. Martin S. R. De Groot N. S. Alfano C. Tartaglia G. G.. (2019). RNA as a key factor in driving or preventing self-assembly of the TAR DNA-binding protein 43. J. Mol. Biol. 431, 1671–1688. 10.1016/j.jmb.2019.01.028 30742796 PMC6461199

[B441] Zacco E. Martin S. R. Thorogate R. Pastore A. (2018). The RNA-recognition motifs of TAR DNA-binding protein 43 may play a role in the aberrant self-assembly of the protein. Front. Mol. Neurosci. 11:372. 10.3389/fnmol.2018.00372 30356856 PMC6190850

[B442] Zeineddine R. Whiten D. R. Farrawell N. E. Mcalary L. Hanspal M. A. Kumita J. R.. (2017). Flow cytometric measurement of the cellular propagation of TDP-43 aggregation. Prion 11, 195–204. 10.1080/19336896.2017.1314426 28486039 PMC5480386

[B446] Zhang Y. J. Gendron T. F. Grima J. C. Sasaguri H. Jansen-West K. Xu Y. F.. (2016). C9ORF72 poly(GA) aggregates sequester and impair HR23 and nucleocytoplasmic transport proteins. Nat. Neurosci. 19, 668–677. 10.1038/nn.4272 26998601 PMC5138863

[B444] Zhang T. Mullane P. C. Periz G. Wang J. (2011). TDP-43 neurotoxicity and protein aggregation modulated by heat shock factor and insulin/IGF-1 signaling. Hum. Mol. Genet. 20, 1952–1965. 10.1093/hmg/ddr076 21355045 PMC3080607

[B443] Zhang H. Tan C.-F. Mori F. Tanji K. Kakita A. Takahashi H.. (2008). TDP-43-immunoreactive neuronal and glial inclusions in the neostriatum in amyotrophic lateral sclerosis with and without dementia. Acta Neuropathol. 115, 115–122. 10.1007/s00401-007-0285-7 17786458

[B445] Zhang X. Wang F. Hu Y. Chen R. Meng D. Guo L.. (2020). *In vivo* stress granule misprocessing evidenced in a FUS knock-in ALS mouse model. Brain 143, 1350–1367. 10.1093/brain/awaa076 32358598

[B447] Zhang Y.-J. Xu Y.-F. Cook C. Gendron T. F. Roettges P. Link C. D.. (2009). Aberrant cleavage of TDP-43 enhances aggregation and cellular toxicity. Proc. Natl. Acad. Sci. U S A 106, 7607–7612. 10.1073/pnas.0900688106 19383787 PMC2671323

[B448] Zheng Z. Lauritzen J. S. Perlman E. Robinson C. G. Nichols M. Milkie D.. (2018). A complete electron microscopy volume of the brain of adult *Drosophila melanogaster*. Cell 174, 730.e22–743.e22. 10.1016/j.cell.2018.06.019 30033368 PMC6063995

[B449] Zhong Y. Wang J. Henderson M. J. Yang P. Hagen B. M. Siddique T.. (2017). Nuclear export of misfolded SOD1 mediated by a normally buried NES-like sequence reduces proteotoxicity in the nucleus. eLife 6:e23759. 10.7554/eLife.23759 28463106 PMC5449186

[B450] Zhou Y. Liu S. Liu G. Oztürk A. Hicks G. G. (2013). ALS-associated FUS mutations result in compromised FUS alternative splicing and autoregulation. PLoS Genet. 9:e1003895. 10.1371/journal.pgen.1003895 24204307 PMC3814325

[B451] Zhu L. Xu M. Yang M. Yang Y. Li Y. Deng J.. (2014). An ALS-mutant TDP-43 neurotoxic peptide adopts an anti-parallel β-structure and induces TDP-43 redistribution. Hum. Mol. Genet. 23, 6863–6877. 10.1093/hmg/ddu409 25113748 PMC4245047

